# Factors Influencing the Implementation of Remote Delivery Strategies for Non-Communicable Disease Care in Low- and Middle-Income Countries: A Narrative Review

**DOI:** 10.3389/phrs.2022.1604583

**Published:** 2022-06-27

**Authors:** Caroline Favas, Éimhín Ansbro, Evette Eweka, Gina Agarwal, Maria Lazo Porras, Ioanna Tsiligianni, Rajesh Vedanthan, Ruth Webster, Pablo Perel, Adrianna Murphy

**Affiliations:** ^1^ London School of Hygiene and Tropical Medicine, University of London, London, United Kingdom; ^2^ Grossman School of Medicine, New York University, New York, NY, United States; ^3^ Department of Family Medicine, McMaster University, Hamilton, ON, Canada; ^4^ Division of Tropical and Humanitarian Medicine, Geneva University Hospitals & University of Geneva, Geneva, Switzerland; ^5^ CRONICAS Centre of Excellence in Chronic Diseases, Universidad Peruana Cayetano Heredia, Lima, Peru; ^6^ Department of Social Medicine, Faculty of Medicine, University of Crete, Rethymno, Greece; ^7^ Centre for Health Economics Research and Evaluation, University of Technology Sydney, Sydney, NSW, Australia; ^8^ George Institute for Global Health, University of New South Wales, Newtown, NSW, Australia

**Keywords:** e-health, COVID-19, community, non-communicable diseases (NCDs), implementation, humanitarian, remote, continuity

## Abstract

**Objectives:** The COVID-19 pandemic has disrupted health care for non-communicable diseases (NCDs) and necessitated strategies to minimize contact with facilities. We aimed to examine factors influencing implementation of remote (non-facility-based) delivery approaches for people with hypertension and/or diabetes in low- and middle-income countries (LMICs), to inform NCD care delivery during health service disruption, including humanitarian crises.

**Methods:** Our narrative review used a hermeneutic and purposive approach, including primary studies conducted in LMICs, which assessed implementation factors influencing remote NCD care delivery. Results were analyzed using the Consolidated Framework for Implementation Research.

**Results:** Twenty-eight included studies revealed the strong influence of both internal organizational and broader contextual factors, such as community health worker policies or technological environment. Addressing patients’ specific characteristics, needs and resources was important for implementation success.

**Conclusion:** This review highlighted the multiple, complex, interdependent factors influencing implementation of remote NCD care in LMICs. Our findings may inform actors designing NCD care delivery in contexts where facility-based access is challenging. Implementation research is needed to evaluate context-adapted e-Health, community-based, and simplified clinical management strategies to facilitate remote NCD care.

## Introduction

Non-communicable diseases (NCDs) cause the greatest burden of mortality and ill-health worldwide [1]. Over three quarters of NCD deaths occur in low- and middle-income countries (LMICs) [2]. The growing burden of NCDs in LMICs, coupled with contextual and system-level challenges, has resulted in innovative approaches to healthcare delivery, including moving care closer to patients. Innovations have included shifting tasks to community health workers or community members, and utilization of technology, such as mobile phones [3–5].

Humanitarian crises, due to natural disaster or armed conflict, also disproportionally affect LMICs [6]. Such crises are characterized by “an exceptional and generalized threat to human life, health or subsistence”. They result in healthcare disruption and may exacerbate pre-existing poverty, inequality and poor access to basic services [7]. The growing global NCD burden has forced humanitarian actors to increase their focus on NCD care [8–11]. More recently, the COVID-19 pandemic has severely disrupted health systems globally, heightening the challenges experienced in many humanitarian contexts [9, 12, 13]. Since COVID-19 poses increased risk to people living with NCDs, they have been recommended to minimize interactions with others, including with health services [14, 15]. Thus, health care providers, including humanitarian actors, have had to adapt NCD service delivery to ensure continuity, while minimizing face-to-face patient contact [16, 17]. Anecdotally, adaptations to reduce facility-based attendance (referred to here as “remote” care), have included use of e-health, community-based strategies, and adaptations to medicines dispensing. These adaptations have been made in a largely reactive and unsystematic manner, and there is little evidence on how these delivery approaches might work in crisis-affected settings or on factors influencing their implementation [9, 11, 18]. Effective remote delivery approaches may prove useful during further waves of the COVID-19 pandemic, and in other settings of service disruption. Indeed, reduced facility-based attendance may also support continuity of care in LMIC and high-income country settings, outside of disruptive situations, where structural barriers limit health care access, and where a more patient-centered approach to care is desired [9, 11, 18].

To support continuity of care for people living with diabetes and/or hypertension (“DM/HTN”) in humanitarian crisis settings during COVID-19-related disruption, we designed a research project in partnership with humanitarian actors and implementation researchers. The project had two main components, an online survey and in-depth interviews with humanitarian actors to understand the disruption and adaptation of care for people with DM/HTN in humanitarian settings (to be published separately), and a narrative review of the literature to explore factors influencing implementation of delivery approaches focused on the remote provision of health care services for DM/HTN, reported here. In light of limited documented evidence from humanitarian crisis settings, we expanded our review to include relevant evidence from LMICs in general. NCD services in many LMICs face similar challenges to those faced in crises [19]. As such, our review will help inform the implementation of non-facility based NCD care by humanitarian actors but may be relevant to a wider audience in LMICs. Furthermore, although we aimed primarily to inform NCD care provision during the COVID-19 pandemic, the findings may be relevant in other settings where healthcare access is impeded by structural barriers or by disruption.

## Methods

### Study Design

We conducted a narrative review using a hermeneutic approach. We selected this approach since our aim was to create an “interpretive understanding” of the barriers and facilitators to successful implementation of selected delivery strategies, rather than to aggregate findings or summarize “facts” in response to a narrow research question, as would be typical of a conventional systematic review [20]. In addition, a narrative review was considered the most appropriate design because of the heterogeneous nature of the literature relating to our research question, the lack of standardized definitions and search terms for key eligibility criteria, and the iterative nature of narrative reviews, which best fit with our larger study aim.

### Scope of the Review

We included any primary research study conducted in LMICs, which assessed or evaluated implementation of our selected delivery strategies that eliminated or reduced facility-based attendance for patients with DM/HTN. The selected delivery approaches are detailed below. Table 1 summarizes the eligibility criteria, and definitions and examples of the selected delivery approaches are presented in Table 2.

**TABLE 1:** Study eligibility criteria (remote delivery strategies for non-communicable disease care in low- and middle-income countries) (United Kingdom 2022).

INCLUSION CRITERIA	EXCLUSION CRITERIA
**Setting**	
Low- and middle-income countries at the time of the study, and as defined by the world bank. this includes humanitarian crisis settings in low- and middle-income countries	High-income countries at the time of the study, and as defined by the world bank
**Intervention(s) or programme(s)**	
Intervention(s) or program(s) providing or supporting the provision of primary health care activities to adults living with DM/HTN^a^ designed to be delivered using any of the delivery approaches selected for the review (see Table 2)	Intervention(s) or program(s) providing or supporting the provision of specialized/secondary health care activities to adults living with DM/HTN, or exclusively for other non-communicable disease than DM/HTN, or Intervention(s) or program(s) providing or supporting the provision of primary health care activities for adults living with DM/HTN, but not using any of the delivery approaches selected for the review
The provision of primary health care activities to adults living with DM/HTN can be embedded into a larger intervention or program (e.g., non-communicable disease program or chronic disease program)	
**Type of research**	
All primary research studies (from grey or peer-reviewed literature) using qualitative, quantitative, or mixed methods approaches, and which include process evaluation/evaluation or assessment of implementation aspects of the selected delivery approaches	Editorials, commentaries, opinion pieces, conference abstracts, or studies that do not report process evaluation or evaluate implementation aspects of the selected delivery approaches
**Publication year**	
No restrictions	
**Language**	
Studies in English or French	Studies in any language other than English or French

a DM/HTN: diabetes and/or hypertension.

**TABLE 2:** Definition and examples of DM/HTN care delivery approaches selected for the review (remote delivery strategies for non-communicable disease care in low- and middle-income countries) (United Kingdom 2022).

DEFINITION	EXAMPLE
**E-health**	
Use of modern electronic information and communication technologies to support the remote provision of primary health care services and information at community level	SMS text messaging or IVR calls; remote advice/feedback; remote consultation; patient or provider electronic clinical decision support tools
**Community-based delivery strategies (including task sharing at community level)**	
Any health care activities implemented within the community and where the community is involved in the delivery of some aspects of care	Support for self-management; community/peer adherence groups; specific psychosocial/mental health support groups; disease monitoring
This includes sharing of health care tasks among a team of health care workers, especially enabling lay or mid-level health professionals to deliver clinical care traditionally performed by higher-level health care professionals at facility level AND enabling tasks to be performed at community level	
**Adaptation of provision of medicines**	
This includes adaptations in term of frequency and/or in terms of decentralization of the provision of medicines to the patient	Medication collection groups; drop off/pick up points at pharmacies; delivery through community health workers
**Simplification of clinical protocols**	
Simplification of treatment and monitoring guidelines	

Abbreviations: SMS, short message service; IVR, (automated) interactive voice response

#### Rationale for Eligibility Criteria

Evaluation of factors affecting implementation: To be eligible, studies needed to report on implementation factors, such as barriers or facilitators to implementation of the selected delivery strategies. Therefore, studies included process evaluation, seeking to understand the mechanisms inherent in effective implementation, the important factors (contexts, circumstances, and conditions) that determined if and how these mechanisms led to effective implementation and, finally, how those factors influenced the mechanisms.

Setting: In light of limited evidence on approaches to DM/HTN care in humanitarian crises, based on the findings of our recent systematic review on this topic [18], we broadened the scope of our review to all LMICs, including those affected by crises. The majority of crises occur in LMICs and the challenges facing NCD health care delivery in many LMICs, including resource scarcity, access disparity, lower health worker capacity, and lack of established NCD treatment programs and policies, are common to humanitarian crisis settings [19, 21].

Disease: We restricted our scope to the provision of DM/HTN care as they are the most common chronic conditions currently being addressed by humanitarian organizations. Additionally, they may act as tracer conditions to monitor the NCD delivery system for crisis-affected populations [22–29].

Delivery approaches: We focused on “remote” care delivery approaches. For the purposes of this review, “remote” provision of care is defined as health services delivered outside the primary health care facility, with the aim of minimizing patients’ contact with health facilities. In collaboration with an advisory committee composed of the largest humanitarian organizations, and drawing from the research team’s expertise, we identified and defined a range of delivery approaches supporting remote provision of DM/HTN care currently implemented in LMICs. These were selected because they were reported by humanitarian actors as the most frequently implemented responses to COVID-19-related disruption to health care delivery for NCDs. Our selected delivery approaches included: 1) e-health; 2) community-based delivery strategies, including task-sharing; 3) adaptation of medicines’ provision; and 4) simplification of protocols to minimize facility contact. To meet our criteria, each of these approaches had to have eliminated or reduced patient contact with primary care facilities.

#### Conceptual Framework

**FIGURE 1:**
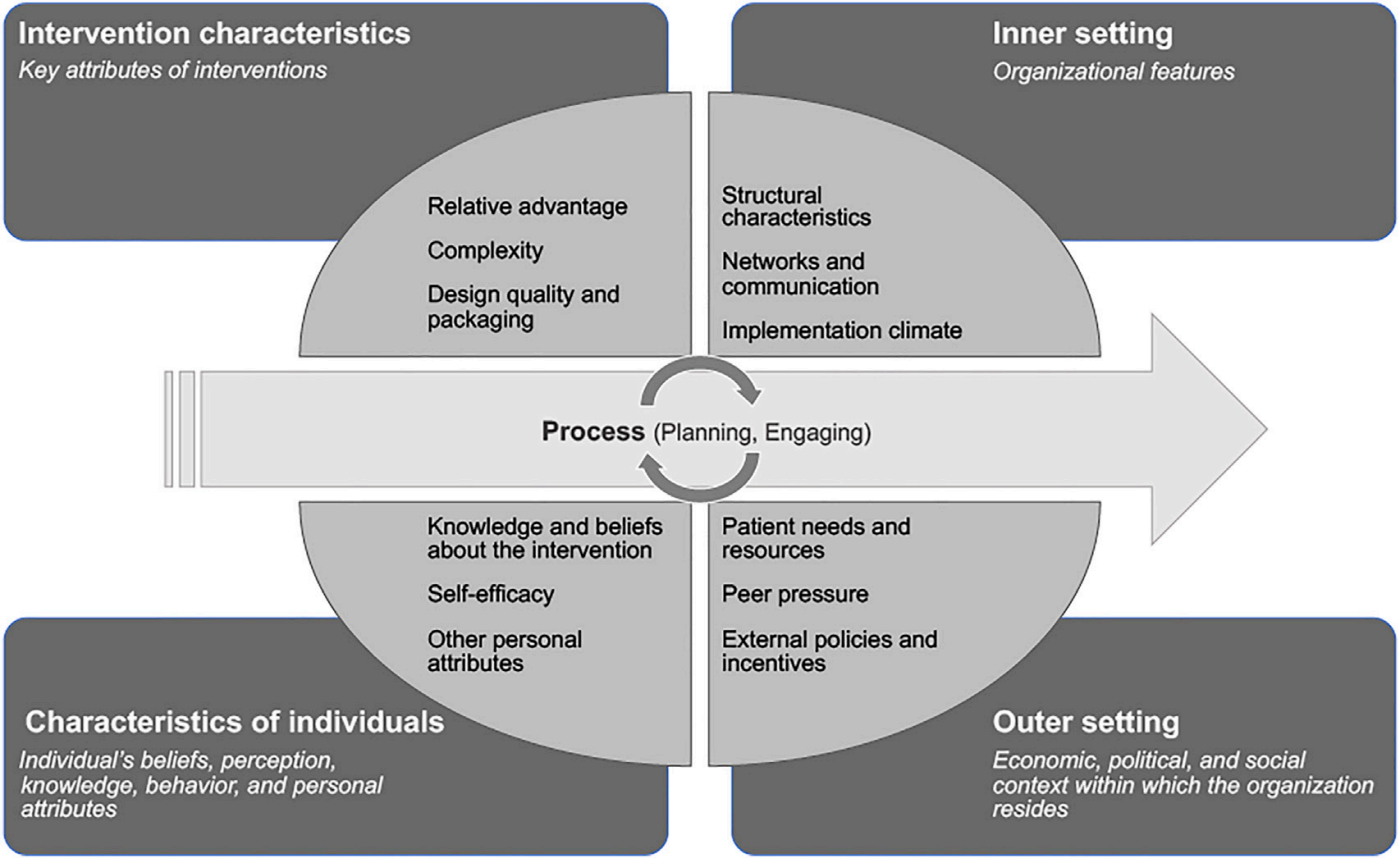
Consolidated Framework for Implementation Research (CFIR): domains and examples of related constructs—Adapted from Damschroder et al. [30] (remote delivery strategies for non-communicable disease care in low- and middle-income countries) (United Kingdom 2022).

To conduct, analyze and report the main findings of the review we used the Consolidated Framework for Implementation Research (CFIR) [30]. The CFIR is a theoretical framework with 39 constructs associated with effective implementation, which are organized under five major domains: Intervention Characteristic, Outer Setting, Inner Setting, Characteristics of Individuals, and Process (Figure 1) [30]. This widely-used, determinant framework helps to identify and understand drivers of effective implementation across multiple levels, while capturing the complexity within, and diversity across, various studies [30–32]. By providing a standardized definition of constructs and guidance for qualitative coding, the CFIR facilitated intuitive data extraction and analysis, while ensuring consistency and systemization in the process [32].

### Search Process

We applied a purposive search approach, drawing from three main sources to select eligible studies: 1) our recent systematic review of health care models for people with DM/HTN in humanitarian settings (Supplementary File S1); 2) our scoping review of studies of process evaluations for managing NCDs in primary care in LMICs (Supplementary File S2) [33] and 3) three reviews from the Global Alliance for Chronic Diseases network evaluating delivery strategies for the provision of DM/HTN care in LMICs [4, 34, 35]. We complemented this using ‘snowballing’ (i.e., pursuing references of references by hand or by means of citation-tracking databases) to identify additional studies of relevance. The references were managed using Mendeley, a bibliographic software management program [36].

### Screening Approach: Selection and Appraisal of Studies

After removal of duplicates, studies were selected through a 2-stage screening process by two independent reviewers (CF, EE). First, according to pre-defined eligibility criteria (Table 1), all records were systematically screened based on their titles, keywords, and abstracts, if available, and were rated: “Yes” (include), “No” (exclude), “Unsure” (not sure if it meets the criteria, or not enough information to decide) [37]. Only primary research studies in English or French were included; there were no restrictions by year of publication. Records rated “Yes” or “Unsure” progressed to full-text screening, while those with discordant ratings were discussed by the two reviewers, and disagreements were mediated by a third reviewer (AM, PP or EA). The full text of selected records was reviewed for relevance and quality of evidence. Ratings, rationales for decisions, and comments related to quality and relevance were documented.

### Data Extraction, Analysis, and Synthesis

Our Microsoft Excel-based data extraction table consisted of two sections: study characteristics and implementation aspects (Supplementary File S3). The latter was developed based on the CFIR framework, including all 5 domains and their respective constructs. For each article, data related to study characteristics were extracted by one reviewer (EE) while the implementation aspects were extracted by a second reviewer (CF). Using the CFIR lens, a thematic analysis and synthesis of evidence was conducted by CF. For each pre-selected delivery approach, implementation barriers and facilitators were mapped onto the appropriate CFIR domains and constructs, a coding framework was developed and adapted iteratively, and emerging themes were identified. Excerpts that best illustrated the emerging themes were identified. Extensive consultation was carried out within the research team to verify all relevant data were extracted, and to ensure quality, consistency, and coherence at all stages of data analysis. Differing views were resolved through discussion and consensus. For the “e-health” delivery approach, this iterative process led to the creation of two additional constructs under the outer setting domain that were not identified by CFIR (“socio-economic context” and “technological environment”).

## Results

**FIGURE 2:**
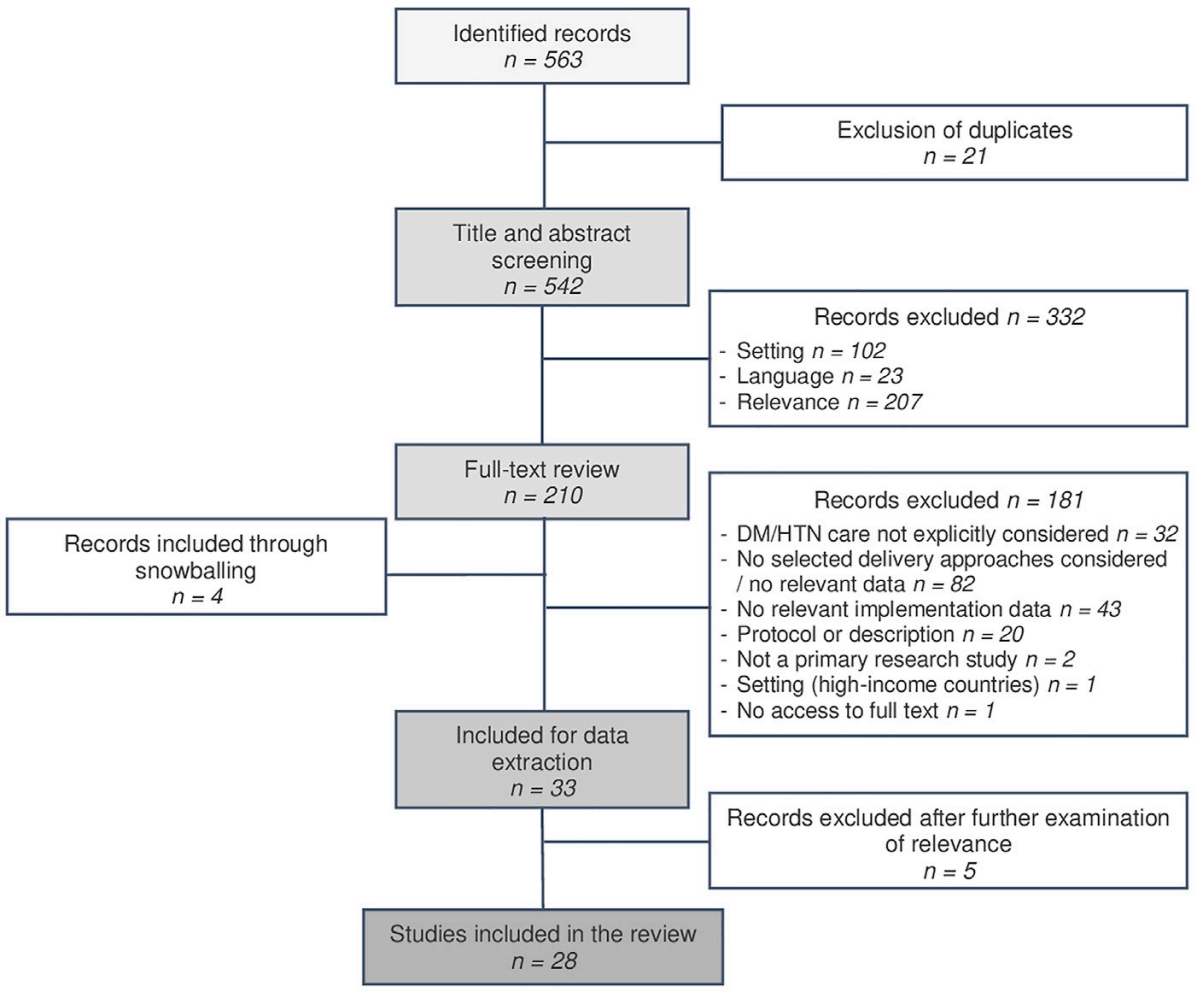
Flow chart of study selection (remote delivery strategies for non-communicable disease care in low- and middle-income countries) (United Kingdom 2022).

Twenty-eight papers met our eligibility criteria. Figure 2 provides an overview of the selection process, and a detailed summary of included studies is provided in Supplementary File S4.

### Study Characteristics

The included studies were published between 2011 and 2020. All concerned programs were implemented before the onset of the COVID-19 pandemic. Two studies related to the same research project [38, 39]. A total of 24 LMICs were represented across all continents. Most studies (*n* = 23) were exclusively conducted in middle-income countries (although one also included a high-income country). Among these, one also concerned a humanitarian setting [40]. Only 5 studies included a low-income country [38, 39, 41–43].

Most studies (*n* = 15) involved a mixed-methods design, and there was high heterogeneity in the objectives, reported outcomes, and data collection and analysis methods reported across studies. The objectives ranged from assessing feasibility, acceptability (mainly from a user perspective), utility, usability—with variability in the definitions of those terms—to broader objectives such as participants’ satisfaction with, or experience/perceptions of the intervention. Most also focused on clinical and/or behavioral effectiveness outcomes.

The majority of studies (*n* = 17) focused exclusively on DM [41–57], few (*n* = 6) focused exclusively on HTN [38, 39, 58–61], while two studies involved both conditions [40, 62]; one study also included coronary artery disease [63], a second study targeted chronic disease more generally, including infectious diseases such as HIV/AIDS or tuberculosis [64]; and another targeted depression among people living with DM and/or HTN [65].

Two of our selected delivery strategies featured most prominently, e-health and community-based delivery strategies. Half of the included studies (*n* = 14) involved e-health delivery strategies [38–40, 43, 47–51, 53–55, 57, 65], while the other half (*n* = 14) involved community-based delivery strategies [41, 42, 44–46, 52, 56, 58–64]. Among the latter, a number (*n* = 4) included shifting of clinical tasks to lay health workers at community level [58, 61, 62, 64]. Among our other selected delivery approaches, only one study included the adaptation of medicine provision [62], and none involved simplification of clinical protocols to minimize facility-based attendance.

The featured e-health strategies involved mobile phone-based health interventions (mHealth), such as short-message-service text messaging (SMS) or automated interactive voice response calls (IVR) [38–40, 43, 47, 49, 53–55, 57], telemonitoring [38, 39, 50, 55, 57], and web-based interventions, such as the use of a web application for education and support for self-management, or internet-based patient decision aids [48, 51, 65].

All studies concerning community-based delivery strategies involved self-management support provided by community health workers (CHWs) or nurses, or via peer-support groups or family members. Several (*n* = 5) studies included activities for disease monitoring or medication adherence [58, 61–64], and one included monthly home delivery of medicines by CHWs [62].

### Factors Affecting Implementation of Delivery Approaches

**FIGURE 3:**
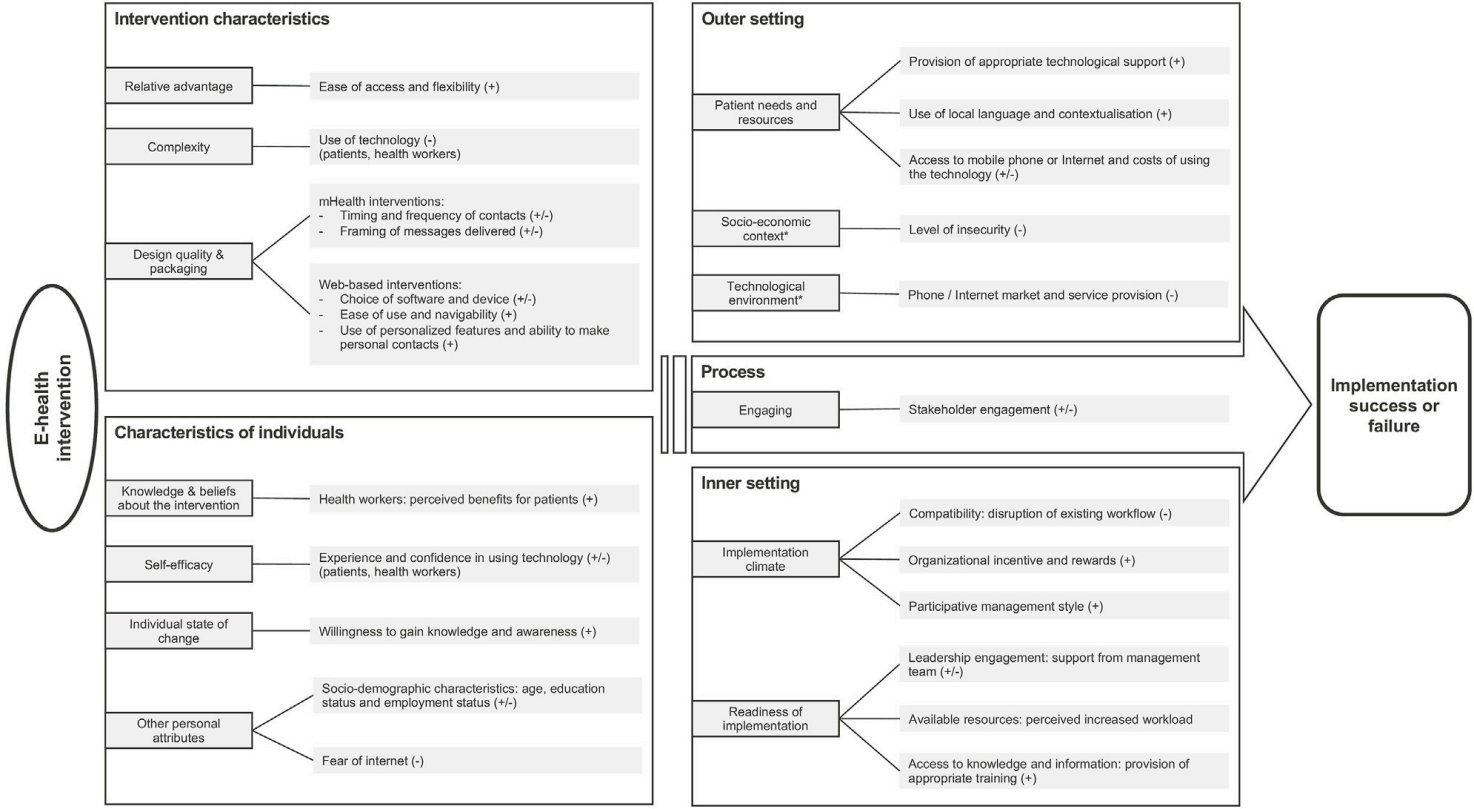
Summary of the factors influencing the implementation of interventions using e-health delivery approaches, based on Consolidated Framework for Implementation Research (CFIR). * New constructs generated inductively from the data. (+) facilitator (−) barrier (±) either facilitator or barrier depending on context (remote delivery strategies for non-communicable disease care in low- and middle-income countries) (United Kingdom 2022).

**FIGURE 4:**
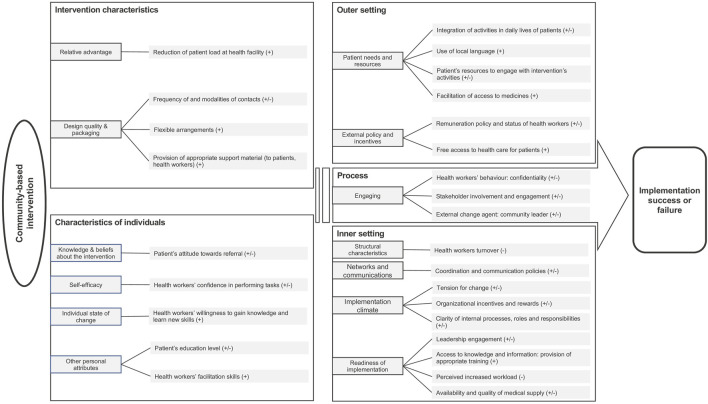
Summary of the factors influencing the implementation of interventions using community-based delivery approach, based on CFIR. (+) facilitator (−) barrier (±) either facilitator or barrier depending on context (remote delivery strategies for non-communicable diseases care in low- and middle-income countries) (United Kingdom 2022).

The factors affecting the implementation of e-health and community-based delivery approaches are presented according to the CFIR domains below and are summarized in Figures 3, 4. The CFIR domains include intervention characteristics, individual characteristics, outer setting, inner setting, and the implementation process (See Supplementary Files S5, S6 for coding frameworks). We did not report separately on our other selected delivery approaches, as only a single study (which was included in our reporting of community-based delivery strategies) referred to the adaptation of medicine provision, and, as mentioned, none involved simplification of protocols.

### E-Health

#### Intervention Characteristics: Technology Design and Perceived Complexity

Intervention characteristics were found to be a critical domain for effective implementation using e-health delivery approaches. Characteristics, such as ease of access (e.g., accessible from anywhere without requiring health center attendance) and flexibility in the use of the technology (available at any time) were considered facilitators of implementation [50, 51, 65], while the complexity and effort required to use a technology were perceived as barriers by both patients and health workers [50, 53, 55, 65]. Important design characteristics of mHealth interventions, such as SMS text messaging or IVR programs, included the timing and frequency of contacts and how the messages were framed [39, 40, 47–49, 54]; for example, one study reported that positively-framed messages were more persuasive than negatively-framed ones and that fear-based messages could unduly alarm people and cause more stress [39]. Regarding web-based interventions, characteristics influencing implementation included ease of use and navigability as well as incorporation of personalized features [38, 48, 51, 65]. A study evaluating a web-based decision aid for patients highlighted that users found it difficult to use features such as “drop-down” menus and a “hide-show” function, but particularly appreciated the personalized information provided through the application [50].

#### Characteristics of Individuals: Perceived Self-Efficacy and Socio-Demographic Characteristics

As reported in several studies, the participants’ lack of experience and knowledge in using technology (such as a smartphone application) could be a barrier to successful implementation, and could require external help, for example, from patient’s children [49, 50, 57, 65]. Moreover, patient’s age, education and employment status were identified as important socio-demographic characteristics to consider at the design and planning stage of an e-health intervention, as uptake was lower among older age groups and less educated people [40, 53, 57].

#### Outer Setting: Patient Needs and Technological Environment

Under the outer setting dimension, implementation success was influenced by access to technological support, which provided clear guidance and training on how to use the technology, or the possibility for patients to call for technical assistance [47, 65]. Furthermore, the use of local languages and contextualization—such as the adaptation of message content to local realities—were perceived as facilitators of implementation in several studies [39, 48, 54]. Unsurprisingly, the condition of local phone or internet markets, as well as the quality of service provision, were identified as significant barriers or facilitators [43, 47, 50, 53, 65]. For example, a study reported that, in Cambodia, the emergence of new telephone companies applying cheaper rates led participants to acquire new mobile phones and neglect the ones provided by the project [43]. While some studies estimated the cost of new technologies, such as mobile phones, none of the included studies evaluated whether this could be a barrier or facilitator to implementation.

#### Inner Setting: Implementation Climate and Readiness for Implementation

For health workers, the perceived disruption of workflow and increased workload induced by the intervention were commonly reported as barriers to its implementation [53, 65]. The provision of appropriate training and the level of support and supervision received by the leadership team either impeded or facilitated implementation [38, 65]. For example, in a study concerning a nurse-supported intervention delivered via a smartphone app, the nurses reported that the management team did not deliver on their agreement to adjust nurses’ work schedules to allow participation in the intervention, and, therefore, they found it difficult to accommodate the new activities within their existing workload [65].

#### Process: Stakeholders’ Engagement

Stakeholders’ engagement was found to be an important driver of successful implementation of e-health interventions, especially the consistency of health providers’ engagement and involvement throughout the implementation, and the level of support provided to the recipient of the e-health intervention (e.g., patients, relatives) [43, 53, 65]. One study reported a gradual decline in the number of SMS text messages sent to patients due to the difficulty of maintaining frequent meetings throughout the implementation period [43].

### Community-Based Delivery Strategies

#### Intervention Characteristics: Intervention Design and Perceived Relative Advantage

In a study involving monthly home visits by CHWs to provide support for self-management and home delivery of medication, the reduced patient load at the clinic was seen as a facilitator for the adoption of the intervention [62]. Factors relating to intervention design positively associated with implementation included flexibility in the arrangements—such as timing and frequency of patients visits by health staff or location of group meetings [44, 56]—as well as the provision of appropriate material to support implementation and uptake of the intervention by both health providers and patients. Examples included the provision of a mobile phone with prepaid network, the development of an information booklet, and the provision of a calendar to record patient’s medication adherence [42, 44, 58, 59].

#### Characteristics of Individuals: Perceived Self-Efficacy and Individual State of Change

Individual state of change is defined in the CFIR as “Characterization of the phase an individual is in, as he or she progresses toward skilled, enthusiastic, and sustained use of the intervention” [30]. Health workers’ confidence in performing the tasks, and their willingness to gain knowledge and learn new skills were identified as drivers of successful implementation of interventions involving community-based delivery strategies [58, 61].

#### Outer Setting: External Policy and Incentives, and Patient Needs and Resources

Under the CFIR construct “patient’s needs and resources”, factors such as ease of integration into patients’ daily lives and resources required to engage with the intervention were found to influence implementation [52, 56, 58]. For example, health workers in one study reported that the low attendance at group meetings, especially by housewives and farmers, was partly due to conflict with other daily activities and lack of transportation to reach the meeting venue [58]. The use of local language(s) was, again, perceived as a facilitator in several studies [41, 42, 46]. The status and remuneration of CHWs influenced implementation. One study highlighted that CHWs preferred being integrated in the health system and receiving a regular salary rather than financial incentives that were often delayed [58].

#### Inner Setting: Organizational Structure, Policies, Processes, and Resources

Provision of appropriate initial and refresher training, staff workload and the level of support provided by senior staff and leadership influenced the readiness for implementation and were, therefore, important drivers of its success [45, 58, 62, 63]. Furthermore, one study highlighted that implementation was strongly stymied by poor coordination and communication policies and poorly defined roles, responsibilities and internal processes (such as procurement processes) [62]. In the study including home delivery of medicines, the main reported barrier related to renewal of prescription at the health facility and the limited availability of the doctor responsible for it [62].

#### Process: Stakeholders’ Engagement

Stakeholders’ engagement with the intervention was a commonly cited influencing factor [41, 42, 56]. For example, the lack of available, motivated peers was reported as a challenge for the implementation of a peer-support program [41] while the involvement of community health professionals in peer group meetings was perceived as a facilitator in another study [56]. Community leaders’ participation in the intervention was also identified as important for implementation success. In a program for the prevention of diabetic foot complications, uptake was greatly improved by the participation of religious leaders in a foot care demonstration and in an informational video [52].

## Discussion

This narrative review illustrated factors influencing the implementation of e-health or community-based delivery approaches to reduce facility-based attendance for DM/HTN care in LMICs. Our findings revealed the strong influence that internal organizational context had on implementation, especially the role of staff. The need to examine the broader, external context was also clear, for example, the policies regarding community health workers for community-based interventions, or the technological environment relevant to e-health programs. Additionally, the influence of service users’ specific socio-demographic characteristics and the need to adapt both intervention design and implementation strategy to patients’ needs and resources were clear.

Our review highlights gaps in the literature. We found only one relevant study from a humanitarian crisis setting, and few from low-income settings. The importance of context in designing NCD care models for humanitarian settings has previously been highlighted [18]. However, implementation research to understand what works for whom and why in these complex settings is particularly sparse [18, 66]. We found no primary research studies relating to simplification of protocols, and only one study which involved the adaptation of medicines’ provision. Anecdotally, in response to Covid-19 pandemic restrictions and the increased clinical risk to NCD patients, some humanitarian actors reduced the frequency of laboratory testing and extended dispensing intervals to reduce NCD patient contact with facilities, but these adaptations have not been captured in the literature to date. Previous studies have called for the development of emergency plans to mitigate NCD service disruption, drawing on the experience of HIV care delivery in unstable humanitarian settings, and there are lessons to be learned from the pandemic response that may be applied to future instances of health service disruption, beyond humanitarian disasters [67].

Remote delivery of care has much broader and far-reaching implications than crisis-affected settings alone, and it is essential to learn from the rapid adaptations to NCD care delivery that took place in all settings in response to the COVID-19 pandemic. Interventions that minimize patients’ contact with health facilities, while ensuring continuity of care, will have important implications for “the new normal” after the pandemic response, for future health care disruptions, and for other settings where access to care is impeded by structural barriers, such as transport costs, distance and lack of health workforce. Remote care approaches may facilitate system adaptations, support patients with other challenges such as frailty, disability or poverty, and may allow for a more patient-centered approach to care [68, 69].

Although not explicitly explored in the included studies, this review shed some light on links across CFIR domains and relationships between factors, which could be leveraged to support effective implementation of our selected remote delivery approaches. Our findings suggest that health care providers’ perceptions of their own self-efficacy were fostered by the creation of a supportive learning environment, which in turn appeared to be influenced by the leadership’s level of engagement. Strong leadership and support seemed to be critical to building a positive and enabling working environment, for example, by facilitating changes to workflow and mobilizing resources. A coherent organizational structure, with existing coordination mechanisms and communication policies, supported the establishment of clear internal processes and roles and responsibilities. In relation to the outer setting (or broader context), the availability and stability of communication networks and the costs of phone and internet services contributed to the perception of a technology’s ease of use and usefulness. Our findings, thus, reflect the interdependency and potential synergy between the implementation factors we identified, and stresses that implementation of any innovative intervention is not a linear process but rather operates in a dynamic and complex system across multiple levels [31, 70].

We were unable to identify the mechanisms by which implementation factors operate or to establish their relative importance, as a realist review would seek to elucidate [71]. This may be partially due to the heterogeneity in focus, depth, and breadth of implementation aspects across studies and the inconsistency in reporting them, issues that have been raised elsewhere [70, 72]. In addition, in most included studies, implementation processes and related influencing factors were assessed anecdotally, and were not clearly distinguished from the intervention.

Future operational research is needed to strengthen the implementation of e-health- and community-based strategies minimizing face-to-face patient contact with primary care facilities in humanitarian and other LMIC settings. Research that serves to deepen our understanding of the relationships between factors affecting implementation outcomes, their relative importance, and the role local context plays in shaping those relationships is particularly warranted. Research documenting efforts to minimize facility-based contact through simplification of clinical management protocols or task-shifting elements of care to providers, peers or patients and families at the community level is also needed. In addition, using participatory methods, involving key stakeholders (patients, health care workers) in intervention design, has been found to be crucial to ensuring implementation success [73, 74]. However, this has not emerged as an influencing factor in our review, the main reason being that this was not evaluated in included studies. Therefore, we encourage including this aspect systematically in further evaluations of such strategies.

In the light of our study findings, we suggest designing and evaluating context-specific interventions to support remote DM/HTN that are supported by comprehensive analyses of patients’ socioeconomic and cultural circumstances, the health system, and the relevant technological and policy context. Examples of such interventions could include an e-health SMS-based tool, that is accessible, flexible, easy-to-use for people with limited education and experience of technology use, framed around positive messaging that is adapted to the realities of displacement, and peer-support groups for patients and carers to enhance diabetes self-management. Evaluating these interventions using a pragmatic implementation framework, such as the CIFR framework, would further our understanding of the factors essential for successful implementation. We suggest that reporting of implementation studies should be strengthened and standardized, potentially through the development of guidance, such as StaRI (Standards for Reporting Implementation Studies) [75].

This review benefitted from the strong collaboration between the authors and the advisory committee. The use of the CFIR allowed the identification of drivers of implementation success or failure in a systematic and structured way, which facilitated comparison across studies [30, 32]. Study limitations included the use of a purposive search strategy, which may have missed eligible studies and may limit the generalizability of our findings. However, our research question was not amenable to a standard systematic review approach and our search was strengthened by drawing from two systematic reviews covering NCD care in humanitarian and LMIC settings, respectively, whose search strategies would have covered studies eligible for inclusion in our own, and by using a snowballing approach and expert involvement. To mitigate the potential for implementation science frameworks to exclude relevant data that do not fit their constructs, we included themes generated inductively, for example scalability and long-term sustainability of interventions [32, 76].

This review provided insights relevant to policy makers, program managers, and other key decision-makers tailoring implementation strategies to support remote NCD care delivery where access to facilities is impeded, including crisis-affected settings. This is of particular relevance to the current COVID-19 pandemic response and to future health service disruptions, but may also serve to strengthen the development of patient-centered care in any context. Our findings highlighted the complexity of implementation processes, which are influenced by multiple interdependent factors in a dynamic way.
