# Supplementary material for: Factors Influencing the Implementation of Remote Delivery Strategies for Non-Communicable Disease Care in Low- and Middle-Income Countries: A Narrative Review
Source: Public Health Rev. 2022 Jun 27;43:1604583. doi: 10.3389/phrs.2022.1604583 (PMC9272771; doi:10.3389/phrs.2022.1604583)
Supplement: Supplementary file 1 [file DataSheet2.pdf]

Supplementary Material 2. Search strategy for referenced scoping review on process evaluation of complex interventions in chronic and neglected tropical diseases in low- and middle- income countries

Lazo-Porras M, Liu H, Miranda JJ, Moore G, Burri M, Chappuis F, et al. Process Evaluation of Complex Interventions in Chronic and Neglected Tropical Diseases in Low- and Middle-Income Countries-A Scoping Review Protocol. Syst Rev (2021) 10(1):244–7. doi:10.1186/s13643-021-01801-7

Search strategy

Pubmed

**Map the research on process evaluation in the areas of NCDs and NTDs to inform the gaps in the design and carrying out of this type of research in LMIC.**			
**Descriptor 1**	**Descriptor 2**	**Descriptor 3**	**Descriptor 4**
**process evaluation**	**NCDs and NTDs**	Low and middle income countries, according to The World Bank, which included 31 low-income economies, 47 lower-middle income economies and 60 upper-middle income economies https://datahelpdesk.worldbank.org/knowledgebase/articles/906519-world-bank-country-and-lending-groups<br><br>Middle income countries<br>Upper-middle income countries	RCT or CT (à voir)

Descriptor 1
process evaluation

MESH

("Outcome and Process Assessment (Health Care)"[Mesh]) AND ("Process Assessment (Health Care)"[Mesh] OR "Health Services Research"[Mesh])
21461 261119

Free terms

Process Assessments (Health Care) OR Process Assessment OR Process Assessments OR Process Measures OR Process Measure OR Outcome and Process Assessment OR Process evaluation OR fidelity OR acceptability

1305349 261119

1022731

MESH + Free terms

((("Outcome and Process Assessment (Health Care)"[Mesh]) OR ("Process Assessment (Health Care)"[Mesh] OR "Health Services Research"[Mesh]))) OR (Process Assessments (Health Care) OR Process Assessment OR Process Assessments OR Process Measures OR Process Measure OR Outcome and Process Assessment OR Process evaluation OR fidelity OR acceptability)

1305349 261119

Descriptor 2

NCDs and NTDs Non-communicable diseases and Neglected tropical diseases

MESH NCDs

((((((("Diabetes Mellitus"[Mesh] OR "Diabetes Mellitus, Type 2"[Mesh] OR "Diabetes Mellitus, Type 1"[Mesh] OR "Diabetes Complications"[Mesh] OR "Diabetes Mellitus, Insulin-Resistant, with Acanthosis Nigricans" [Supplementary Concept] OR "Diabetes, Gestational"[Mesh] OR "Diabetes Mellitus, Insulin-Dependent, 15" [Supplementary Concept] OR "Diabetes Mellitus, Insulin-Dependent, 6" [Supplementary Concept] OR "Diabetes Mellitus, Insulin-Dependent, 10" [Supplementary Concept] OR "Diabetes Mellitus, Insulin-Dependent, 17" [Supplementary Concept] OR "Diabetes Mellitus, Noninsulin-Dependent, 3" [Supplementary Concept] AND "Diabetes Mellitus, Insulin-Dependent, 18" [Supplementary Concept] OR "Diabetes Mellitus, Insulin-Dependent, 2" [Supplementary Concept] AND "Diabetes Mellitus, Insulin-Dependent, 3" [Supplementary Concept]) AND ("Hypertension"[Mesh] OR "Essential Hypertension"[Mesh] OR "Hypertension, Malignant"[Mesh])) OR "Cardiovascular Diseases"[Mesh]) OR ("Depression"[Mesh] OR "Depressive Disorder"[Mesh] OR "Depressive Disorder, Major"[Mesh] OR "Major Depressive Disorder 1" [Supplementary Concept] OR "Major Depressive Disorder 2" [Supplementary Concept])) OR "Renal Insufficiency, Chronic"[Mesh]) OR ("Noncommunicable Diseases"[Mesh] OR "Coronary Artery Disease"[Mesh])) OR ("Chronic Disease"[Mesh] OR "Multiple Chronic Conditions"[Mesh])) OR "Pulmonary Disease, Chronic Obstructive"[Mesh]

2852751 261119

MESH NTDs

((((((((((((((((((("Neglected Diseases") OR "Buruli Ulcer"[Mesh]) OR (Ulcer, Buruli) OR mycobacterium ulcerans OR "Chagas Disease"[Mesh]) OR "Dengue"[Mesh]) OR "Chikungunya Fever"[Mesh]) OR "Dracunculiasis"[Mesh]) OR ("Echinococcosis"[Mesh] OR "Echinococcosis, Pulmonary"[Mesh] OR "Echinococcosis, Hepatic"[Mesh] OR "Alveolar echinococcosis" [Supplementary Concept])) OR "Trypanosomiasis, African"[Mesh]) OR ("Leishmaniasis"[Mesh] OR "Leishmaniasis, Diffuse Cutaneous"[Mesh] OR "Leishmaniasis, Cutaneous"[Mesh] OR "Leishmaniasis, Visceral"[Mesh] OR "Leishmaniasis, Mucocutaneous"[Mesh])) OR ("Leprosy"[Mesh] OR "Leprosy, Multibacillary"[Mesh] OR "Leprosy, Paucibacillary"[Mesh] OR "Leprosy, Tuberculoid"[Mesh])) OR "Elephantiasis, Filarial"[Mesh]) OR ("Onchocerciasis"[Mesh] OR "Onchocerciasis, Ocular"[Mesh])) OR "Rabies"[Mesh]) OR ("Schistosomiasis"[Mesh] OR "Neuroschistosomiasis"[Mesh])) OR "Taeniasis"[Mesh]) OR "Neurocysticercosis"[Mesh]) OR ("Cysticercosis"[Mesh] OR "Submacular Cysticercosis" [Supplementary Concept])) OR "Trachoma"[Mesh]) OR "Yaws"[Mesh]) OR "Chromoblastomycosis"[Mesh]) OR "Scabies"[Mesh] OR "Snake Bites"[Mesh]

157305 261119

Free terms NTDs

Ulcer, Buruli OR mycobacterium ulcerans OR ((Disease, Chagas) OR american trypanosomiasis) OR trypansosoma cruzi OR (dengue) OR mosquito OR (chikungunya) OR Chikungunya virus OR (((dracunculiasis) OR guinea-worm disease) OR dracunculus medinensis) OR Dracunculiases ((((((((((echinococcosis) OR echinococcus granulosus) OR cystic echinococcosis) OR hydatidosis) OR hydatid disease) OR alveolar echinococcosis) OR echinococcus multilocularis) OR polycystic echinococcosis) OR echinococcus vogeli) OR unicystic echinococcosis) OR echinococcus oligarthrus) OR Echinococcoses OR ((((((((((foodborne trematodiases) OR trematode worms) OR flukes) OR freshwater snail) OR freshwater fish) OR clonorchis sinensis) OR opisthorchis viverrini) OR opisthorchis Felineus) OR fasciola hepatica) OR fasciola gigantica) OR paragonimus) OR Disease, Foodborne OR (((((((human african trypanosomiasis) OR sleeping sickness) OR trypanosoma brucei gambiense) OR tsetse flies) OR glossina genus) OR trypanosoma brucei rhodesiense) OR nagana) OR Trypanosomiases, African OR (((((((leishmaniasis) OR visceral leishmaniasis) OR cutaneous leishmaniasis) OR mucocutaneous leishmaniasis) OR kala-azar) OR leishmania parasites) OR phlebotomine sandflies) OR Leishmaniases OR ((((leprosy) OR hansen's disease) OR mycobacterium leprae) OR Leprosies) OR Hansen Disease OR (((((((((lymphatic filariasis) OR elephantiasis) OR nematodes) OR roundworms) OR wuchereria bancrofti) OR brugia malayi) OR brugia timori) OR culex) OR anopheles) OR aedes) OR Elephantiases, Filarial OR (((((onchocerciacis) OR river blindness) OR onchocerca volvulus) OR simulium) OR blackflies) OR Onchocerciases OR (rabies) OR Lyssa OR (((((((((((schistosomiasis) OR parasitic worms) OR blood flukes) OR trematode worms) OR Intestinal schistosomiasis) OR schistosoma mansoni) OR schistosoma japonicum) OR schistosoma mekongi) OR schistosoma guineensis) OR schistosoma intercalatum) OR urogenital schistosomiasis) OR schistosoma haematobium) OR Schistosomiases OR (((((((((soil-transmitted helminthiases) OR soil-transmitted helminth infection) OR roundworm) OR ascaris lumbricoides) OR whipworm) OR trichuris trichiura) OR hookworms) OR necator americanus) OR ancylostoma duodenale) OR Helminthiases) OR Infections, Nematomorpha OR (((((((((((taeniasis/cysticercosis) OR taeniasis) OR cysticercosis) OR tapeworms) OR taenia solium) OR pork tapeworm) OR taenia saginata) OR beef tapeworm) OR taenia asiatica) OR cysticerci) OR cysticercus) OR Cysticercoses OR ((trachoma) OR chlamydia trachomatis) OR Trachomas OR ((((endemic treponematoses) OR yaws) OR treponema pallidum) OR pertenue) OR Frambesia OR ((((Disease, Neglected) OR "neglected tropical disease") OR "tropical disease") OR "neglected disease")
424715 261119

Free terms NCDs

type 2 diabetes mellitus OR type 1 diabetes mellitus OR diabetes mellitus OR diabetes OR cardiovascular disease OR coronary heart disease OR heart failure OR depression OR chronic obstructive pulmonary disease OR copd OR chronic kidney disease OR chronic diseases OR non communicable diseases[all]
4084095 261119

NTD MESH + Free Terms

(((((((((((((((((((((("Neglected Diseases") OR "Buruli Ulcer"[Mesh]) OR (ulcer, buruli) OR mycobacterium ulcerans OR "Chagas Disease"[Mesh]) OR "Dengue"[Mesh]) OR "Chikungunya Fever"[Mesh]) OR "Dracunculiasis"[Mesh]) OR ("Echinococcosis"[Mesh] OR "Echinococcosis, Pulmonary"[Mesh] OR "Echinococcosis, Hepatic"[Mesh] OR "Alveolar echinococcosis"[Supplementary Concept])) OR "Trypanosomiasis, African"[Mesh]) OR ("Leishmaniasis"[Mesh] OR "Leishmaniasis, Diffuse Cutaneous"[Mesh] OR "Leishmaniasis, Cutaneous"[Mesh] OR "Leishmaniasis, Visceral"[Mesh] OR "Leishmaniasis, Mucocutaneous"[Mesh])) OR ("Leprosy"[Mesh] OR "Leprosy, Multibacillary"[Mesh] OR "Leprosy, Paucibacillary"[Mesh] OR "Leprosy, Tuberculoid"[Mesh])) OR "Elephantiasis, Filarial"[Mesh]) OR ("Onchocerciasis"[Mesh] OR "Onchocerciasis, Ocular"[Mesh])) OR "Rabies"[Mesh]) OR ("Schistosomiasis"[Mesh] OR "Neuroschistosomiasis"[Mesh])) OR "Taeniasis"[Mesh]) OR "Neurocysticercosis"[Mesh]) OR ("Cysticercosis"[Mesh] OR "Submacular Cysticercosis"[Supplementary Concept])) OR "Trachoma"[Mesh]) OR "Yaws"[Mesh]) OR "Chromoblastomycosis"[Mesh]) OR "Scabies"[Mesh] OR "Snake Bites"[Mesh])) OR (ulcer, buruli OR mycobacterium ulcerans OR ((disease, chagas) OR american trypanosomiasis) OR trypanosoma cruzi OR (dengue) OR mosquito OR (chikungunya) OR chikungunya virus OR (((dracunculiasis) OR guinea-worm disease) OR dracunculus medinensis) OR dracunculiasis (((((((((((echinococcosis) OR echinococcus granulosus) OR cystic echinococcosis) OR hydatidosis) OR hydatid disease) OR alveolar echinococcosis) OR echinococcus multilocularis) OR polycystic echinococcosis) OR echinococcus vogeli) OR unicystic echinococcosis) OR echinococcus oligarthrus) OR echinococcosis OR (((((((((((foodborne trematodiases) OR trematode worms) OR flukes) OR freshwater snail) OR freshwater fish) OR clonorchis sinensis) OR opisthorchis viverrini) OR opisthorchis felineus) OR fasciola hepatica) OR fasciola gigantica) OR paragonimus) OR disease, foodborne OR (((((((human african trypanosomiasis) OR sleeping sickness) OR trypanosoma brucei gambiense) OR tsetse flies) OR glossina genus) OR trypanosoma brucei rhodesiense) OR nagana) OR trypanosomiases, african OR (((((((leishmaniasis) OR visceral leishmaniasis) OR cutaneous leishmaniasis) OR mucocutaneous leishmaniasis) OR kala-azar) OR leishmania parasites) OR phlebotomine sandflies) OR leishmaniases OR ((((leprosy) OR hansen's disease) OR mycobacterium leprae) OR leprosy) OR hansen disease OR ((((((((((lymphatic filariasis) OR elephantiasis) OR nematodes) OR roundworms) OR wuchereria bancrofti) OR brugia malayi) OR brugia timori) OR culex) OR anopheles) OR aedes) OR elephantiasis, filarial OR (((((onchocerciasis) OR river blindness) OR onchocerca volvulus) OR simulium) OR blackflies) OR onchocerciasis OR (rabies) OR lyssa OR ((((((((((((schistosomiasis) OR parasitic worms) OR blood flukes) OR trematode worms) OR intestinal schistosomiasis) OR schistosoma mansoni) OR schistosoma japonicum) OR schistosoma mekongi) OR schistosoma guineensis) OR schistosoma intercalatum) OR urogenital schistosomiasis) OR schistosoma haematobium) OR schistosomiasis OR ((((((((((soil-transmitted helminthiases) OR soil-transmitted helminth infection) OR roundworm) OR ascaris lumbricoides) OR whipworm) OR trichuris trichiura) OR hookworms) OR necator americanus) OR ancylostoma duodenale) OR helminthiases) OR infections, nematomorpha OR (((((((((((taeniasis/cysticercosis) OR taeniasis) OR cysticercosis) OR tapeworms) OR taenia solium) OR pork tapeworm) OR taenia saginata) OR beef tapeworm) OR taenia asiatica) OR cysticerci) OR cysticercus) OR cysticercosis OR ((trachoma) OR chlamydia trachomatis) OR trachoma OR ((((endemic treponematoses) OR yaws) OR

treponema pallidum) OR pertenue) OR frambesia OR ((((disease, neglected) OR "neglected tropical disease") OR "tropical disease") OR "neglected disease"))
455858 261119

NCD MESH + Free Terms

((((((((("Diabetes Mellitus"[Mesh] OR "Diabetes Mellitus, Type 2"[Mesh] OR "Diabetes Mellitus, Type 1"[Mesh] OR "Diabetes Complications"[Mesh] OR "Diabetes Mellitus, Insulin-Resistant, with Acanthosis Nigricans" [Supplementary Concept] OR "Diabetes, Gestational"[Mesh] OR "Diabetes Mellitus, Insulin-Dependent, 15" [Supplementary Concept] OR "Diabetes Mellitus, Insulin-Dependent, 6" [Supplementary Concept] OR "Diabetes Mellitus, Insulin-Dependent, 10" [Supplementary Concept] OR "Diabetes Mellitus, Insulin-Dependent, 17" [Supplementary Concept] OR "Diabetes Mellitus, Noninsulin-Dependent, 3" [Supplementary Concept] AND "Diabetes Mellitus, Insulin-Dependent, 18" [Supplementary Concept] OR "Diabetes Mellitus, Insulin-Dependent, 2" [Supplementary Concept] AND "Diabetes Mellitus, Insulin-Dependent, 3" [Supplementary Concept]) AND ("Hypertension"[Mesh] OR "Essential Hypertension"[Mesh] OR "Hypertension, Malignant"[Mesh])) OR "Cardiovascular Diseases"[Mesh]) OR ("Depression"[Mesh] OR "Depressive Disorder"[Mesh] OR "Depressive Disorder, Major"[Mesh] OR "Major Depressive Disorder 1" [Supplementary Concept] OR "Major Depressive Disorder 2" [Supplementary Concept])) OR "Renal Insufficiency, Chronic"[Mesh]) OR ("Noncommunicable Diseases"[Mesh] OR "Coronary Artery Disease"[Mesh])) OR ("Chronic Disease"[Mesh] OR "Multiple Chronic Conditions"[Mesh])) OR "Pulmonary Disease, Chronic Obstructive"[Mesh])) OR (type 2 diabetes mellitus OR type 1 diabetes mellitus OR diabetes mellitus OR diabetes OR hypertension OR high blood pressure OR cardiovascular disease OR coronary heart disease OR heart failure OR depression OR chronic obstructive pulmonary disease OR copd OR chronic kidney disease OR chronic diseases OR non communicable diseases[all])

4261787 261119

NCDs + NTDs Mesh + Free Terms

((((((((((("Diabetes Mellitus"[Mesh] OR "Diabetes Mellitus, Type 2"[Mesh] OR "Diabetes Mellitus, Type 1"[Mesh] OR "Diabetes Complications"[Mesh] OR "Diabetes Mellitus, Insulin-Resistant, with Acanthosis Nigricans" [Supplementary Concept] OR "Diabetes, Gestational"[Mesh] OR "Diabetes Mellitus, Insulin-Dependent, 15" [Supplementary Concept] OR "Diabetes Mellitus, Insulin-Dependent, 6" [Supplementary Concept] OR "Diabetes Mellitus, Insulin-Dependent, 10" [Supplementary Concept] OR "Diabetes Mellitus, Insulin-Dependent, 17" [Supplementary Concept] OR "Diabetes Mellitus, Noninsulin-Dependent, 3" [Supplementary Concept] AND "Diabetes Mellitus, Insulin-Dependent, 18" [Supplementary Concept] OR "Diabetes Mellitus, Insulin-Dependent, 2" [Supplementary Concept] AND "Diabetes Mellitus, Insulin-Dependent, 3" [Supplementary Concept]) AND ("Hypertension"[Mesh] OR "Essential Hypertension"[Mesh] OR "Hypertension, Malignant"[Mesh])) OR "Cardiovascular Diseases"[Mesh]) OR ("Depression"[Mesh] OR "Depressive Disorder"[Mesh] OR "Depressive Disorder, Major"[Mesh] OR "Major Depressive Disorder 1" [Supplementary Concept] OR "Major Depressive Disorder 2" [Supplementary Concept])) OR "Renal Insufficiency, Chronic"[Mesh]) OR ("Noncommunicable Diseases"[Mesh] OR "Coronary Artery Disease"[Mesh])) OR ("Chronic Disease"[Mesh] OR "Multiple Chronic Conditions"[Mesh])) OR "Pulmonary Disease, Chronic Obstructive"[Mesh])) OR (type 2 diabetes mellitus OR type 1 diabetes mellitus OR diabetes mellitus OR diabetes OR hypertension OR high blood pressure OR

cardiovascular disease OR coronary heart disease OR heart failure OR depression OR chronic obstructive pulmonary disease OR copd OR chronic kidney disease OR chronic diseases OR non communicable diseases[all]))) OR ((((((((((((((((((((((("Neglected Diseases") OR "Buruli Ulcer"[Mesh]) OR (ulcer, buruli) OR mycobacterium ulcerans OR "Chagas Disease"[Mesh]) OR "Dengue"[Mesh]) OR "Chikungunya Fever"[Mesh]) OR "Dracunculiasis"[Mesh]) OR ("Echinococcosis"[Mesh] OR "Echinococcosis, Pulmonary"[Mesh] OR "Echinococcosis, Hepatic"[Mesh] OR "Alveolar echinococcosis"[Supplementary Concept])) OR "Trypanosomiasis, African"[Mesh]) OR ("Leishmaniasis"[Mesh] OR "Leishmaniasis, Diffuse Cutaneous"[Mesh] OR "Leishmaniasis, Cutaneous"[Mesh] OR "Leishmaniasis, Visceral"[Mesh] OR "Leishmaniasis, Mucocutaneous"[Mesh])) OR ("Leprosy"[Mesh] OR "Leprosy, Multibacillary"[Mesh] OR "Leprosy, Paucibacillary"[Mesh] OR "Leprosy, Tuberculoid"[Mesh])) OR "Elephantiasis, Filarial"[Mesh]) OR ("Onchocerciasis"[Mesh] OR "Onchocerciasis, Ocular"[Mesh])) OR "Rabies"[Mesh]) OR ("Schistosomiasis"[Mesh] OR "Neuroschistosomiasis"[Mesh])) OR "Taeniasis"[Mesh]) OR "Neurocysticercosis"[Mesh]) OR ("Cysticercosis"[Mesh] OR "Submacular Cysticercosis"[Supplementary Concept])) OR "Trachoma"[Mesh]) OR "Yaws"[Mesh]) OR "Chromoblastomycosis"[Mesh]) OR "Scabies"[Mesh] OR "Snake Bites"[Mesh])) OR (ulcer, buruli OR mycobacterium ulcerans OR ((disease, chagas) OR american trypanosomiasis) OR trypanosoma cruzi OR (dengue) OR mosquito OR (chikungunya) OR chikungunya virus OR (((dracunculiasis) OR guinea-worm disease) OR dracunculus medinensis) OR dracunculiasis (((((((((((echinococcosis) OR echinococcus granulosus) OR cystic echinococcosis) OR hydatidosis) OR hydatid disease) OR alveolar echinococcosis) OR echinococcus multilocularis) OR polycystic echinococcosis) OR echinococcus vogeli) OR unicystic echinococcosis) OR echinococcus oligarthrus) OR echinococcosis OR (((((((((((foodborne trematodiases) OR trematode worms) OR flukes) OR freshwater snail) OR freshwater fish) OR clonorchis sinensis) OR opisthorchis viverrini) OR opisthorchis felineus) OR fasciola hepatica) OR fasciola gigantica) OR paragonimus) OR disease, foodborne OR (((((((human african trypanosomiasis) OR sleeping sickness) OR trypanosoma brucei gambiense) OR tsetse flies) OR glossina genus) OR trypanosoma brucei rhodesiense) OR nagana) OR trypanosomiases, african OR (((((((leishmaniasis) OR visceral leishmaniasis) OR cutaneous leishmaniasis) OR mucocutaneous leishmaniasis) OR kala-azar) OR leishmania parasites) OR phlebotomine sandflies) OR leishmaniases OR ((((leprosy) OR hansen's disease) OR mycobacterium leprae) OR leprosy) OR hansen disease OR ((((((((((lymphatic filariasis) OR elephantiasis) OR nematodes) OR roundworms) OR wuchereria bancrofti) OR brugia malayi) OR brugia timori) OR culex) OR anopheles) OR aedes) OR elephantiasis, filarial OR (((((onchocerciasis) OR river blindness) OR onchocerca volvulus) OR simulium) OR blackflies) OR onchocerciasis OR (rabies) OR lyssa OR ((((((((((((schistosomiasis) OR parasitic worms) OR blood flukes) OR trematode worms) OR intestinal schistosomiasis) OR schistosoma mansoni) OR schistosoma japonicum) OR schistosoma mekongi) OR schistosoma guineensis) OR schistosoma intercalatum) OR urogenital schistosomiasis) OR schistosoma haematobium) OR schistosomiasis OR ((((((((((soil-transmitted helminthiases) OR soil-transmitted helminth infection) OR roundworm) OR ascaris lumbricoides) OR whipworm) OR trichuris trichiura) OR hookworms) OR necator americanus) OR ancylostoma duodenale) OR helminthiases) OR infections, nematomorpha OR (((((((((((taeniasis/cysticercosis) OR taeniasis) OR cysticercosis) OR tapeworms) OR taenia solium) OR pork tapeworm) OR taenia saginata) OR beef tapeworm) OR taenia asiatica) OR cysticerci) OR cysticercus) OR cysticercosis OR

((trachoma) OR chlamydia trachomatis) OR trachoma OR ((((endemic treponematoses) OR yaws) OR treponema pallidum) OR pertenue) OR frambesia OR ((((disease, neglected) OR "neglected tropical disease") OR "tropical disease") OR "neglected disease")))

4686883 261119

Descriptor 3

Low- and middle-income countries

MESH

(("Developing Countries"[Mesh]) OR "Afghanistan"[Mesh]) OR "Benin"[Mesh]) OR "Burkina Faso"[Mesh])) OR "Burundi"[Mesh]) OR "Central African Republic"[Mesh]) OR "Chad"[Mesh]) OR "Democratic Republic of the Congo"[Mesh]) OR "Ethiopia"[Mesh]) OR "Gambia"[Mesh]) OR "Guinea"[Mesh]) OR "Haiti"[Mesh]) OR "Democratic People's Republic of Korea"[Mesh]) OR "Liberia"[Mesh]) OR "Madagascar"[Mesh]) OR "Malawi"[Mesh]) OR "Nepal"[Mesh]) OR "Niger"[Mesh]) OR "Rwanda"[Mesh]) OR "Sierra Leone"[Mesh]) OR "Somalia"[Mesh]) OR "South Sudan"[Mesh]) OR "Syria"[Mesh]) OR "Tajikistan"[Mesh]) OR "Tanzania"[Mesh]) OR "Togo"[Mesh]) OR "Uganda"[Mesh]) OR "Yemen"[Mesh]) OR "Mali"[Mesh]) OR "Angola"[Mesh]) OR "Bangladesh"[Mesh]) OR "Bhutan"[Mesh]) OR "Bolivia"[Mesh]) OR "Cabo Verde"[Mesh]) OR "Cambodia"[Mesh]) OR "Cameroon"[Mesh]) OR "Comoros"[Mesh]) OR "Cote d'Ivoire"[Mesh]) OR "Djibouti"[Mesh]) OR "Egypt"[Mesh]) OR "El Salvador"[Mesh]) OR "Ghana"[Mesh]) OR "Honduras"[Mesh]) OR "India"[Mesh]) OR "Papua New Guinea"[Mesh]) OR "Indonesia"[Mesh]) OR "Kenya"[Mesh]) OR "Micronesia"[Mesh]) OR "Kyrgyzstan"[Mesh]) OR "Laos"[Mesh]) OR "Lesotho"[Mesh]) OR "Mauritania"[Mesh]) OR "Moldova"[Mesh]) OR "Mongolia"[Mesh]) OR "Morocco"[Mesh]) OR "Myanmar"[Mesh]) OR "Nicaragua"[Mesh]) OR "Nigeria"[Mesh]) OR "Pakistan"[Mesh]) OR "Philippines"[Mesh]) OR "Sao Tome and Principe"[Mesh]) OR "Senegal"[Mesh]) OR "Melanesia"[Mesh]) OR "Sudan"[Mesh]) OR "Swaziland"[Mesh]) OR "Timor-Leste"[Mesh]) OR "Tunisia"[Mesh]) OR "Ukraine"[Mesh]) OR "Uzbekistan"[Mesh]) OR "Vanuatu"[Mesh]) OR "Vietnam"[Mesh]) OR "Zambia"[Mesh]) OR "Zimbabwe"[Mesh]) OR "Middle East"[Mesh]) OR "Albania"[Mesh]) OR "Algeria"[Mesh]) OR "American Samoa"[Mesh]) OR "Argentina"[Mesh]) OR "Armenia"[Mesh]) OR "Azerbaijan"[Mesh]) OR "Republic of Belarus"[Mesh]) OR "Belize"[Mesh]) OR "Bosnia and Herzegovina"[Mesh]) OR "Botswana"[Mesh]) OR "Brazil"[Mesh]) OR "Bulgaria"[Mesh]) OR "China"[Mesh]) OR "Colombia"[Mesh]) OR "Costa Rica"[Mesh]) OR "Cuba"[Mesh]) OR "Dominica"[Mesh]) OR "Dominican Republic"[Mesh]) OR "Equatorial Guinea"[Mesh]) OR "Ecuador"[Mesh]) OR "Fiji"[Mesh]) OR "Gabon"[Mesh]) OR "Georgia (Republic)"[Mesh]) OR "Grenada"[Mesh]) OR "Guatemala"[Mesh]) OR "Guyana"[Mesh]) OR "Iran"[Mesh]) OR "Iraq"[Mesh]) OR "Jamaica"[Mesh]) OR "Jordan"[Mesh]) OR "Kazakhstan"[Mesh]) OR "Kosovo"[Mesh]) OR "Lebanon"[Mesh]) OR "Libya"[Mesh]) OR "Macedonia (Republic)"[Mesh]) OR "Malaysia"[Mesh]) OR "Indian Ocean Islands"[Mesh]) OR "Mauritius"[Mesh]) OR "Mexico"[Mesh]) OR "Montenegro"[Mesh]) OR "Namibia"[Mesh]) OR "Paraguay"[Mesh]) OR "Peru"[Mesh]) OR "Romania"[Mesh]) OR "Russia"[Mesh]) OR "Samoa"[Mesh]) OR "Serbia"[Mesh]) OR "Sri Lanka"[Mesh]) OR "South Africa"[Mesh]) OR "Saint Lucia"[Mesh]) OR "Saint Vincent and the Grenadines"[Mesh]) OR "Suriname"[Mesh]) OR "Thailand"[Mesh]) OR "Tonga"[Mesh]) OR "Turkey"[Mesh]) OR "Turkmenistan"[Mesh]) OR "Venezuela"[Mesh]

Free terms

Descriptor 4

Randomized controlled trials (RCTs) and non-RCTs

MESH

(("Clinical Trial" [Publication Type] OR "Clinical Trials as Topic"[Mesh] OR "Controlled Clinical Trial" [Publication Type] OR "Pragmatic Clinical Trial" [Publication Type] OR "Clinical Trial, Phase IV" [Publication Type] OR "Clinical Trial, Phase III" [Publication Type] OR "Clinical Trial, Phase II" [Publication Type] OR "Adaptive Clinical Trial" [Publication Type] OR "Non-Randomized Controlled Trials as Topic"[Mesh] OR "Adaptive Clinical Trials as Topic"[Mesh] OR "Pragmatic Clinical Trials as Topic"[Mesh] OR "Evaluation Studies" [Publication Type] OR "Randomized Controlled Trial" [Publication Type] OR "Validation Studies" [Publication Type]) OR ("Observational Studies as Topic"[Mesh] OR "Observational Study" [Publication Type])) OR ("Clinical Study" [Publication Type] OR "Clinical Studies as Topic"[Mesh]) OR randomized controlled trial[pt] OR controlled clinical trial[pt] OR randomized[tiab] OR placebo[tiab] OR drug therapy[sh] OR randomly[tiab] OR trial[tiab] OR groups[tiab] OR (randomized controlled trial[pt] OR controlled clinical trial[pt] OR randomized controlled trials[mh] OR random allocation[mh] OR double-blind method[mh] OR single-blind method[mh] OR clinical trial[pt] OR clinical trials[mh] OR "clinical trial"[tw] OR ((singl*[tw] OR doubl*[tw] OR trebl*[tw] OR tripl*[tw]) AND (mask*[tw] OR blind*[tw])) OR "latin square"[tw] OR placebos[mh] OR placebo*[tw] OR random*[tw] OR research design[mh:noexp] OR comparative study[pt] OR evaluation studies[pt] OR follow-up studies[mh] OR prospective studies[mh] OR cross-over studies[mh] OR control[tw] OR controll*[tw] OR prospectiv*[tw] OR volunteer*[tw]) NOT (animals[mh] NOT humans[mh])

8456387 261119

Free terms

randomized controlled trial[pt] OR controlled clinical trial[pt] OR randomized[tiab] OR placebo[tiab] OR drug therapy[sh] OR randomly[tiab] OR trial[tiab] OR groups[tiab] OR randomised controlled study OR randomised controlled trial OR rct OR non-rct OR comparative studies OR control group study OR control group trial OR controlled trial OR experimental studies OR feasibility studies OR field studies OR non experimental studies OR non experimental study OR nonexperimental studies OR nonexperimental study OR observation studies OR observation study OR observational studies OR panel study OR prevention trial OR preventive study OR preventive trial OR quality improvement studies OR quasiexperimental study OR replication studies OR trend studies OR twin studies OR twins study OR validation studies OR mixed method study

7124124 261119

MESH + Free Terms

((randomized controlled trial[pt] OR controlled clinical trial[pt] OR randomized[tiab] OR placebo[tiab] OR drug therapy[sh] OR randomly[tiab] OR trial[tiab] OR groups[tiab] OR randomised controlled study [Title/Abstract] OR randomised controlled trial [Title/Abstract] OR rct OR non-rct [Title/Abstract] OR comparative studies [Title/Abstract] OR control group study [Title/Abstract] OR control group trial [Title/Abstract] OR controlled trial [Title/Abstract] OR experimental studies [Title/Abstract] OR feasibility studies [Title/Abstract] OR field studies [Title/Abstract] OR non experimental studies [Title/Abstract] OR non experimental study [Title/Abstract] OR nonexperimental studies [Title/Abstract] OR nonexperimental study [Title/Abstract] OR observation studies [Title/Abstract] OR observation study [Title/Abstract] OR observational studies [Title/Abstract] OR panel study [Title/Abstract] OR prevention trial [Title/Abstract] OR preventive study [Title/Abstract] OR preventive trial [Title/Abstract] OR quality improvement studies [Title/Abstract] OR quasiexperimental study [Title/Abstract] OR replication studies [Title/Abstract] OR trend studies [Title/Abstract] OR twin studies [Title/Abstract] OR twins study [Title/Abstract] OR validation studies [Title/Abstract] OR mixed method study [Title/Abstract])) OR ((("Clinical Trial" [Publication Type] OR "Clinical Trials as Topic"[Mesh] OR "Controlled Clinical Trial" [Publication Type] OR "Pragmatic Clinical Trial" [Publication Type] OR "Clinical Trial, Phase IV" [Publication Type] OR "Clinical Trial, Phase III" [Publication Type] OR "Clinical Trial, Phase II" [Publication Type] OR "Adaptive Clinical Trial" [Publication Type] OR "Non-Randomized Controlled Trials as Topic"[Mesh] OR "Adaptive Clinical Trials as Topic"[Mesh] OR "Pragmatic Clinical Trials as Topic"[Mesh] OR "Evaluation Studies"

[Publication Type] OR "Randomized Controlled Trial" [Publication Type] OR "Validation Studies" [Publication Type]) OR ("Observational Studies as Topic"[Mesh] OR "Observational Study" [Publication Type])) OR ("Clinical Study" [Publication Type] OR "Clinical Studies as Topic"[Mesh]) OR randomized controlled trial[pt] OR controlled clinical trial[pt] OR randomized[tiab] OR placebo[tiab] OR drug therapy[sh] OR randomly[tiab] OR trial[tiab] OR groups[tiab] OR (randomized controlled trial[pt] OR controlled clinical trial[pt] OR randomized controlled trials[mh] OR random allocation[mh] OR double-blind method[mh] OR single-blind method[mh] OR clinical trial[pt] OR clinical trials[mh] OR "clinical trial"[tw] OR ((singl*[tw] OR doubl*[tw] OR trebl*[tw] OR tripl*[tw]) AND (mask*[tw] OR blind*[tw])) OR "latin square"[tw] OR placebos[mh] OR placebo*[tw] OR random*[tw] OR research design[mh:noexp] OR comparative study[pt] OR evaluation studies[pt] OR follow-up studies[mh] OR prospective studies[mh] OR cross-over studies[mh] OR control[tw] OR controll*[tw] OR prospectiv*[tw] OR volunteer*[tw]) NOT (animals[mh] NOT humans[mh]))

2602874 261119

NOT
fracture OR injury OR malignant neoplasm OR cancer surgery OR cardiovascular surgery OR stent OR surgery OR cancer OR stent OR (animals NOT humans)

ALL
((((((("Outcome and Process Assessment (Health Care)"[Mesh]) OR ("Process Assessment (Health Care)"[Mesh] OR "Health Services Research"[Mesh]))) OR (Process Assessments (Health Care) OR Process Assessment [tiab] OR Process Assessments [tiab] OR Process Measures [tiab] OR Process Measure [tiab] OR Outcome and Process Assessment [tiab] OR Process evaluation OR fidelity [tiab] OR implementation process))) AND ((((randomized controlled trial[pt] OR controlled clinical trial[pt] OR randomized[tiab] OR placebo[tiab] OR drug therapy[sh] OR randomly[tiab] OR trial[tiab] OR groups[tiab] OR randomised controlled study [tiab] OR randomised controlled trial [tiab] OR rct [tiab] OR non-rct [tiab] OR comparative studies [tiab] OR control group study [tiab] OR control group trial [tiab] OR controlled trial [tiab] OR experimental studies [tiab] OR feasibility studies [tiab] OR field studies [tiab] OR non experimental studies [tiab] OR non experimental study [tiab] OR nonexperimental studies [tiab] OR nonexperimental study [tiab] OR observation studies [tiab] OR observation study [tiab] OR observational studies [tiab] OR prevention trial [tiab] OR preventive study [tiab] OR preventive trial [tiab] OR quality improvement studies [tiab] OR quasiexperimental study [tiab] OR validation studies [tiab] OR mixed method study [tiab])) OR ((("Clinical Trial" [Publication Type] OR "Clinical Trials as Topic"[Mesh] OR "Controlled Clinical Trial" [Publication Type] OR "Pragmatic Clinical Trial" [Publication Type] OR "Clinical Trial, Phase IV" [Publication Type] OR "Clinical Trial, Phase III" [Publication Type] OR "Clinical Trial, Phase II" [Publication Type] OR "Adaptive Clinical Trial" [Publication Type] OR "Non-Randomized Controlled Trials as Topic"[Mesh] OR "Adaptive Clinical Trials as Topic"[Mesh] OR "Pragmatic Clinical Trials as Topic"[Mesh] OR "Evaluation Studies" [Publication Type] OR "Randomized Controlled Trial" [Publication Type] OR "Validation Studies" [Publication Type]) OR ("Observational Studies as Topic"[Mesh] OR "Observational Study" [Publication Type])) OR ("Clinical Study" [Publication Type] OR "Clinical Studies as Topic"[Mesh]) OR randomized controlled trial[pt] OR controlled clinical trial[pt] OR randomized[tiab] OR placebo[tiab] OR drug therapy[sh] OR randomly[tiab] OR trial[tiab] OR groups[tiab] OR (randomized controlled trial[pt] OR controlled

clinical trial[pt] OR randomized controlled trials[mh] OR random allocation[mh] OR double-blind method[mh] OR single-blind method[mh] OR clinical trial[pt] OR clinical trials[mh] OR "clinical trial"[tw] OR comparative study[pt] OR evaluation studies[pt] OR follow-up studies[mh] OR prospective studies[mh] OR cross-over studies[mh] OR control[tw]) NOT (animals[mh] NOT humans[mh]))))) AND
(((
((((((((((((((((((((((((((("Developing Countries"[Mesh]) OR "Afghanistan"[Mesh]) OR "Benin"[Mesh]) OR "Burkina Faso"[Mesh])) OR "Burundi"[Mesh]) OR "Central African Republic"[Mesh]) OR "Chad"[Mesh]) OR "Democratic Republic of the Congo"[Mesh]) OR "Ethiopia"[Mesh]) OR "Gambia"[Mesh]) OR "Guinea"[Mesh]) OR "Haiti"[Mesh]) OR "Democratic People's Republic of Korea"[Mesh]) OR "Liberia"[Mesh]) OR "Madagascar"[Mesh]) OR "Malawi"[Mesh]) OR "Nepal"[Mesh]) OR "Niger"[Mesh]) OR "Rwanda"[Mesh]) OR "Sierra Leone"[Mesh]) OR "Somalia"[Mesh]) OR "South Sudan"[Mesh]) OR "Syria"[Mesh]) OR "Tajikistan"[Mesh]) OR "Tanzania"[Mesh]) OR "Togo"[Mesh]) OR "Uganda"[Mesh]) OR "Yemen"[Mesh]) OR "Mali"[Mesh]) OR "Angola"[Mesh]) OR "Bangladesh"[Mesh]) OR "Bhutan"[Mesh]) OR "Bolivia"[Mesh]) OR "Cabo Verde"[Mesh]) OR "Cambodia"[Mesh]) OR "Cameroon"[Mesh]) OR "Comoros"[Mesh]) OR "Cote d'Ivoire"[Mesh]) OR "Djibouti"[Mesh]) OR "Egypt"[Mesh]) OR "El Salvador"[Mesh]) OR "Ghana"[Mesh]) OR "Honduras"[Mesh]) OR "India"[Mesh]) OR "Papua New Guinea"[Mesh]) OR "Indonesia"[Mesh]) OR "Kenya"[Mesh]) OR "Micronesia"[Mesh]) OR "Kyrgyzstan"[Mesh]) OR "Laos"[Mesh]) OR "Lesotho"[Mesh]) OR "Mauritania"[Mesh]) OR "Moldova"[Mesh]) OR "Mongolia"[Mesh]) OR "Morocco"[Mesh]) OR "Myanmar"[Mesh]) OR "Nicaragua"[Mesh]) OR "Nigeria"[Mesh]) OR "Pakistan"[Mesh]) OR "Philippines"[Mesh]) OR "Sao Tome and Principe"[Mesh]) OR "Senegal"[Mesh]) OR "Melanesia"[Mesh]) OR "Sudan"[Mesh]) OR "Swaziland"[Mesh]) OR "Timor-Leste"[Mesh]) OR "Tunisia"[Mesh]) OR "Ukraine"[Mesh]) OR "Uzbekistan"[Mesh]) OR "Vanuatu"[Mesh]) OR "Vietnam"[Mesh]) OR "Zambia"[Mesh]) OR "Zimbabwe"[Mesh]) OR "Middle East"[Mesh]) OR "Albania"[Mesh]) OR "Algeria"[Mesh]) OR "American Samoa"[Mesh]) OR "Argentina"[Mesh]) OR "Armenia"[Mesh]) OR "Azerbaijan"[Mesh]) OR "Republic of Belarus"[Mesh]) OR "Belize"[Mesh]) OR "Bosnia and Herzegovina"[Mesh]) OR "Botswana"[Mesh]) OR "Brazil"[Mesh]) OR "Bulgaria"[Mesh]) OR "China"[Mesh]) OR "Colombia"[Mesh]) OR "Costa Rica"[Mesh]) OR "Cuba"[Mesh]) OR "Dominica"[Mesh]) OR "Dominican Republic"[Mesh]) OR "Equatorial Guinea"[Mesh]) OR "Ecuador"[Mesh]) OR "Fiji"[Mesh]) OR "Gabon"[Mesh]) OR "Georgia (Republic)"[Mesh]) OR "Grenada"[Mesh]) OR "Guatemala"[Mesh]) OR "Guyana"[Mesh]) OR "Iran"[Mesh]) OR "Iraq"[Mesh]) OR "Jamaica"[Mesh]) OR "Jordan"[Mesh]) OR "Kazakhstan"[Mesh]) OR "Kosovo"[Mesh]) OR "Lebanon"[Mesh]) OR "Libya"[Mesh]) OR "Macedonia (Republic)"[Mesh]) OR "Malaysia"[Mesh]) OR "Indian Ocean Islands"[Mesh]) OR "Mauritius"[Mesh]) OR "Mexico"[Mesh]) OR "Montenegro"[Mesh]) OR "Namibia"[Mesh]) OR "Paraguay"[Mesh]) OR "Peru"[Mesh]) OR "Romania"[Mesh]) OR "Russia"[Mesh]) OR "Samoa"[Mesh]) OR "Serbia"[Mesh]) OR "Sri Lanka"[Mesh]) OR "South Africa"[Mesh]) OR "Saint Lucia"[Mesh]) OR "Saint Vincent and the Grenadines"[Mesh]) OR "Suriname"[Mesh]) OR "Thailand"[Mesh]) OR "Tonga"[Mesh]) OR "Turkey"[Mesh]) OR "Turkmenistan"[Mesh]) OR "Venezuela"[Mesh]))) AND (((((((((((("Diabetes Mellitus"[Mesh] OR "Diabetes Mellitus, Type 2"[Mesh] OR "Diabetes Mellitus, Type 1"[Mesh] OR "Diabetes Complications"[Mesh] OR "Diabetes Mellitus, Insulin-

Resistant, with Acanthosis Nigricans" [Supplementary Concept] OR "Diabetes, Gestational"[Mesh] OR "Diabetes Mellitus, Insulin-Dependent, 15" [Supplementary Concept] OR "Diabetes Mellitus, Insulin-Dependent, 6" [Supplementary Concept] OR "Diabetes Mellitus, Insulin-Dependent, 10" [Supplementary Concept] OR "Diabetes Mellitus, Insulin-Dependent, 17" [Supplementary Concept] OR "Diabetes Mellitus, Noninsulin-Dependent, 3" [Supplementary Concept] AND "Diabetes Mellitus, Insulin-Dependent, 18" [Supplementary Concept] OR "Diabetes Mellitus, Insulin-Dependent, 2" [Supplementary Concept] AND "Diabetes Mellitus, Insulin-Dependent, 3" [Supplementary Concept]) AND ("Hypertension"[Mesh] OR "Essential Hypertension"[Mesh] OR "Hypertension, Malignant"[Mesh])) OR "Cardiovascular Diseases"[Mesh]) OR ("Depression" [Majr] OR "Depressive Disorder"[Mesh] OR "Depressive Disorder, Major"[Mesh] OR "Major Depressive Disorder 1" [Supplementary Concept] OR "Major Depressive Disorder 2" [Supplementary Concept])) OR "Renal Insufficiency, Chronic"[Mesh]) OR ("Noncommunicable Diseases"[Mesh] OR "Coronary Artery Disease"[Mesh])) OR ("Chronic Disease"[Mesh] OR "Multiple Chronic Conditions"[Mesh])) OR "Pulmonary Disease, Chronic Obstructive"[Mesh])) OR (type 2 diabetes mellitus [tiab] OR type 1 diabetes mellitus [tiab] OR diabetes mellitus [tiab] OR diabetes [tiab] OR hypertension [tiab] OR cardiovascular disease [tiab] OR coronary heart disease [tiab] OR heart failure [tiab] OR depression [tiab] OR chronic obstructive pulmonary disease [tiab] OR copd [tiab] OR chronic kidney disease [tiab] OR chronic diseases [tiab] OR non communicable diseases[all]))) OR ((((((((((((((((((((("Neglected Diseases") OR "Buruli Ulcer"[Mesh]) OR (ulcer, buruli) OR mycobacterium ulcerans OR "Chagas Disease"[Mesh]) OR "Dengue"[Mesh]) OR "Chikungunya Fever"[Mesh]) OR "Dracunculiasis"[Mesh]) OR ("Echinococcosis"[Mesh] OR "Echinococcosis, Pulmonary"[Mesh] OR "Echinococcosis, Hepatic"[Mesh] OR "Alveolar echinococcosis"[Supplementary Concept])) OR "Trypanosomiasis, African"[Mesh]) OR ("Leishmaniasis"[Mesh] OR "Leishmaniasis, Diffuse Cutaneous"[Mesh] OR "Leishmaniasis, Cutaneous"[Mesh] OR "Leishmaniasis, Visceral"[Mesh] OR "Leishmaniasis, Mucocutaneous"[Mesh])) OR ("Leprosy"[Mesh] OR "Leprosy, Multibacillary"[Mesh] OR "Leprosy, Paucibacillary"[Mesh] OR "Leprosy, Tuberculoid"[Mesh])) OR "Elephantiasis, Filarial"[Mesh]) OR ("Onchocerciasis"[Mesh] OR "Onchocerciasis, Ocular"[Mesh])) OR "Rabies"[Mesh]) OR ("Schistosomiasis"[Mesh] OR "Neuroschistosomiasis"[Mesh])) OR "Taeniasis"[Mesh]) OR "Neurocysticercosis"[Mesh]) OR ("Cysticercosis"[Mesh] OR "Submacular Cysticercosis"[Supplementary Concept])) OR "Trachoma"[Mesh]) OR "Yaws"[Mesh]) OR "Chromoblastomycosis"[Mesh]) OR "Scabies"[Mesh] OR "Snake Bites"[Mesh])) OR (ulcer, buruli [tiab] OR mycobacterium ulcerans [tiab] OR ((disease, chagas [tiab]) OR american trypanosomiasis [tiab]) OR trypanosoma cruzi [tiab] OR (dengue [tiab]) OR mosquito [tiab] OR (chikungunya [tiab]) OR chikungunya virus [tiab] OR (((dracunculiasis [tiab]) OR guinea-worm disease [tiab]) OR dracunculus medinensis [tiab]) OR dracunculiasis [tiab] ((((((((((echinococcosis [tiab]) OR echinococcus granulosus [tiab]) OR cystic echinococcosis [tiab]) OR hydatidosis [tiab]) OR hydatid disease [tiab]) OR alveolar echinococcosis) OR echinococcus multilocularis) OR polycystic echinococcosis) OR echinococcus vogeli) OR unicystic echinococcosis) OR echinococcus oligarthrus) OR echinococcosis [tiab] OR ((((((((((foodborne trematodiases [tiab]) OR trematode worms [tiab]) OR flukes [tiab]) OR freshwater snail [tiab]) OR freshwater fish[tiab]) OR clonorchis sinensis [tiab]) OR opisthorchis viverrini [tiab]) OR opisthorchis felineus [tiab]) OR fasciola hepatica [tiab]) OR fasciola gigantica [tiab]) OR paragonimus [tiab]) OR disease, foodborne [tiab] OR ((((((human african

trypanosomiasis [tiab]) OR sleeping sickness [tiab]) OR trypanosoma brucei gambiense [tiab]) OR tsetse flies [tiab]) OR glossina genus [tiab]) OR trypanosoma brucei rhodesiense [tiab]) OR nagana [tiab]) OR trypanosomiases, african [tiab] OR (((((((leishmaniasis [tiab]) OR visceral leishmaniasis [tiab]) OR cutaneous leishmaniasis [tiab]) OR mucocutaneous leishmaniasis [tiab]) OR kala-azar [tiab]) OR leishmania parasites [tiab]) OR phlebotomine sandflies [tiab]) OR leishmaniases [tiab] OR ((((leprosy [tiab]) OR hansen's disease [tiab]) OR mycobacterium leprae [tiab]) OR leprosy [tiab]) OR hansen disease [tiab] OR ((((((((((lymphatic filariasis [tiab]) OR elephantiasis [tiab]) OR nematodes [tiab]) OR roundworms [tiab]) OR wuchereria bancrofti [tiab]) OR brugia malayi [tiab]) OR brugia timori [tiab]) OR culex [tiab]) OR anopheles [tiab]) OR aedes [tiab]) OR elephantiasis, filarial [tiab] OR (((((onchocerciasis [tiab]) OR river blindness [tiab]) OR onchocerca volvulus [tiab]) OR simulium [tiab]) OR blackflies [tiab]) OR onchocerciasis [tiab] OR (rabies [tiab]) OR lyssa [tiab] OR ((((((((((((schistosomiasis [tiab]) OR parasitic worms [tiab]) OR blood flukes [tiab]) OR trematode worms [tiab]) OR intestinal schistosomiasis [tiab]) OR schistosoma mansoni [tiab]) OR schistosoma japonicum [tiab]) OR schistosoma mekongi [tiab]) OR schistosoma guineensis [tiab]) OR schistosoma intercalatum [tiab]) OR urogenital schistosomiasis [tiab]) OR schistosoma haematobium [tiab]) OR schistosomiasis [tiab]OR ((((((((((soil-transmitted helminthiases [tiab]) OR soil-transmitted helminth infection [tiab]) OR roundworm [tiab]) OR ascaris lumbricoides [tiab]) OR whipworm [tiab]) OR trichuris trichiura [tiab]) OR hookworms [tiab]) OR necator americanus [tiab]) OR ancylostoma duodenale [tiab]) OR helminthiases [tiab]) OR infections, nematomorpha [tiab] OR (((((((((((taeniasis/cysticercosis [tiab]) OR taeniasis [tiab]) OR cysticercosis [tiab]) OR tapeworms [tiab]) OR taenia solium [tiab]) OR pork tapeworm [tiab]) OR taenia saginata [tiab]) OR beef tapeworm [tiab]) OR taenia asiatica [tiab]) OR cysticerci [tiab]) OR cysticercus [tiab]) OR cysticercosis [tiab] OR ((trachoma [tiab]) OR chlamydia trachomatis [tiab]) OR trachoma [tiab] OR ((((endemic treponematoses [tiab]) OR yaws [tiab]) OR treponema pallidum [tiab]) OR pertenue [tiab]) OR frambesia [tiab] OR ((((disease, neglected) OR "neglected tropical disease") OR "tropical disease") OR "neglected disease")))) NOT (fracture OR injury OR malignant neoplasm OR cancer surgery OR cardiovascular surgery OR stent OR surgery OR cancer OR stent OR (animals NOT humans))

4396 291119

- Filters activated: published in the last 10 years, Humans, English, French, Portuguese, Spanish. Clear all to show 6553 items.

Embase

ALL

((((((("Outcome and Process Assessment (Health Care)"[Mesh]) OR ("Process Assessment (Health Care)"[Mesh] OR "Health Services Research"[Mesh]))) OR (Process Assessments (Health Care) OR Process Assessment [tiab] OR Process Assessments [tiab] OR Process Measures [tiab] OR Process Measure [tiab] OR Outcome and Process Assessment [tiab] OR Process evaluation OR fidelity [tiab] OR implementation process))) AND ((((randomized controlled trial[pt] OR controlled clinical trial[pt] OR randomized[tiab] OR placebo[tiab] OR drug therapy[sh] OR randomly[tiab] OR trial[tiab] OR groups[tiab] OR randomised controlled study [tiab] OR randomised controlled trial [tiab] OR rct [tiab] OR non-rct [tiab] OR comparative studies [tiab] OR control group study [tiab] OR control group trial [tiab] OR controlled trial [tiab] OR experimental studies [tiab] OR feasibility studies [tiab] OR field studies [tiab] OR non

experimental studies [tiab] OR non experimental study [tiab] OR nonexperimental studies [tiab] OR nonexperimental study [tiab] OR observation studies [tiab] OR observation study [tiab] OR observational studies [tiab] OR prevention trial [tiab] OR preventive study [tiab] OR preventive trial [tiab] OR quality improvement studies [tiab] OR quasiexperimental study [tiab] OR validation studies [tiab] OR mixed method study [tiab])) OR ((("Clinical Trial" [Publication Type] OR "Clinical Trials as Topic"[Mesh] OR "Controlled Clinical Trial" [Publication Type] OR "Pragmatic Clinical Trial" [Publication Type] OR "Clinical Trial, Phase IV" [Publication Type] OR "Clinical Trial, Phase III" [Publication Type] OR "Clinical Trial, Phase II" [Publication Type] OR "Adaptive Clinical Trial" [Publication Type] OR "Non-Randomized Controlled Trials as Topic"[Mesh] OR "Adaptive Clinical Trials as Topic"[Mesh] OR "Pragmatic Clinical Trials as Topic"[Mesh] OR "Evaluation Studies" [Publication Type] OR "Randomized Controlled Trial" [Publication Type] OR "Validation Studies" [Publication Type]) OR ("Observational Studies as Topic"[Mesh] OR "Observational Study" [Publication Type])) OR ("Clinical Study" [Publication Type] OR "Clinical Studies as Topic"[Mesh]) OR randomized controlled trial[pt] OR controlled clinical trial[pt] OR randomized[tiab] OR placebo[tiab] OR drug therapy[sh] OR randomly[tiab] OR trial[tiab] OR groups[tiab] OR (randomized controlled trial[pt] OR controlled clinical trial[pt] OR randomized controlled trials[mh] OR random allocation[mh] OR double-blind method[mh] OR single-blind method[mh] OR clinical trial[pt] OR clinical trials[mh] OR "clinical trial"[tw] OR comparative study[pt] OR evaluation studies[pt] OR follow-up studies[mh] OR prospective studies[mh] OR cross-over studies[mh] OR control[tw]) NOT (animals[mh] NOT humans[mh]))))) AND
(((
((((((((((((((((((((((((((("Developing Countries"[Mesh]) OR "Afghanistan"[Mesh]) OR "Benin"[Mesh]) OR "Burkina Faso"[Mesh])) OR "Burundi"[Mesh]) OR "Central African Republic"[Mesh]) OR "Chad"[Mesh]) OR "Democratic Republic of the Congo"[Mesh]) OR "Ethiopia"[Mesh]) OR "Gambia"[Mesh]) OR "Guinea"[Mesh]) OR "Haiti"[Mesh]) OR "Democratic People's Republic of Korea"[Mesh]) OR "Liberia"[Mesh]) OR "Madagascar"[Mesh]) OR "Malawi"[Mesh]) OR "Nepal"[Mesh]) OR "Niger"[Mesh]) OR "Rwanda"[Mesh]) OR "Sierra Leone"[Mesh]) OR "Somalia"[Mesh]) OR "South Sudan"[Mesh]) OR "Syria"[Mesh]) OR "Tajikistan"[Mesh]) OR "Tanzania"[Mesh]) OR "Togo"[Mesh]) OR "Uganda"[Mesh]) OR "Yemen"[Mesh]) OR "Mali"[Mesh]) OR "Angola"[Mesh]) OR "Bangladesh"[Mesh]) OR "Bhutan"[Mesh]) OR "Bolivia"[Mesh]) OR "Cabo Verde"[Mesh]) OR "Cambodia"[Mesh]) OR "Cameroon"[Mesh]) OR "Comoros"[Mesh]) OR "Cote d'Ivoire"[Mesh]) OR "Djibouti"[Mesh]) OR "Egypt"[Mesh]) OR "El Salvador"[Mesh]) OR "Ghana"[Mesh]) OR "Honduras"[Mesh]) OR "India"[Mesh]) OR "Papua New Guinea"[Mesh]) OR "Indonesia"[Mesh]) OR "Kenya"[Mesh]) OR "Micronesia"[Mesh]) OR "Kyrgyzstan"[Mesh]) OR "Laos"[Mesh]) OR "Lesotho"[Mesh]) OR "Mauritania"[Mesh]) OR "Moldova"[Mesh]) OR "Mongolia"[Mesh]) OR "Morocco"[Mesh]) OR "Myanmar"[Mesh]) OR "Nicaragua"[Mesh]) OR "Nigeria"[Mesh]) OR "Pakistan"[Mesh]) OR "Philippines"[Mesh]) OR "Sao Tome and Principe"[Mesh]) OR "Senegal"[Mesh]) OR "Melanesia"[Mesh]) OR "Sudan"[Mesh]) OR "Swaziland"[Mesh]) OR "Timor-Leste"[Mesh]) OR "Tunisia"[Mesh]) OR "Ukraine"[Mesh]) OR "Uzbekistan"[Mesh]) OR "Vanuatu"[Mesh]) OR "Vietnam"[Mesh]) OR "Zambia"[Mesh]) OR "Zimbabwe"[Mesh]) OR "Middle East"[Mesh]) OR "Albania"[Mesh]) OR "Algeria"[Mesh]) OR "American Samoa"[Mesh]) OR "Argentina"[Mesh]) OR "Armenia"[Mesh]) OR "Azerbaijan"[Mesh]) OR "Republic of Belarus"[Mesh]) OR

"Belize"[Mesh]) OR "Bosnia and Herzegovina"[Mesh]) OR "Botswana"[Mesh]) OR "Brazil"[Mesh]) OR "Bulgaria"[Mesh]) OR "China"[Mesh]) OR "Colombia"[Mesh]) OR "Costa Rica"[Mesh]) OR "Cuba"[Mesh]) OR "Dominica"[Mesh]) OR "Dominican Republic"[Mesh]) OR "Equatorial Guinea"[Mesh]) OR "Ecuador"[Mesh]) OR "Fiji"[Mesh]) OR "Gabon"[Mesh]) OR "Georgia (Republic)"[Mesh]) OR "Grenada"[Mesh]) OR "Guatemala"[Mesh]) OR "Guyana"[Mesh]) OR "Iran"[Mesh]) OR "Iraq"[Mesh]) OR "Jamaica"[Mesh]) OR "Jordan"[Mesh]) OR "Kazakhstan"[Mesh]) OR "Kosovo"[Mesh]) OR "Lebanon"[Mesh]) OR "Libya"[Mesh]) OR "Macedonia (Republic)"[Mesh]) OR "Malaysia"[Mesh]) OR "Indian Ocean Islands"[Mesh]) OR "Mauritius"[Mesh]) OR "Mexico"[Mesh]) OR "Montenegro"[Mesh]) OR "Namibia"[Mesh]) OR "Paraguay"[Mesh]) OR "Peru"[Mesh]) OR "Romania"[Mesh]) OR "Russia"[Mesh]) OR "Samoa"[Mesh]) OR "Serbia"[Mesh]) OR "Sri Lanka"[Mesh]) OR "South Africa"[Mesh]) OR "Saint Lucia"[Mesh]) OR "Saint Vincent and the Grenadines"[Mesh]) OR "Suriname"[Mesh]) OR "Thailand"[Mesh]) OR "Tonga"[Mesh]) OR "Turkey"[Mesh]) OR "Turkmenistan"[Mesh]) OR "Venezuela"[Mesh]))) AND ((((((((((("Diabetes Mellitus"[Mesh] OR "Diabetes Mellitus, Type 2"[Mesh] OR "Diabetes Mellitus, Type 1"[Mesh] OR "Diabetes Complications"[Mesh] OR "Diabetes Mellitus, Insulin-Resistant, with Acanthosis Nigricans" [Supplementary Concept] OR "Diabetes, Gestational"[Mesh] OR "Diabetes Mellitus, Insulin-Dependent, 15" [Supplementary Concept] OR "Diabetes Mellitus, Insulin-Dependent, 6" [Supplementary Concept] OR "Diabetes Mellitus, Insulin-Dependent, 10" [Supplementary Concept] OR "Diabetes Mellitus, Insulin-Dependent, 17" [Supplementary Concept] OR "Diabetes Mellitus, Noninsulin-Dependent, 3" [Supplementary Concept] AND "Diabetes Mellitus, Insulin-Dependent, 18" [Supplementary Concept] OR "Diabetes Mellitus, Insulin-Dependent, 2" [Supplementary Concept] AND "Diabetes Mellitus, Insulin-Dependent, 3" [Supplementary Concept]) AND ("Hypertension"[Mesh] OR "Essential Hypertension"[Mesh] OR "Hypertension, Malignant"[Mesh])) OR "Cardiovascular Diseases"[Mesh]) OR ("Depression" [Majr] OR "Depressive Disorder"[Mesh] OR "Depressive Disorder, Major"[Mesh] OR "Major Depressive Disorder 1" [Supplementary Concept] OR "Major Depressive Disorder 2" [Supplementary Concept])) OR "Renal Insufficiency, Chronic"[Mesh]) OR ("Noncommunicable Diseases"[Mesh] OR "Coronary Artery Disease"[Mesh])) OR ("Chronic Disease"[Mesh] OR "Multiple Chronic Conditions"[Mesh])) OR "Pulmonary Disease, Chronic Obstructive"[Mesh])) OR (type 2 diabetes mellitus [tiab] OR type 1 diabetes mellitus [tiab] OR diabetes mellitus [tiab] OR diabetes [tiab] OR hypertension [tiab] OR cardiovascular disease [tiab] OR coronary heart disease [tiab] OR heart failure [tiab] OR depression [tiab] OR chronic obstructive pulmonary disease [tiab] OR copd [tiab] OR chronic kidney disease [tiab] OR chronic diseases [tiab] OR non communicable diseases[all]))) OR (((((((((((((((((((((("Neglected Diseases") OR "Buruli Ulcer"[Mesh]) OR (ulcer, buruli) OR mycobacterium ulcerans OR "Chagas Disease"[Mesh]) OR "Dengue"[Mesh]) OR "Chikungunya Fever"[Mesh]) OR "Dracunculiasis"[Mesh]) OR ("Echinococcosis"[Mesh] OR "Echinococcosis, Pulmonary"[Mesh] OR "Echinococcosis, Hepatic"[Mesh] OR "Alveolar echinococcosis"[Supplementary Concept])) OR "Trypanosomiasis, African"[Mesh]) OR ("Leishmaniasis"[Mesh] OR "Leishmaniasis, Diffuse Cutaneous"[Mesh] OR "Leishmaniasis, Cutaneous"[Mesh] OR "Leishmaniasis, Visceral"[Mesh] OR "Leishmaniasis, Mucocutaneous"[Mesh])) OR ("Leprosy"[Mesh] OR "Leprosy, Multibacillary"[Mesh] OR "Leprosy, Paucibacillary"[Mesh] OR "Leprosy, Tuberculoid"[Mesh])) OR "Elephantiasis, Filarial"[Mesh]) OR ("Onchocerciasis"[Mesh] OR "Onchocerciasis, Ocular"[Mesh]))

OR "Rabies"[Mesh]) OR ("Schistosomiasis"[Mesh] OR "Neuroschistosomiasis"[Mesh])) OR "Taeniasis"[Mesh]) OR "Neurocysticercosis"[Mesh]) OR ("Cysticercosis"[Mesh] OR "Submacular Cysticercosis"[Supplementary Concept])) OR "Trachoma"[Mesh]) OR "Yaws"[Mesh]) OR "Chromoblastomycosis"[Mesh]) OR "Scabies"[Mesh] OR "Snake Bites"[Mesh])) OR (ulcer, buruli [tiab] OR mycobacterium ulcerans [tiab] OR ((disease, chagas [tiab]) OR american trypanosomiasis [tiab]) OR trypanosoma cruzi [tiab] OR (dengue [tiab]) OR mosquito [tiab] OR (chikungunya [tiab]) OR chikungunya virus [tiab] OR (((dracunculiasis [tiab]) OR guinea-worm disease [tiab]) OR dracunculus medinensis [tiab]) OR dracunculiasis [tiab] ((((((((((echinococcosis [tiab]) OR echinococcus granulosus [tiab]) OR cystic echinococcosis [tiab]) OR hydatidosis [tiab]) OR hydatid disease [tiab]) OR alveolar echinococcosis) OR echinococcus multilocularis) OR polycystic echinococcosis) OR echinococcus vogeli) OR unicystic echinococcosis) OR echinococcus oligarthrus) OR echinococcosis [tiab] OR (((((((((((foodborne trematodiases [tiab]) OR trematode worms [tiab]) OR flukes [tiab]) OR freshwater snail [tiab]) OR freshwater fish[tiab]) OR clonorchis sinensis [tiab]) OR opisthorchis viverrini [tiab]) OR opisthorchis felineus [tiab]) OR fasciola hepatica [tiab]) OR fasciola gigantica [tiab]) OR paragonimus [tiab]) OR disease, foodborne [tiab] OR (((((((human african trypanosomiasis [tiab]) OR sleeping sickness [tiab]) OR trypanosoma brucei gambiense [tiab]) OR tsetse flies [tiab]) OR glossina genus [tiab]) OR trypanosoma brucei rhodesiense [tiab]) OR nagana [tiab]) OR trypanosomiases, african [tiab] OR (((((((leishmaniasis [tiab]) OR visceral leishmaniasis [tiab]) OR cutaneous leishmaniasis [tiab]) OR mucocutaneous leishmaniasis [tiab]) OR kala-azar [tiab]) OR leishmania parasites [tiab]) OR phlebotomine sandflies [tiab]) OR leishmaniases [tiab] OR ((((leprosy [tiab]) OR hansen's disease [tiab]) OR mycobacterium leprae [tiab]) OR leprosy [tiab]) OR hansen disease [tiab] OR ((((((((((lymphatic filariasis [tiab]) OR elephantiasis [tiab]) OR nematodes [tiab]) OR roundworms [tiab]) OR wuchereria bancrofti [tiab]) OR brugia malayi [tiab]) OR brugia timori [tiab]) OR culex [tiab]) OR anopheles [tiab]) OR aedes [tiab]) OR elephantiasis, filarial [tiab] OR (((((onchocerciasis [tiab]) OR river blindness [tiab]) OR onchocerca volvulus [tiab]) OR simulium [tiab]) OR blackflies [tiab]) OR onchocerciasis [tiab] OR (rabies [tiab]) OR lyssa [tiab] OR ((((((((((((schistosomiasis [tiab]) OR parasitic worms [tiab]) OR blood flukes [tiab]) OR trematode worms [tiab]) OR intestinal schistosomiasis [tiab]) OR schistosoma mansoni [tiab]) OR schistosoma japonicum [tiab]) OR schistosoma mekongi [tiab]) OR schistosoma guineensis [tiab]) OR schistosoma intercalatum [tiab]) OR urogenital schistosomiasis [tiab]) OR schistosoma haematobium [tiab]) OR schistosomiasis [tiab]OR ((((((((((soil-transmitted helminthiases [tiab]) OR soil-transmitted helminth infection [tiab]) OR roundworm [tiab]) OR ascaris lumbricoides [tiab]) OR whipworm [tiab]) OR trichuris trichiura [tiab]) OR hookworms [tiab]) OR necator americanus [tiab]) OR ancylostoma duodenale [tiab]) OR helminthiases [tiab]) OR infections, nematomorpha [tiab] OR (((((((((((taeniasis/cysticercosis [tiab]) OR taeniasis [tiab]) OR cysticercosis [tiab]) OR tapeworms [tiab]) OR taenia solium [tiab]) OR pork tapeworm [tiab]) OR taenia saginata [tiab]) OR beef tapeworm [tiab]) OR taenia asiatica [tiab]) OR cysticerci [tiab]) OR cysticercus [tiab]) OR cysticercosis [tiab] OR ((trachoma [tiab]) OR chlamydia trachomatis [tiab]) OR trachoma [tiab] OR ((((endemic treponematoses [tiab]) OR yaws [tiab]) OR treponema pallidum [tiab]) OR pertenue [tiab]) OR frambesia [tiab] OR ((((disease, neglected) OR "neglected tropical disease") OR "tropical disease") OR "neglected disease")))) NOT (fracture OR injury OR malignant neoplasm OR cancer surgery OR cardiovascular surgery OR stent OR surgery OR cancer OR stent OR (animals NOT humans))

4396 291119

- Filters activated: published in the last 10 years, Humans, English, French, Portuguese, Spanish. Clear all to show 6553 items.

Web of Science

TS=(("health care quality" OR "program evaluation" OR "outcome assessment" OR "treatment outcome" OR "clinical governance" OR (("health care" OR "healthcare") NEAR/1 (quality OR "quality assurance" OR "quality indicator" OR access OR evaluation)) OR "process assessment" OR "quality of care research" OR "quality of health care" OR "outcome management" OR "patient outcome" OR "standard of care" OR "causal mechanisms" OR "process evaluation") AND ("non communicable disease" OR "non-communicable disease" OR "non-communicable diseases" OR "noncommunicable disease" OR "noncommunicable diseases" OR "neglected tropical diseases" OR "non insulin dependent diabetes mellitus" OR "insulin dependent diabetes mellitus" OR "cardiovascular disease" OR "depression"/mj OR "chronic obstructive lung disease" OR "chronic kidney failure" OR "buruli ulcer" OR "chagas disease" OR "dengue" OR "chikungunya" OR "dracunculiasis" OR "echinococcosis" OR "african trypanosomiasis" OR "leishmaniasis" OR "leprosy" OR "lymphatic filariasis" OR "onchocerciasis" OR "rabies" OR "schistosomiasis" OR "taeniasis" OR "trachoma" OR "yaws" OR "chromomycosis" OR "mycosis" OR "scabies" OR "ectoparasite" OR "snakebite" OR "type 2 diabetes mellitus" OR "type 1 diabetes mellitus" OR "cardiovascular disease" OR "chronic obstructive pulmonary disease" OR "chronic kidney disease" OR "buruli ulcer" OR "chagas disease" OR "dengue" OR "chikungunya" OR "draculiasis-guinea worm diseases" OR "guinea worm disease" OR "dracunculosis dracunculiasis" OR "echinococcosis" OR "foodborne trematodiases" OR "human african trypanosomiasis" OR "sleeping sickness" OR "african trypanosomiasis" OR "african human trypanosomiasis" OR leishmaniasis OR "leprosy-hansens disease" OR "lymphatic filariasis" OR "onchocerciasis-river blindness" OR "onchocercosis" OR rabies OR schistosomiasis OR "soil-transmitted helminthiases" OR taeniasis OR cysticercosis OR trachoma OR "yaws-endemic treponematoses" OR chromoblastomycosis OR "deep mycoses" OR scabies OR ectoparasites OR "snakebite envenoming") AND ((((developing OR "least developed" OR "less developed" OR "under-developed" OR "third world" OR "low income" OR "middle income" OR "lower middle income" OR "upper middle income") NEAR/1 (countr* OR nation OR nations))) OR "afghanistan" OR "benin" OR "burkina faso" OR "burundi" OR "central african republic" OR "chad" OR "democratic republic congo" OR "eritrea" OR "ethiopia" OR "gambia" OR "guinea" OR "guinea-bissau" OR "haiti" OR "korea" OR "liberia" OR "madagascar" OR "malawi" OR "mozambique" OR "nepal" OR "niger" OR "rwanda" OR "sierra leone" OR "somalia" OR "south sudan" OR "syrian arab republic" OR "tajikistan" OR "tanzania" OR "togo" OR "uganda" OR "yemen" OR "mali" OR afghanistan OR benin OR "burkina faso" OR burundi OR "central african republic" OR eritrea OR ethiopia OR gambia OR guinea OR "guinea bissau" OR haiti OR korea OR liberia OR madagascar OR malawi OR mali OR mozambique OR nepal OR niger OR rwanda OR "sierra leone" OR somalia OR "south sudan" OR "syrian arab republic" OR "syria" OR tajikistan OR tanzania OR togo OR uganda OR yemen OR "angola" OR "bangladesh" OR "bhutan" OR "bolivia" OR "cape verde" OR "cambodia" OR "cameroon" OR "comoros" OR "congo" OR "cote d`ivoire" OR "djibouti" OR "egypt" OR "el salvador" OR "ghana" OR "honduras" OR "india" OR "papua new guinea" OR "indonesia" OR "kenya" OR "kiribati" OR "kyrgyzstan" OR "laos" OR "lesotho" OR "mauritania" OR "federated states of micronesia" OR "moldova" OR "mongolia" OR "morocco" OR "myanmar" OR "nicaragua" OR "nigeria" OR "pakistan" OR "philippines" OR "sao tome and principe" OR "senegal" OR "solomon islands" OR "sudan" OR "swaziland" OR "timor-leste" OR "tunisia" OR "ukraine" OR "uzbekistan" OR "vanuatu" OR "viet nam" OR "zambia" OR "zimbabwe" OR "palestine" OR angola OR bangladesh OR bhutan OR bolivia OR "cabo verde" OR cambodia OR cameroon OR comoros OR congo OR "cote d ivoire" OR djibouti OR egypt OR "el salvador" OR ghana OR honduras OR india OR indonesia OR kenya OR kiribati OR "kyrgyz republic" OR kyrgyzstan OR "lao pdr" OR lesotho OR mauritania OR "federated states of micronesia" OR moldova OR mongolia OR morocco OR myanmar OR nicaragua OR nigeria OR pakistan OR "papua new guinea" OR philippines OR "sao tome and principe" OR senegal OR "solomon islands" OR sudan OR swaziland OR "timor-leste" OR tunisia

OR ukraine OR uzbekistan OR vanuatu OR vietnam OR "west bank and gaza" OR zambia OR zimbabwe OR palestine OR "albania" OR "algeria" OR "american samoa" OR "argentina" OR "armenia" OR "azerbaijan" OR "belarus" OR "belize" OR "bosnia and herzegovina" OR "botswana" OR "brazil" OR "bulgaria" OR "china" OR "colombia" OR "costa rica" OR "cuba" OR "dominica" OR "dominican republic" OR "equatorial guinea" OR "ecuador" OR "fiji" OR "gabon" OR "georgia (republic)" OR "grenada" OR "guatemala" OR "guyana" OR "iran" OR "iraq" OR "jamaica" OR "jordan" OR "kazakhstan" OR "kosovo" OR "lebanon" OR "libyan arab jamahiriya" OR "macedonia (republic)" OR "malaysia" OR "maldives" OR "marshall islands" OR "mauritius" OR "mexico" OR "montenegro (republic)" OR "namibia" OR "nauru" OR "paraguay" OR "peru" OR "romania" OR "russian federation" OR "samoan islands" OR "serbia" OR "sri lanka" OR "south africa" OR "saint lucia" OR "saint vincent and the grenadines" OR "suriname" OR "thailand" OR "tonga" OR "turkey (republic)" OR "turkmenistan" OR "tuvalu" OR "venezuela" OR albania OR algeria OR "american samoa" OR argentina OR armenia OR azerbaijan OR belarus OR belize OR "bosnia and herzegovina" OR botswana OR brazil OR bulgaria OR china OR colombia OR "costa rica" OR cuba OR dominica OR "dominican republic" OR "equatorial guinea" OR ecuador OR fiji OR gabon OR georgia OR grenada OR guatemala OR guyana OR "iran islamic rep." OR iran OR iraq OR jamaica OR jordan OR kazakhstan OR kosovo OR lebanon OR libya OR "macedonia fyr" OR malaysia OR maldives OR "marshall islands" OR mauritius OR mexico OR montenegro OR namibia OR nauru OR paraguay OR peru OR romania OR "russian federation" OR russia OR samoa OR serbia OR "sri lanka" OR "south africa" OR "st. lucia" OR "st. vincent and the grenadines" OR suriname OR thailand OR tonga OR turkey OR turkmenistan OR tuvalu OR venezuela) AND ("controlled clinical trial (topic)" OR "crossover procedure" OR "double blind procedure" OR "randomized controlled trial" OR "single blind procedure" OR "comparative study" OR "controlled study" OR "experimental study" OR "feasibility study" OR "field study" OR "observational study" OR "panel study" OR "prevention study" OR "quality improvement study" OR "quasi experimental study" OR "replication study" OR "trend study" OR "twin study" OR "validation study" OR "randomised controlled study" OR "randomised controlled trial" OR "rct" OR "non-rct" OR "comparative studies" OR "control group study" OR "control group trial" OR "controlled trial" OR "experimental studies" OR "feasibility studies" OR "field studies" OR "non experimental studies" OR "non experimental study" OR "nonexperimental studies" OR "nonexperimental study" OR "observation studies" OR "observation study" OR "observational studies" OR "panel study" OR "prevention trial" OR "preventive study" OR "preventive trial" OR "quality improvement studies" OR "quasiexperimental study" OR "replication studies" OR "trend studies" OR "twin studies" OR "twins study" OR "validation studies" OR random* OR factorial* OR crossover* OR ((cross NEAR/1 over*)) OR placebo* OR (doubl* AND blind*) OR (singl* AND blind*) OR assign* OR volunteer*) NOT ("fracture" OR "injury" OR "malignant neoplasm" OR "cancer surgery" OR "cardiovascular surgery" OR "stent" OR "surgery" OR cancer OR stent OR "cardiovascular surgery" OR surgery) NOT ((animal* OR plant* OR rats OR mice OR pigs) NOT human*))

91 271119

75 090120
Restricción 2009 - 2020

<u>Ebsco Host</u>

Business Source Premier, ERIC, Green FILE and PSYNDEX

Published Date: 20080101-20191131

Process Evaluation – All Text

health care quality OR program evaluation OR outcome assessment OR treatment outcome OR clinical governance OR health care OR healthcare OR quality assurance OR quality indicator OR access OR evaluation OR process assessment OR quality of care research OR quality of health care OR outcome management OR patient outcome OR standard of care OR causal mechanisms OR process evaluation OR fidelity

NTDs and NCDs - All Text

(buruli ulcer OR mycobacterium ulcerans OR chagas disease OR american trypanosomiasis OR trypanosoma cruzi OR dengue OR mosquito OR chikungunya OR dracunculiasis OR guinea worm disease OR dracunculus medinensis OR echinococcosis OR echinococcus granulosus OR cystic echinococcosis OR hydatidosis OR hydatid disease OR alveolar echinococcosis OR echinococcus multilocularis OR polycystic echinococcosis OR echinococcus vogeli OR unicystic echinococcosis OR echinococcus oligarthrus OR foodborne trematodiases OR trematode worms OR flukes OR freshwater snails OR freshwater fish OR clonorchis sinensis OR opisthorchis viverrini OR opisthorchis Felineus OR fasciola hepatica OR fasciola gigantica OR paragonimus OR human african trypanosomiasis OR sleeping sickness OR trypanosoma brucei gambiense OR tsetse flies OR glossina genus OR trypanosoma brucei rhodesiense OR nagana OR leishmaniasis OR visceral leishmaniasis OR cutaneous leishmaniasis OR mucocutaneous leishmaniasis OR kala-azar OR phlebotomine sandflies OR leprosy OR hansen's disease OR mycobacterium leprae OR lymphatic filariasis OR elephantiasis OR nematodes OR roundworms OR (roundworms or nematodes) OR wuchereria bancrofti OR brugia malayi OR brugia timori OR culex OR anopheles OR aedes OR onchocerciasis OR river blindness OR onchocerca volvulus OR simulium OR blackflies OR rabies OR schistosomiasis OR parasitic worms OR blood fluke OR trematode worms OR Intestinal schistosomiasis OR schistosoma mansoni OR schistosoma japonicum OR schistosoma mekongi OR schistosoma guineensis OR schistosoma intercalatum OR urogenital schistosomiasis OR schistosoma haematobium OR soil-transmitted helminthiases OR soil-transmitted helminth infection OR roundworm OR ascaris lumbricoides OR whipworm OR trichuris trichiura OR hookworms in humans OR hookworms OR necator americanus OR ancylostoma duodenale OR taeniasis/cysticercosis OR taeniasis OR cysticercosis OR tapeworm OR taenia solium OR pork tapeworm OR taenia saginata OR beef tapeworm OR taenia asiatica OR cysticerci OR trachoma OR chlamydia trachomatis OR endemic treponematoses OR yaws OR treponema pallidum OR pertenue OR neglected tropical diseases OR tropical diseases OR neglected diseases OR ntds OR yaws OR chromomycosis OR deep mycosis OR scabies OR ectoparasite OR snakebite OR yaws-endemic treponematoses OR chromoblastomycosis OR deep mycoses OR ectoparasites OR snakebite envenoming) OR (non communicable disease OR non-communicable disease OR non-communicable diseases OR noncommunicable disease OR noncommunicable diseases OR non insulin dependent diabetes mellitus OR insulin dependent diabetes mellitus OR cardiovascular disease OR depression OR chronic obstructive lung disease OR chronic kidney failure OR OR type 2 diabetes mellitus OR type 1 diabetes mellitus OR coronary heart disease OR chronic obstructive pulmonary disease OR chronic kidney disease)

LMIC - All Text

third world OR low income country OR middle income country OR lower middle income country OR upper middle income country OR afghanistan OR benin OR burkina faso OR burundi OR central african republic OR chad OR democratic republic congo OR eritrea OR ethiopia OR gambia OR guinea OR guinea-bissau OR haiti OR korea OR liberia OR madagascar OR malawi OR mozambique OR nepal OR niger OR rwanda OR sierra leone OR somalia OR south sudan OR syrian arab republic OR tajikistan OR tanzania OR togo OR uganda OR yemen OR mali OR angola OR bangladesh OR bhutan OR bolivia OR cape verde OR cambodia OR cameroon OR comoros OR congo OR cote d`ivoire OR djibouti OR egypt OR el salvador OR ghana OR honduras OR india OR papua new guinea OR indonesia OR kenya OR kiribati OR kyrgyzstan OR laos OR lesotho OR mauritania OR federated states of micronesia OR moldova OR mongolia OR morocco OR myanmar OR nicaragua OR nigeria OR pakistan OR philippines OR sao tome and principe OR senegal OR solomon islands OR sudan OR swaziland OR timor-leste OR tunisia OR ukraine OR uzbekistan OR vanuatu OR vietnam OR zambia OR zimbabwe OR palestine OR albania OR algeria OR american samoa OR argentina OR armenia OR azerbaijan OR belarus OR belize OR bosnia and herzegovina OR botswana OR brazil OR bulgaria OR china OR colombia OR costa rica OR cuba OR dominica OR dominican republic OR equatorial guinea OR ecuador OR fiji OR gabon OR georgia (republic) OR grenada OR guatemala OR guyana OR iran OR iraq OR jamaica OR jordan OR kazakhstan OR kosovo OR lebanon OR libyan arab jamahiriya OR macedonia (republic) OR malaysia OR maldives OR marshall islands OR mauritius OR mexico OR montenegro (republic) OR namibia OR nauru OR paraguay OR peru OR romania OR russian federation OR samoan islands OR serbia OR sri lanka OR south africa OR saint lucia OR saint vincent and the grenadines OR suriname OR thailand OR tonga OR turkey (republic) OR turkmenistan OR tuvalu OR venezuela

Type of studies – AB Abstract

controlled clinical trial OR crossover procedure OR double blind procedure OR randomized controlled trial OR single blind procedure OR comparative study OR controlled study OR experimental study OR feasibility study OR field study OR observational study OR panel study OR prevention study OR quality improvement study OR quasi experimental study OR replication study OR trend study OR twin study OR validation study OR randomised controlled study OR randomised controlled trial OR rct OR non-rct OR comparative studies OR control group study OR control group trial OR controlled trial OR experimental studies OR feasibility studies OR field studies OR non experimental studies OR non experimental study OR nonexperimental studies OR nonexperimental study OR observation studies OR observation study OR observational studies OR panel study OR prevention trial OR preventive study OR preventive trial OR quality improvement studies OR quasiexperimental study OR replication studies OR trend studies OR twin studies OR twins study OR validation studies

449 281119

124 090120 2009 - 2020

Global Index Medicus

Process Evaluation

health care quality OR program evaluation OR outcome assessment OR treatment outcome OR clinical governance OR health care OR healthcare OR quality assurance OR quality indicator OR access OR evaluation OR process assessment OR quality of care research OR quality of health care OR outcome management OR patient outcome OR standard of care OR causal mechanisms OR process evaluation OR fidelity OR program evaluation OR outcome assessment OR treatment outcome OR clinical governance OR health care OR healthcare OR quality assurance OR quality indicator OR access OR evaluation OR process assessment OR quality of care research OR quality of health care OR outcome management OR patient outcome OR standard of care OR causal mechanisms OR process evaluation OR fidelity

NTDs

(tw:(buruli ulcer OR mycobacterium ulcerans OR chagas disease OR american trypanosomiasis OR trypanosoma cruzi OR dengue OR mosquito OR chikungunya OR dracunculiasis OR guinea worm disease OR dracunculus medinensis OR echinococcosis OR echinococcus granulosus OR cystic echinococcosis OR hydatidosis OR hydatid disease OR alveolar echinococcosis OR echinococcus multilocularis OR polycystic echinococcosis OR echinococcus vogeli OR unicystic echinococcosis OR echinococcus oligarthrus OR foodborne trematodiases OR trematode worms OR flukes OR freshwater snails OR freshwater fish OR clonorchis sinensis OR opisthorchis viverrini OR opisthorchis Felineus OR fasciola hepatica OR fasciola gigantica OR paragonimus OR human african trypanosomiasis OR sleeping sickness OR trypanosoma brucei gambiense OR tsetse flies OR glossina genus OR trypanosoma brucei rhodesiense OR nagana OR leishmaniasis OR visceral leishmaniasis OR cutaneous leishmaniasis OR mucocutaneous leishmaniasis OR kala-azar OR phlebotomine sandflies OR leprosy OR hansen's disease OR mycobacterium leprae OR lymphatic filariasis OR elephantiasis OR nematodes OR roundworms)) OR (tw:(roundworms or nematodes)) OR (tw:(wuchereria bancrofti OR brugia malayi OR brugia timori OR culex OR anopheles OR aedes OR onchocerciasis OR river blindness OR onchocerca volvulus OR simulium OR blackflies OR rabies OR schistosomiasis OR parasitic worms OR blood fluke OR trematode worms OR Intestinal schistosomiasis OR schistosoma mansoni OR schistosoma japonicum OR schistosoma mekongi OR schistosoma guineensis OR schistosoma intercalatum OR urogenital schistosomiasis OR schistosoma haematobium OR soil-transmitted helminthiases OR soil-transmitted helminth infection OR roundworm OR ascaris lumbricoides OR whipworm OR trichuris trichiura OR hookworms in humans OR hookworms OR necator americanus OR ancylostoma duodenale OR taeniasis/cysticercosis OR taeniasis OR cysticercosis OR tapeworm OR taenia solium OR pork tapeworm OR taenia saginata OR beef tapeworm OR taenia asiatica OR cysticerci OR trachoma OR chlamydia trachomatis OR endemic treponematoses OR yaws OR treponema pallidum OR pertenue)) OR (tw:(neglected tropical diseases OR tropical diseases OR neglected diseases OR ntds OR yaws OR chromomycosis OR deep mycosis OR scabies OR ectoparasite OR snakebite OR yaws-endemic treponematoses OR chromoblastomycosis OR deep mycoses OR ectoparasites OR snakebite envenoming))

NCDs

(tw:((tw:((tw:((tw:(non communicable disease)) OR (tw:(non-communicable disease)) OR (tw:(non-communicable diseases)) OR (tw:(noncommunicable disease)) OR (tw:(noncommunicable diseases))))))) OR (tw:(non insulin dependent diabetes mellitus

)) OR (tw:(insulin dependent diabetes mellitus)) OR (tw:(cardiovascular disease)) OR (tw:(chronic obstructive lung disease)) OR (tw:(chronic kidney failure)) OR (tw:(type 2 diabetes mellitus)) OR (tw:(type 1 diabetes mellitus)) OR (tw:(coronary heart disease)) OR (tw:(chronic obstructive pulmonary disease)) OR (tw:(chronic kidney disease))

NTDs + NCDs

(tw:((tw:((tw:((tw:((tw:(non communicable disease)) OR (tw:(non-communicable disease)) OR (tw:(non-communicable diseases)) OR (tw:(noncommunicable disease)) OR (tw:(noncommunicable diseases))))))) OR (tw:(non insulin dependent diabetes mellitus)) OR (tw:(insulin dependent diabetes mellitus)) OR (tw:(cardiovascular disease)) OR (tw:(chronic obstructive lung disease)) OR (tw:(chronic kidney failure)) OR (tw:(type 2 diabetes mellitus)) OR (tw:(type 1 diabetes mellitus)) OR (tw:(coronary heart disease)) OR (tw:(chronic obstructive pulmonary disease)) OR (tw:(chronic kidney disease)))) OR (tw:((tw:(buruli ulcer OR mycobacterium ulcerans OR chagas disease OR american trypanosomiasis OR trypanosoma cruzi OR dengue OR mosquito OR chikungunya OR dracunculiasis OR guinea worm disease OR dracunculus medinensis OR echinococcosis OR echinococcus granulosus OR cystic echinococcosis OR hydatidosis OR hydatid disease OR alveolar echinococcosis OR echinococcus multilocularis OR polycystic echinococcosis OR echinococcus vogeli OR unicystic echinococcosis OR echinococcus oligarthrus OR foodborne trematodiases OR trematode worms OR flukes OR freshwater snails OR freshwater fish OR clonorchis sinensis OR opisthorchis viverrini OR opisthorchis Felineus OR fasciola hepatica OR fasciola gigantica OR paragonimus OR human african trypanosomiasis OR sleeping sickness OR trypanosoma brucei gambiense OR tsetse flies OR glossina genus OR trypanosoma brucei rhodesiense OR nagana OR leishmaniasis OR visceral leishmaniasis OR cutaneous leishmaniasis OR mucocutaneous leishmaniasis OR kala-azar OR phlebotomine sandflies OR leprosy OR hansen's disease OR mycobacterium leprae OR lymphatic filariasis OR elephantiasis OR nematodes OR roundworms)) OR (tw:(roundworms or nematodes)) OR (tw:(wuchereria bancrofti OR brugia malayi OR brugia timori OR culex OR anopheles OR aedes OR onchocerciasis OR river blindness OR onchocerca volvulus OR simulium OR blackflies OR rabies OR schistosomiasis OR parasitic worms OR blood fluke OR trematode worms OR Intestinal schistosomiasis OR schistosoma mansoni OR schistosoma japonicum OR schistosoma mekongi OR schistosoma guineensis OR schistosoma intercalatum OR urogenital schistosomiasis OR schistosoma haematobium OR soil-transmitted helminthiases OR soil-transmitted helminth infection OR roundworm OR ascaris lumbricoides OR whipworm OR trichuris trichiura OR hookworms in humans OR hookworms OR necator americanus OR ancylostoma duodenale OR taeniasis/cysticercosis OR taeniasis OR cysticercosis OR tapeworm OR taenia solium OR pork tapeworm OR taenia saginata OR beef tapeworm OR taenia asiatica OR cysticerci OR trachoma OR chlamydia trachomatis OR endemic treponematoses OR yaws OR treponema pallidum OR pertenue)) OR (tw:(neglected tropical diseases OR tropical diseases OR neglected diseases OR ntds OR yaws OR chromomycosis OR deep mycosis OR scabies OR ectoparasite OR snakebite OR yaws-endemic treponematoses OR chromoblastomycosis OR deep mycoses OR ectoparasites OR snakebite envenoming))))

LMIC

(tw:((tw:(third world OR low income country OR middle income country OR lower middle income country OR upper middle income country)) OR (tw:(afghanistan)) OR (tw:(benin)) OR (tw:(burkina faso)) OR (tw:(burundi)) OR (tw:(central african republic)) OR (tw:(chad)) OR (tw:(democratic republic congo)) OR (tw:(eritrea)) OR (tw:(ethiopia)) OR (tw:(gambia)) OR (tw:(guinea)) OR (tw:(guinea-bissau)) OR (tw:(haiti)) OR (tw:(korea)) OR (tw:(liberia)) OR (tw:(madagascar)) OR (tw:(malawi)) OR (tw:(mozambique)) OR (tw:(nepal)) OR (tw:(niger)) OR (tw:(rwanda)) OR (tw:(sierra leone)) OR (tw:(somalia)) OR (tw:(south sudan)) OR (tw:(syrian arab republic)) OR (tw:(tajikistan)) OR (tw:(tanzania)) OR (tw:(togo)) OR (tw:(uganda)) OR (tw:(yemen)) OR (tw:(mali)))) OR (tw:((tw:(angola)) OR (tw:(bangladesh)) OR (tw:(bhutan)) OR (tw:(bolivia)) OR (tw:(cape verde)) OR (tw:(cambodia)) OR (tw:(cameroon)) OR (tw:(comoros)) OR (tw:(congo)) OR (tw:(cote d`ivoire)) OR (tw:(djibouti)) OR (tw:(egypt)) OR (tw:(el salvador)) OR (tw:(ghana)) OR (tw:(honduras)) OR (tw:(india)) OR (tw:(papua new guinea)) OR (tw:(indonesia)) OR (tw:(kenya)) OR (tw:(kiribati)) OR (tw:(kyrgyzstan)) OR (tw:(laos)) OR (tw:(lesotho)) OR (tw:(mauritania)) OR (tw:(federated states of micronesia)) OR (tw:(moldova)) OR (tw:(mongolia)) OR (tw:(morocco)) OR (tw:(myanmar)) OR (tw:(nicaragua)) OR (tw:(nigeria)) OR (tw:(pakistan)) OR (tw:(philippines)) OR (tw:(sao tome and principe)) OR (tw:(senegal)) OR (tw:(solomon islands)) OR (tw:(sudan)) OR (tw:(swaziland)) OR (tw:(timor-leste)) OR (tw:(tunisia)) OR (tw:(ukraine)) OR (tw:(uzbekistan)) OR (tw:(vanuatu)) OR (tw:(vietnam)) OR (tw:(zambia)) OR (tw:(zimbabwe)) OR (tw:(palestine)))) OR (tw:((tw:(albania)) OR (tw:(algeria)) OR (tw:(american samoa)) OR (tw:(argentina)) OR (tw:(armenia)) OR (tw:(azerbaijan)) OR (tw:(belarus)) OR (tw:(belize)) OR (tw:(bosnia and herzegovina)) OR (tw:(botswana)) OR (tw:(brazil)) OR (tw:(bulgaria)) OR (tw:(china)) OR (tw:(colombia)) OR (tw:(costa rica)) OR (tw:(cuba)) OR (tw:(dominica)) OR (tw:(dominican republic)) OR (tw:(equatorial guinea)) OR (tw:(ecuador)) OR (tw:(fiji)) OR (tw:(gabon)) OR (tw:(georgia (republic))) OR (tw:(grenada)) OR (tw:(guatemala)) OR (tw:(guyana)) OR (tw:(iran)) OR (tw:(iraq)) OR (tw:(jamaica)) OR (tw:(jordan)) OR (tw:(kazakhstan)) OR (tw:(kosovo)) OR (tw:(lebanon)) OR (tw:(libyan arab jamahiriya)) OR (tw:(macedonia (republic))) OR (tw:(malaysia)) OR (tw:(maldives)) OR (tw:(marshall islands)) OR (tw:(mauritius)) OR (tw:(mexico)) OR (tw:(montenegro (republic))) OR (tw:(namibia)) OR (tw:(nauru)) OR (tw:(paraguay)) OR (tw:(peru)) OR (tw:(romania)) OR (tw:(russian federation)) OR (tw:(samoan islands)) OR (tw:(serbia)) OR (tw:(sri lanka)) OR (tw:(south africa)) OR (tw:(saint lucia)) OR (tw:(saint vincent and the grenadines)) OR (tw:(suriname)) OR (tw:(thailand)) OR (tw:(tonga)) OR (tw:(turkey (republic))) OR (tw:(turkmenistan)) OR (tw:(tuvalu)) OR (tw:(venezuela))))

Type of studies

(tw:(controlled clinical trial)) OR (tw:(crossover procedure)) OR (tw:(double blind procedure)) OR (tw:(randomized controlled trial)) OR (tw:(single blind procedure)) OR (tw:(comparative study)) OR (tw:(controlled study)) OR (tw:(experimental study)) OR (tw:(feasibility study)) OR (tw:(field study)) OR (tw:(observational study)) OR (tw:(panel study)) OR (tw:(prevention study)) OR (tw:(quality improvement study)) OR (tw:(quasi experimental study)) OR (tw:(randomised controlled study OR randomised controlled trial OR rct OR non-rct)) OR (tw:(experimental studies OR feasibility studies OR field studies OR non experimental studies OR non experimental study OR nonexperimental studies OR nonexperimental study OR observation studies OR

observation study OR observational studies OR panel study)) OR (tw:(prevention trial OR preventive study OR preventive trial OR quality improvement studies OR quasiexperimental study OR replication studies OR trend studies OR twin studies OR twins study OR validation studies)) OR (tw:(replication study OR trend study OR twin study OR validation study))

Process Evaluation + NCDS/NTDs + LMIC + type of studies
(tw:(health care quality OR program evaluation OR outcome assessment OR treatment outcome OR clinical governance OR health care OR healthcare OR quality assurance OR quality indicator OR access OR evaluation OR process assessment OR quality of care research OR quality of health care OR outcome management OR patient outcome OR standard of care OR causal mechanisms OR process evaluation OR fidelity OR program evaluation OR outcome assessment OR treatment outcome OR clinical governance OR health care OR healthcare OR quality assurance OR quality indicator OR access OR evaluation OR process assessment OR quality of care research OR quality of health care OR outcome management OR patient outcome OR standard of care OR causal mechanisms OR process evaluation OR fidelity)) AND (tw:((tw:((tw:((tw:((tw:((tw:(non communicable disease)) OR (tw:(non-communicable disease)) OR (tw:(non-communicable diseases)) OR (tw:(noncommunicable disease)) OR (tw:(noncommunicable diseases))))))) OR (tw:(non insulin dependent diabetes mellitus)) OR (tw:(insulin dependent diabetes mellitus)) OR (tw:(cardiovascular disease)) OR (tw:(chronic obstructive lung disease)) OR (tw:(chronic kidney failure)) OR (tw:(type 2 diabetes mellitus)) OR (tw:(type 1 diabetes mellitus)) OR (tw:(coronary heart disease)) OR (tw:(chronic obstructive pulmonary disease)) OR (tw:(chronic kidney disease)))) OR (tw:((tw:(buruli ulcer OR mycobacterium ulcerans OR chagas disease OR american trypanosomiasis OR trypanosoma cruzi OR dengue OR mosquito OR chikungunya OR dracunculiasis OR guinea worm disease OR dracunculus medinensis OR echinococcosis OR echinococcus granulosus OR cystic echinococcosis OR hydatidosis OR hydatid disease OR alveolar echinococcosis OR echinococcus multilocularis OR polycystic echinococcosis OR echinococcus vogeli OR unicystic echinococcosis OR echinococcus oligarthrus OR foodborne trematodiases OR trematode worms OR flukes OR freshwater snails OR freshwater fish OR clonorchis sinensis OR opisthorchis viverrini OR opisthorchis Felineus OR fasciola hepatica OR fasciola gigantica OR paragonimus OR human african trypanosomiasis OR sleeping sickness OR trypanosoma brucei gambiense OR tsetse flies OR glossina genus OR trypanosoma brucei rhodesiense OR nagana OR leishmaniasis OR visceral leishmaniasis OR cutaneous leishmaniasis OR mucocutaneous leishmaniasis OR kala-azar OR phlebotomine sandflies OR leprosy OR hansen's disease OR mycobacterium leprae OR lymphatic filariasis OR elephantiasis OR nematodes OR roundworms)) OR (tw:(roundworms or nematodes)) OR (tw:(wuchereria bancrofti OR brugia malayi OR brugia timori OR culex OR anopheles OR aedes OR onchocerciasis OR river blindness OR onchocerca volvulus OR simulium OR blackflies OR rabies OR schistosomiasis OR parasitic worms OR blood fluke OR trematode worms OR Intestinal schistosomiasis OR schistosoma mansoni OR schistosoma japonicum OR schistosoma mekongi OR schistosoma guineensis OR schistosoma intercalatum OR urogenital schistosomiasis OR schistosoma haematobium OR soil-transmitted helminthiases OR soil-transmitted helminth infection OR roundworm OR ascaris lumbricoides OR whipworm OR trichuris trichiura OR hookworms in humans OR hookworms OR necator americanus OR ancylostoma duodenale OR taeniasis/cysticercosis OR taeniasis OR cysticercosis

OR tapeworm OR taenia solium OR pork tapeworm OR taenia saginata OR beef tapeworm OR taenia asiatica OR cysticerci OR trachoma OR chlamydia trachomatis OR endemic treponematoses OR yaws OR treponema pallidum OR pertenue)) OR (tw:(neglected tropical diseases OR tropical diseases OR neglected diseases OR ntds OR yaws OR chromomycosis OR deep mycosis OR scabies OR ectoparasite OR snakebite OR yaws-endemic treponematoses OR chromoblastomycosis OR deep mycoses OR ectoparasites OR snakebite envenoming)))))) AND (tw:((tw:((tw:(third world OR low income country OR middle income country OR lower middle income country OR upper middle income country)) OR (tw:(afghanistan)) OR (tw:(benin)) OR (tw:(burkina faso)) OR (tw:(burundi)) OR (tw:(central african republic)) OR (tw:(chad)) OR (tw:(democratic republic congo)) OR (tw:(eritrea)) OR (tw:(ethiopia)) OR (tw:(gambia)) OR (tw:(guinea)) OR (tw:(guinea-bissau)) OR (tw:(haiti)) OR (tw:(korea)) OR (tw:(liberia)) OR (tw:(madagascar)) OR (tw:(malawi)) OR (tw:(mozambique)) OR (tw:(nepal)) OR (tw:(niger)) OR (tw:(rwanda)) OR (tw:(sierra leone)) OR (tw:(somalia)) OR (tw:(south sudan)) OR (tw:(syrian arab republic)) OR (tw:(tajikistan)) OR (tw:(tanzania)) OR (tw:(togo)) OR (tw:(uganda)) OR (tw:(yemen)) OR (tw:(mali)))) OR (tw:((tw:(angola)) OR (tw:(bangladesh)) OR (tw:(bhutan)) OR (tw:(bolivia)) OR (tw:(cape verde)) OR (tw:(cambodia)) OR (tw:(cameroon)) OR (tw:(comoros)) OR (tw:(congo)) OR (tw:(cote d`ivoire)) OR (tw:(djibouti)) OR (tw:(egypt)) OR (tw:(el salvador)) OR (tw:(ghana)) OR (tw:(honduras)) OR (tw:(india)) OR (tw:(papua new guinea)) OR (tw:(indonesia)) OR (tw:(kenya)) OR (tw:(kiribati)) OR (tw:(kyrgyzstan)) OR (tw:(laos)) OR (tw:(lesotho)) OR (tw:(mauritania)) OR (tw:(federated states of micronesia)) OR (tw:(moldova)) OR (tw:(mongolia)) OR (tw:(morocco)) OR (tw:(myanmar)) OR (tw:(nicaragua)) OR (tw:(nigeria)) OR (tw:(pakistan)) OR (tw:(philippines)) OR (tw:(sao tome and principe)) OR (tw:(senegal)) OR (tw:(solomon islands)) OR (tw:(sudan)) OR (tw:(swaziland)) OR (tw:(timor-leste)) OR (tw:(tunisia)) OR (tw:(ukraine)) OR (tw:(uzbekistan)) OR (tw:(vanuatu)) OR (tw:(vietnam)) OR (tw:(zambia)) OR (tw:(zimbabwe)) OR (tw:(palestine)))) OR (tw:((tw:(albania)) OR (tw:(algeria)) OR (tw:(american samoa)) OR (tw:(argentina)) OR (tw:(armenia)) OR (tw:(azerbaijan)) OR (tw:(belarus)) OR (tw:(belize)) OR (tw:(bosnia and herzegovina)) OR (tw:(botswana)) OR (tw:(brazil)) OR (tw:(bulgaria)) OR (tw:(china)) OR (tw:(colombia)) OR (tw:(costa rica)) OR (tw:(cuba)) OR (tw:(dominica)) OR (tw:(dominican republic)) OR (tw:(equatorial guinea)) OR (tw:(ecuador)) OR (tw:(fiji)) OR (tw:(gabon)) OR (tw:(georgia (republic))) OR (tw:(grenada)) OR (tw:(guatemala)) OR (tw:(guyana)) OR (tw:(iran)) OR (tw:(iraq)) OR (tw:(jamaica)) OR (tw:(jordan)) OR (tw:(kazakhstan)) OR (tw:(kosovo)) OR (tw:(lebanon)) OR (tw:(libyan arab jamahiriya)) OR (tw:(macedonia (republic))) OR (tw:(malaysia)) OR (tw:(maldives)) OR (tw:(marshall islands)) OR (tw:(mauritius)) OR (tw:(mexico)) OR (tw:(montenegro (republic))) OR (tw:(namibia)) OR (tw:(nauru)) OR (tw:(paraguay)) OR (tw:(peru)) OR (tw:(romania)) OR (tw:(russian federation)) OR (tw:(samoan islands)) OR (tw:(serbia)) OR (tw:(sri lanka)) OR (tw:(south africa)) OR (tw:(saint lucia)) OR (tw:(saint vincent and the grenadines)) OR (tw:(suriname)) OR (tw:(thailand)) OR (tw:(tonga)) OR (tw:(turkey (republic))) OR (tw:(turkmenistan)) OR (tw:(tuvalu)) OR (tw:(venezuela)))))) AND (tw:((tw:(controlled clinical trial)) OR (tw:(crossover procedure)) OR (tw:(double blind procedure)) OR (tw:(randomized controlled trial)) OR (tw:(single blind procedure)) OR (tw:(comparative study)) OR (tw:(controlled study)) OR (tw:(experimental study)) OR (tw:(feasibility study)) OR (tw:(field study)) OR (tw:(observational study)) OR (tw:(panel study)) OR (tw:(prevention study)) OR (tw:(quality improvement study)) OR (tw:(quasi

experimental study)) OR (tw:(randomised controlled study OR randomised controlled trial OR rct OR non-rct)) OR (tw:(experimental studies OR feasibility studies OR field studies OR non experimental studies OR non experimental study OR nonexperimental studies OR nonexperimental study OR observation studies OR observation study OR observational studies OR panel study)) OR (tw:(prevention trial OR preventive study OR preventive trial OR quality improvement studies OR quasiexperimental study OR replication studies OR trend studies OR twin studies OR twins study OR validation studies)) OR (tw:(replication study OR trend study OR twin study OR validation study))))

480 281119

488 090120

Virtual Health Library

Process Evaluation

health care quality OR program evaluation OR outcome assessment OR treatment outcome OR clinical governance OR health care OR healthcare OR quality assurance OR quality indicator OR access OR evaluation OR process assessment OR quality of care research OR quality of health care OR outcome management OR patient outcome OR standard of care OR causal mechanisms OR process evaluation OR fidelity OR program evaluation OR outcome assessment OR treatment outcome OR clinical governance OR health care OR healthcare OR quality assurance OR quality indicator OR access OR evaluation OR process assessment OR quality of care research OR quality of health care OR outcome management OR patient outcome OR standard of care OR causal mechanisms OR process evaluation OR fidelity

NTDs + NCDs
(tw:((tw:((tw:((tw:((tw:(non communicable disease)) OR (tw:(non-communicable disease)) OR (tw:(non-communicable diseases)) OR (tw:(noncommunicable disease)) OR (tw:(noncommunicable diseases))))))) OR (tw:(non insulin dependent diabetes mellitus)) OR (tw:(insulin dependent diabetes mellitus)) OR (tw:(cardiovascular disease)) OR (tw:(chronic obstructive lung disease)) OR (tw:(chronic kidney failure)) OR (tw:(type 2 diabetes mellitus)) OR (tw:(type 1 diabetes mellitus)) OR (tw:(coronary heart disease)) OR (tw:(chronic obstructive pulmonary disease)) OR (tw:(chronic kidney disease)))) OR (tw:((tw:(buruli ulcer OR mycobacterium ulcerans OR chagas disease OR american trypanosomiasis OR trypanosoma cruzi OR dengue OR mosquito OR chikungunya OR dracunculiasis OR guinea worm disease OR dracunculus medinensis OR echinococcosis OR echinococcus granulosus OR cystic echinococcosis OR hydatidosis OR hydatid disease OR alveolar echinococcosis OR echinococcus multilocularis OR polycystic echinococcosis OR echinococcus vogeli OR unicystic echinococcosis OR echinococcus oligarthrus OR foodborne trematodiases OR trematode worms OR flukes OR freshwater snails OR freshwater fish OR clonorchis sinensis OR opisthorchis viverrini OR opisthorchis Felineus OR fasciola hepatica OR fasciola gigantica OR paragonimus OR human african trypanosomiasis OR sleeping sickness OR trypanosoma brucei gambiense OR tsetse flies OR glossina genus OR trypanosoma brucei rhodesiense OR nagana OR leishmaniasis OR visceral leishmaniasis OR cutaneous leishmaniasis OR mucocutaneous leishmaniasis OR kala-azar OR phlebotomine sandflies OR leprosy OR hansen's disease OR mycobacterium leprae OR lymphatic filariasis OR elephantiasis OR nematodes OR roundworms)) OR (tw:(roundworms or nematodes)) OR (tw:(wuchereria bancrofti OR brugia malayi OR brugia timori OR culex OR anopheles OR aedes OR onchocerciasis OR river blindness OR onchocerca volvulus OR simulium OR blackflies OR rabies OR schistosomiasis OR parasitic worms OR blood fluke OR trematode worms OR Intestinal schistosomiasis OR schistosoma mansoni OR schistosoma japonicum OR schistosoma mekongi OR schistosoma guineensis OR schistosoma intercalatum OR urogenital schistosomiasis OR schistosoma haematobium OR soil-transmitted helminthiases OR soil-transmitted helminth infection OR roundworm OR ascaris lumbricoides OR whipworm OR trichuris trichiura OR hookworms in humans OR hookworms OR necator americanus OR ancylostoma duodenale OR taeniasis/cysticercosis OR taeniasis OR cysticercosis OR tapeworm OR taenia solium OR pork tapeworm OR taenia saginata OR beef tapeworm OR taenia asiatica OR cysticerci OR trachoma OR chlamydia trachomatis OR endemic treponematoses OR yaws OR treponema pallidum OR pertenue)) OR (tw:(neglected tropical diseases OR tropical diseases OR neglected diseases OR ntds OR yaws OR chromomycosis OR deep mycosis OR scabies OR ectoparasite OR

snakebite OR yaws-endemic treponematoses OR chromoblastomycosis OR deep mycoses OR ectoparasites OR snakebite envenoming))))

LMIC

(tw:((tw:(third world OR low income country OR middle income country OR lower middle income country OR upper middle income country)) OR (tw:(afghanistan)) OR (tw:(benin)) OR (tw:(burkina faso)) OR (tw:(burundi)) OR (tw:(central african republic)) OR (tw:(chad)) OR (tw:(democratic republic congo)) OR (tw:(eritrea)) OR (tw:(ethiopia)) OR (tw:(gambia)) OR (tw:(guinea)) OR (tw:(guinea-bissau)) OR (tw:(haiti)) OR (tw:(korea)) OR (tw:(liberia)) OR (tw:(madagascar)) OR (tw:(malawi)) OR (tw:(mozambique)) OR (tw:(nepal)) OR (tw:(niger)) OR (tw:(rwanda)) OR (tw:(sierra leone)) OR (tw:(somalia)) OR (tw:(south sudan)) OR (tw:(syrian arab republic)) OR (tw:(tajikistan)) OR (tw:(tanzania)) OR (tw:(togo)) OR (tw:(uganda)) OR (tw:(yemen)) OR (tw:(mali)))) OR (tw:((tw:(angola)) OR (tw:(bangladesh)) OR (tw:(bhutan)) OR (tw:(bolivia)) OR (tw:(cape verde)) OR (tw:(cambodia)) OR (tw:(cameroon)) OR (tw:(comoros)) OR (tw:(congo)) OR (tw:(cote d`ivoire)) OR (tw:(djibouti)) OR (tw:(egypt)) OR (tw:(el salvador)) OR (tw:(ghana)) OR (tw:(honduras)) OR (tw:(india)) OR (tw:(papua new guinea)) OR (tw:(indonesia)) OR (tw:(kenya)) OR (tw:(kiribati)) OR (tw:(kyrgyzstan)) OR (tw:(laos)) OR (tw:(lesotho)) OR (tw:(mauritania)) OR (tw:(federated states of micronesia)) OR (tw:(moldova)) OR (tw:(mongolia)) OR (tw:(morocco)) OR (tw:(myanmar)) OR (tw:(nicaragua)) OR (tw:(nigeria)) OR (tw:(pakistan)) OR (tw:(philippines)) OR (tw:(sao tome and principe)) OR (tw:(senegal)) OR (tw:(solomon islands)) OR (tw:(sudan)) OR (tw:(swaziland)) OR (tw:(timor-leste)) OR (tw:(tunisia)) OR (tw:(ukraine)) OR (tw:(uzbekistan)) OR (tw:(vanuatu)) OR (tw:(vietnam)) OR (tw:(zambia)) OR (tw:(zimbabwe)) OR (tw:(palestine)))) OR (tw:((tw:(albania)) OR (tw:(algeria)) OR (tw:(american samoa)) OR (tw:(argentina)) OR (tw:(armenia)) OR (tw:(azerbaijan)) OR (tw:(belarus)) OR (tw:(belize)) OR (tw:(bosnia and herzegovina)) OR (tw:(botswana)) OR (tw:(brazil)) OR (tw:(bulgaria)) OR (tw:(china)) OR (tw:(colombia)) OR (tw:(costa rica)) OR (tw:(cuba)) OR (tw:(dominica)) OR (tw:(dominican republic)) OR (tw:(equatorial guinea)) OR (tw:(ecuador)) OR (tw:(fiji)) OR (tw:(gabon)) OR (tw:(georgia (republic))) OR (tw:(grenada)) OR (tw:(guatemala)) OR (tw:(guyana)) OR (tw:(iran)) OR (tw:(iraq)) OR (tw:(jamaica)) OR (tw:(jordan)) OR (tw:(kazakhstan)) OR (tw:(kosovo)) OR (tw:(lebanon)) OR (tw:(libyan arab jamahiriya)) OR (tw:(macedonia (republic))) OR (tw:(malaysia)) OR (tw:(maldives)) OR (tw:(marshall islands)) OR (tw:(mauritius)) OR (tw:(mexico)) OR (tw:(montenegro (republic))) OR (tw:(namibia)) OR (tw:(nauru)) OR (tw:(paraguay)) OR (tw:(peru)) OR (tw:(romania)) OR (tw:(russian federation)) OR (tw:(samoan islands)) OR (tw:(serbia)) OR (tw:(sri lanka)) OR (tw:(south africa)) OR (tw:(saint lucia)) OR (tw:(saint vincent and the grenadines)) OR (tw:(suriname)) OR (tw:(thailand)) OR (tw:(tonga)) OR (tw:(turkey (republic))) OR (tw:(turkmenistan)) OR (tw:(tuvalu)) OR (tw:(venezuela))))

Type of studies

(tw:(controlled clinical trial)) OR (tw:(crossover procedure)) OR (tw:(double blind procedure)) OR (tw:(randomized controlled trial)) OR (tw:(single blind procedure)) OR (tw:(comparative study)) OR (tw:(controlled study)) OR (tw:(experimental study)) OR (tw:(feasibility study)) OR (tw:(field study)) OR (tw:(observational study)) OR

(tw:(panel study)) OR (tw:(prevention study)) OR (tw:(quality improvement study)) OR (tw:(quasi experimental study)) OR (tw:(randomised controlled study OR randomised controlled trial OR rct OR non-rct)) OR (tw:(experimental studies OR feasibility studies OR field studies OR non experimental studies OR non experimental study OR nonexperimental studies OR nonexperimental study OR observation studies OR observation study OR observational studies OR panel study)) OR (tw:(prevention trial OR preventive study OR preventive trial OR quality improvement studies OR quasiexperimental study OR replication studies OR trend studies OR twin studies OR twins study OR validation studies)) OR (tw:(replication study OR trend study OR twin study OR validation study))

Process Evaluation + NCDS/NTDs + LMIC + type of studies

(tw:(health care quality OR program evaluation OR outcome assessment OR treatment outcome OR clinical governance OR health care OR healthcare OR quality assurance OR quality indicator OR access OR evaluation OR process assessment OR quality of care research OR quality of health care OR outcome management OR patient outcome OR standard of care OR causal mechanisms OR process evaluation OR fidelity OR program evaluation OR outcome assessment OR treatment outcome OR clinical governance OR health care OR healthcare OR quality assurance OR quality indicator OR access OR evaluation OR process assessment OR quality of care research OR quality of health care OR outcome management OR patient outcome OR standard of care OR causal mechanisms OR process evaluation OR fidelity)) AND (tw:((tw:((tw:((tw:((tw:((tw:(non communicable disease)) OR (tw:(non-communicable disease)) OR (tw:(non-communicable diseases)) OR (tw:(noncommunicable disease)) OR (tw:(noncommunicable diseases)))))))) OR (tw:(non insulin dependent diabetes mellitus)) OR (tw:(insulin dependent diabetes mellitus)) OR (tw:(cardiovascular disease)) OR (tw:(chronic obstructive lung disease)) OR (tw:(chronic kidney failure)) OR (tw:(type 2 diabetes mellitus)) OR (tw:(type 1 diabetes mellitus)) OR (tw:(coronary heart disease)) OR (tw:(chronic obstructive pulmonary disease)) OR (tw:(chronic kidney disease)))) OR (tw:((tw:(buruli ulcer OR mycobacterium ulcerans OR chagas disease OR american trypanosomiasis OR trypanosoma cruzi OR dengue OR mosquito OR chikungunya OR dracunculiasis OR guinea worm disease OR dracunculus medinensis OR echinococcosis OR echinococcus granulosus OR cystic echinococcosis OR hydatidosis OR hydatid disease OR alveolar echinococcosis OR echinococcus multilocularis OR polycystic echinococcosis OR echinococcus vogeli OR unicystic echinococcosis OR echinococcus oligarthrus OR foodborne trematodiases OR trematode worms OR flukes OR freshwater snails OR freshwater fish OR clonorchis sinensis OR opisthorchis viverrini OR opisthorchis Felineus OR fasciola hepatica OR fasciola gigantica OR paragonimus OR human african trypanosomiasis OR sleeping sickness OR trypanosoma brucei gambiense OR tsetse flies OR glossina genus OR trypanosoma brucei rhodesiense OR nagana OR leishmaniasis OR visceral leishmaniasis OR cutaneous leishmaniasis OR mucocutaneous leishmaniasis OR kala-azar OR phlebotomine sandflies OR leprosy OR hansen's disease OR mycobacterium leprae OR lymphatic filariasis OR elephantiasis OR nematodes OR roundworms)) OR (tw:(roundworms or nematodes)) OR (tw:(wuchereria bancrofti OR brugia malayi OR brugia timori OR culex OR anopheles OR aedes OR onchocerciasis OR river blindness OR onchocerca volvulus OR simulium OR blackflies OR rabies OR schistosomiasis OR parasitic worms OR blood fluke OR trematode worms OR Intestinal schistosomiasis

OR schistosoma mansoni OR schistosoma japonicum OR schistosoma mekongi OR schistosoma guineensis OR schistosoma intercalatum OR urogenital schistosomiasis OR schistosoma haematobium OR soil-transmitted helminthiases OR soil-transmitted helminth infection OR roundworm OR ascaris lumbricoides OR whipworm OR trichuris trichiura OR hookworms in humans OR hookworms OR necator americanus OR ancylostoma duodenale OR taeniasis/cysticercosis OR taeniasis OR cysticercosis OR tapeworm OR taenia solium OR pork tapeworm OR taenia saginata OR beef tapeworm OR taenia asiatica OR cysticerci OR trachoma OR chlamydia trachomatis OR endemic treponematoses OR yaws OR treponema pallidum OR pertenue)) OR (tw:(neglected tropical diseases OR tropical diseases OR neglected diseases OR ntds OR yaws OR chromomycosis OR deep mycosis OR scabies OR ectoparasite OR snakebite OR yaws-endemic treponematoses OR chromoblastomycosis OR deep mycoses OR ectoparasites OR snakebite envenoming)))))) AND (tw:((tw:((tw:(third world OR low income country OR middle income country OR lower middle income country OR upper middle income country)) OR (tw:(afghanistan)) OR (tw:(benin)) OR (tw:(burkina faso)) OR (tw:(burundi)) OR (tw:(central african republic)) OR (tw:(chad)) OR (tw:(democratic republic congo)) OR (tw:(eritrea)) OR (tw:(ethiopia)) OR (tw:(gambia)) OR (tw:(guinea)) OR (tw:(guinea-bissau)) OR (tw:(haiti)) OR (tw:(korea)) OR (tw:(liberia)) OR (tw:(madagascar)) OR (tw:(malawi)) OR (tw:(mozambique)) OR (tw:(nepal)) OR (tw:(niger)) OR (tw:(rwanda)) OR (tw:(sierra leone)) OR (tw:(somalia)) OR (tw:(south sudan)) OR (tw:(syrian arab republic)) OR (tw:(tajikistan)) OR (tw:(tanzania)) OR (tw:(togo)) OR (tw:(uganda)) OR (tw:(yemen)) OR (tw:(mali)))) OR (tw:((tw:(angola)) OR (tw:(bangladesh)) OR (tw:(bhutan)) OR (tw:(bolivia)) OR (tw:(cape verde)) OR (tw:(cambodia)) OR (tw:(cameroon)) OR (tw:(comoros)) OR (tw:(congo)) OR (tw:(cote d`ivoire)) OR (tw:(djibouti)) OR (tw:(egypt)) OR (tw:(el salvador)) OR (tw:(ghana)) OR (tw:(honduras)) OR (tw:(india)) OR (tw:(papua new guinea)) OR (tw:(indonesia)) OR (tw:(kenya)) OR (tw:(kiribati)) OR (tw:(kyrgyzstan)) OR (tw:(laos)) OR (tw:(lesotho)) OR (tw:(mauritania)) OR (tw:(federated states of micronesia)) OR (tw:(moldova)) OR (tw:(mongolia)) OR (tw:(morocco)) OR (tw:(myanmar)) OR (tw:(nicaragua)) OR (tw:(nigeria)) OR (tw:(pakistan)) OR (tw:(philippines)) OR (tw:(sao tome and principe)) OR (tw:(senegal)) OR (tw:(solomon islands)) OR (tw:(sudan)) OR (tw:(swaziland)) OR (tw:(timor-leste)) OR (tw:(tunisia)) OR (tw:(ukraine)) OR (tw:(uzbekistan)) OR (tw:(vanuatu)) OR (tw:(vietnam)) OR (tw:(zambia)) OR (tw:(zimbabwe)) OR (tw:(palestine)))) OR (tw:((tw:(albania)) OR (tw:(algeria)) OR (tw:(american samoa)) OR (tw:(argentina)) OR (tw:(armenia)) OR (tw:(azerbaijan)) OR (tw:(belarus)) OR (tw:(belize)) OR (tw:(bosnia and herzegovina)) OR (tw:(botswana)) OR (tw:(brazil)) OR (tw:(bulgaria)) OR (tw:(china)) OR (tw:(colombia)) OR (tw:(costa rica)) OR (tw:(cuba)) OR (tw:(dominica)) OR (tw:(dominican republic)) OR (tw:(equatorial guinea)) OR (tw:(ecuador)) OR (tw:(fiji)) OR (tw:(gabon)) OR (tw:(georgia (republic))) OR (tw:(grenada)) OR (tw:(guatemala)) OR (tw:(guyana)) OR (tw:(iran)) OR (tw:(iraq)) OR (tw:(jamaica)) OR (tw:(jordan)) OR (tw:(kazakhstan)) OR (tw:(kosovo)) OR (tw:(lebanon)) OR (tw:(libyan arab jamahiriya)) OR (tw:(macedonia (republic))) OR (tw:(malaysia)) OR (tw:(maldives)) OR (tw:(marshall islands)) OR (tw:(mauritius)) OR (tw:(mexico)) OR (tw:(montenegro (republic))) OR (tw:(namibia)) OR (tw:(nauru)) OR (tw:(paraguay)) OR (tw:(peru)) OR (tw:(romania)) OR (tw:(russian federation)) OR (tw:(samoan islands)) OR (tw:(serbia)) OR (tw:(sri lanka)) OR (tw:(south africa)) OR (tw:(saint lucia)) OR (tw:(saint vincent and the grenadines)) OR (tw:(suriname)) OR (tw:(thailand

)) OR (tw:(tonga)) OR (tw:(turkey (republic))) OR (tw:(turkmenistan)) OR (tw:(tuvalu)) OR (tw:(venezuela))))))

3.624 281119
