# Supplementary material for: Factors Influencing the Implementation of Remote Delivery Strategies for Non-Communicable Disease Care in Low- and Middle-Income Countries: A Narrative Review
Source: Public Health Rev. 2022 Jun 27;43:1604583. doi: 10.3389/phrs.2022.1604583 (PMC9272771; doi:10.3389/phrs.2022.1604583)
Supplement: Supplementary file 2 [file DataSheet4.pdf]

Supplementary Material 4: Summary of study characteristics

Author, Date, Reference	Study title	Country (*level of income*[1])	Disease targeted[2]	Delivery approach(es)	Key health care services delivered through the approach
1.<br>(Abdel-All et al., 2018)	Evaluation of a training program of hypertension for accredited social health activists (ASHA) in rural India	India<br>*(LMIC)*	HTN	Community-based *(including task-sharing)*	CHWs – **support for self-management**:<br>- Education support groups (HTN and its management, promotion of healthy lifestyle)<br>- Disease monitoring: clinical measurements (BP, weight)
2.<br>(Amarchand et al., 2015)	Lessons for addressing noncommunicable diseases within a primary health-care system from the Ballabgarh project, India	India<br>*(LMIC)*	Common NCDs (HTN, DM and CAD)	Community-based	CHWs:<br>- NCD risk assessment & referral<br>- Promotion of healthy lifestyle<br>- Disease monitoring: medication adherence
3.<br>(Ameh et al., 2017)	Quality of integrated chronic disease care in rural South Africa: user and provider perspectives	South Africa<br>*(UMIC)*	Chronic diseases (HIV/ AIDS, TB, HTN, DM, COPD, asthma, epilepsy, mental health illnesses)	Community-based<br>*(including task-sharing)*	CHWs – **Assisted' self-management**:<br>- Health promotion and education<br>- Disease monitoring: BP and blood sugar monitoring assistance<br>- Home-delivery of medication
4.<br>(Barsky et al., 2019)	Analysis of the Implementation, User Perspectives, and Feedback From a Mobile Health Intervention for Individuals Living With Hypertension (DREAM-GLOBAL): Mixed Methods Study	Tanzania<br>*(LIC)*<br>(Canada *(HIC)*)	HTN	E-health<br>*(mHealth, telemonitoring)*	Mobile health intervention:<br>- Mobile phone app for trained CHW or nurses to link the mobile phone to the BP measurement device and allow transmission of the reading to the central server<br>- SMS text messaging-based system: passive (promotion of healthy lifestyle) and active (based on BP results)
5.<br>(Baumann et al., 2015)	A Demonstration of Peer Support for Ugandan Adults With Type 2 Diabetes	Uganda<br>*(LIC)*	DM (type 2)	Community-based	**Peer support** intervention by nurse-led team:<br>- Training of peers and champions, provision of materials (including a phone)<br>- Regular phone or personal contacts: assistance in applying disease management or prevention

					in daily life, emotional and social support, linkage to clinical care, and ongoing support
6.<br>(Brandt et al., 2019)	Addressing Depression Comorbid With Diabetes or Hypertension in Resource-Poor Settings: A Qualitative Study About User Perception of a Nurse-Supported Smartphone App in Peru	Peru<br>*(UMIC)*	Depression in people living with DM and / or HTN	E-health<br>*(web-based intervention)*	Nurse supported smartphone app to address depression comorbid with DM and/or HTN:<br>- App using behavioural activation approach (text and videos), and interactive elements (notification, dialogue pop-ups)<br>- Nurse dashboard to monitor participants participations
7.<br>(DePue et al., 2013)	Implementation of a Culturally Tailored Diabetes Intervention With Community Health Workers in American Samoa	American Samoa<br>*(LMIC)*	DM (type 2)	Community-based	Nurse-CHW team – **support for self-management**:<br>- Use of algorithm to determine risk profile and visit schedule<br>- Regular (individual and group) visits to patients – health promotion and education, safety monitoring and urgent referral
8.<br>(Do Valle Nascimento et al., 2017)	A pilot study of a Community Health Agent-led type 2 diabetes self-management program using Motivational Interviewing-based approaches in a public primary care center in São Paulo, Brazil	Brazil<br>*(UMIC)*	DM (type 2)	Community-based	Community health agents (CHA) – **support for self-management**:<br>- Use of motivational interviewing approaches to support DM patients' self-management as part of routine service delivery in their mandatory monthly home visits
9.<br>(Fisher et al., 2012)	Peer Support For Self-Management Of Diabetes Improved Outcomes In International Settings	Cameroon<br>*(LMIC)*<br>South Africa<br>*(UMIC)*<br>Thailand<br>(*UMIC)*<br>Uganda<br>*(LIC)*	DM (type not specified)	Community-based	**Peer support** for self-management programmes implemented in 4 countries:<br>- "Peer supporters" or "peer champions" provide support to "partners" (face to face meetings of phone contacts): disease management, healthy lifestyle<br>- Thailand: village health volunteers led meetings and supported the intervention implementation<br>- Cooking sessions, shared meals

10.<br>(Flood et al., 2017)	A quality improvement project using statistical process control methods for type 2 diabetes control in a resource-limited setting	Guatemala<br>*(LMIC)*	DM (type 2)	Community-based	**Home-based DM education programme** targeting patients and family members delivered by a nurse educator
11.<br>(Haddad et al., 2014)	A Feasibility Study of Mobile Phone Text Messaging to Support Education and Management of Type 2 Diabetes in Iraq	Iraq<br>*(LMIC)*	DM (type 2)	E-health<br>*(mHealth)*	Mobile health intervention:<br>- SMS text messages sent weekly using a website to support DM self-management. Education-related themes: diet, treatment, complication awareness, blood glucose monitoring, enhancement of clinic attendance
12.<br>(Huang et al., 2014)	Use of family member-based supervision in the management of patients with hypertension in rural china	China<br>*(UMIC)*	HTN	Community-based	**Family-based supervision programme**:<br>- A family member was selected as the observer to supervise patient's treatment, and remind patients to seek timely health care and to get their BP monitored on a regular basis
13.<br>(Jafar et al., 2016)	Control of blood pressure and risk attenuation: a public health intervention in rural Bangladesh, Pakistan, and Sri Lanka: feasibility trial results	Bangladesh<br>*(LMIC)*<br>Pakistan<br>*(LMIC)*<br>Sri Lanka<br>*(LMIC)*	HTN	Community-based	**Home health education** by government CHWs and documentation using checklist
14.<br>(Jafari et al., 2016)	Exploring educational needs and design aspects of internet-enabled patient education for persons with diabetes:<br>a qualitative interview study	Iran<br>*(UMIC)*	DM (type 2)	E-health<br>*(web-based intervention)*	The study explores educational needs and design features of personalised Internet-enabled education for patients with DM to enhance self-care management
15.<br>(Kulnawan et al., 2011)	Development of diabetes telephone-linked care system for self-management support and acceptability test among type 2 diabetic patients.	Thailand<br>*(LMIC or UMIC)*	DM (type 2)	E-health<br>*(mHealth)*	Mobile (or home) telephone health intervention:<br>- Telephone-linked care system using interactive voice response for DM self-management education and support
16.<br>(Lee J Y et al., 2015)	Diabetes telemonitoring reduces the risk of hypoglycaemia during Ramadan: a pilot randomized controlled study	Malaysia<br>*(UMIC)*	DM (type 2)	E-health<br>*(telemonitoring)*	DM telemonitoring programme:<br>- Education on DM management

					- Self-monitoring (via web-enabled glucometer) and goal setting (feedback provided when blood glucose below or above thresholds)
17.<br>(Lee Yew Kong et al., 2018)	Usability and utility evaluation of the web-based “Should I Start Insulin?” patient decision aid for patients with type 2 diabetes among older people	Malaysia<br>*(UMIC)*	DM (type 2)	E-health<br>*(web-based intervention)*	Web-based patient decision aid for insulin initiation:<br>- website with interactive features to support decision-making process
18.<br>(Maar et al., 2016)	Unpacking the Black Box: A Formative Research Approach to the Development of Theory-Driven, Evidence-Based, and Culturally Safe Text Messages in Mobile Health Interventions	Tanzania<br>*(LIC)*<br>(Canada *(HIC)*)	HTN	E-health<br>*(mHealth, telemonitoring)*	Mobile health intervention:<br>- Mobile phone app for trained CHW or nurses to link the mobile phone to the BP measurement device and allow transmission of the reading to the central server<br>- SMS text messaging-based system: passive (promotion of healthy lifestyle) and active (based on BP results)
19.<br>(Ndou et al., 2013)	A rapid assessment of a community health worker pilot programme to improve the management of hypertension and diabetes in Emfuleni sub-district of Gauteng Province, South Africa	South Africa<br>*(UMIC)*	DM (type not specified) and / or HTN	Community-based<br>*(including task-sharing)*<br>Adaptation of medicine provision	**CHW-led programme for DM and/or HTN management** – Monthly home visits:<br>- Social support, health promotion and counselling, encouragement for appropriate visits to clinic<br>- Home delivery of medication<br>- Assessment of basic clinical indicators
20.<br>(Peacock et al., 2020)	A culturally informed lower-extremity complication prevention program for people living with diabetes in south India	India<br>*(LMIC)*	DM (type not specified)	Community-based	**Lower-extremity complication prevention programme** using “Culturally informed community nursing practice” process. CHWs<br>- Community education session (group session)<br>- Foot assessment<br>- Self-care education
21.<br>(Pichayapinyo et al., 2019)	Feasibility study of automated interactive voice response telephone calls with community health nurse follow-up to improve glycaemic control in patients with type 2 diabetes	Thailand<br>*(UMIC)*	DM (type 2)	E-health<br>*(mHealth)*	Telephone health intervention to support self-management:<br>- automated interactive voice response calls to patients (weekly)

					- automated follow-up email notifications to their nurses (weekly report)<br>- Follow-up of patients as needed (by nurses)
22.<br>(Rahmawati and Bajorek, 2015)	A Community Health Worker–Based Program for Elderly People With Hypertension in Indonesia: A Qualitative Study, 2013	Indonesia<br>*(LMIC)*	HTN	Community-based<br>*(including task-sharing)*	CHW-based programme – **the Integrated Health Service Post for the Elderly**<br>(includes disease monitoring: BP check)
23.<br>(Ramachandran et al., 2018)	mDiabetes initiative using text messages to improve lifestyle and health-seeking behaviour in India	India<br>*(LMIC)*	DM (type not specified)	E-health<br>*(mHealth)*	Mobile health programme:<br>- Tailored SMS text messages sent on alternate days (promotion of healthy lifestyle, basics of DM and medication adherence)
24.<br>(Rho et al., 2017)	Comparison of the Acceptance of Telemonitoring for Glucose Management Between South Korea and China	China<br>*(UMIC)*<br>(South Korea *(HIC) – not included in this review*)	DM (type 2)	E-health<br>*(Telemonitoring, mHealth)*	Telemonitoring for glucose control:<br>- Self-monitoring of blood glucose (using a glucometer connected to a web-based system<br>- Recommendations through online messages sent by nurses<br>- Text messages or phone calls as needed
25.<br>(Saleh et al., 2018)	mHealth use for non-communicable diseases care in primary health: patients' perspective from rural settings and refugee camps	Lebanon<br>*(UMIC)*	DM (type not specified) and / or HTN	E-health<br>*(mHealth)*	Mobile health intervention:<br>- Educational SMS text messages sent weekly to patient or relatives (promotion of healthy lifestyle, medication adherence, symptoms of DM / HTN) and appointment reminders
26.<br>(Shen et al., 2017)	Can a community-based peer-led diabetes self-management programme be effective: 12-week evaluation	China<br>*(UMIC)*	DM (type 2)	Community-based	**CHWs support and peer-led** self-management programme:<br>- Community health professional-led education group classes<br>- Peer leaders-led self-efficacy enhancing group activities<br>- Supervision: bi-weekly follow-up peer leader meetings

27.<br>(Van Olmen et al., 2017)	Process evaluation of a mobile health intervention for people with diabetes in low-income countries – the implementation of the TEXT4DSM study	DRC<br>*(LIC)*<br>Cambodia<br>*(LIC)*<br>Philippines<br>*(LMIC)*	DM (type 2 or 1)	E-health<br>*(mHealth)*	Mobile health intervention to support self-management:<br>- In addition to diabetes self-management education, SMS text messages were sent to participants several times a week (healthy behaviours and disease management)
28.<br>(Lazo-Porras et al., 2020)	Foot thermometry with mHeath-based supplementation to prevent diabetic foot ulcers: A randomized controlled trial	Peru<br>*(UMIC)*	DM (type 2)	E-health<br>*(mHealth, telemonitoring)*	Foot thermometry and mobile health intervention to prevent diabetic foot ulcers:<br>- Education session on foot care<br>- Daily use of thermometry device by patients<br>- In case of alarm sign: follow-up by a nurse<br>- SMS text and voice messages sent weekly (reminder and foot-care promotion messages) to patient or caregiver via automated software

[1] Country level of income: LIC: low-income country; LMIC= lower middle-income country; UMIC= upper middle-income country; HIC= high income country

[2] Disease targeted (through the intervention): CAD= coronary artery disease; COPD= chronic obstructive pulmonary disease; DM= diabetes mellitus; HIV/AIDS= human immunodeficiency virus/acquired immunodeficiency syndrome; HTN= hypertension; NCD= non-communicable diseases; TB= tuberculosis

Other abbreviations: BP= blood pressure; CHW= community health workers
