# Supplementary figures and images for: Factors Influencing the Implementation of Remote Delivery Strategies for Non-Communicable Disease Care in Low- and Middle-Income Countries: A Narrative Review
Source: Public Health Rev. 2022 Jun 27;43:1604583. doi: 10.3389/phrs.2022.1604583 (PMC9272771; doi:10.3389/phrs.2022.1604583)

# Supplementary Material 6: Coding Framework Community Based Strategies

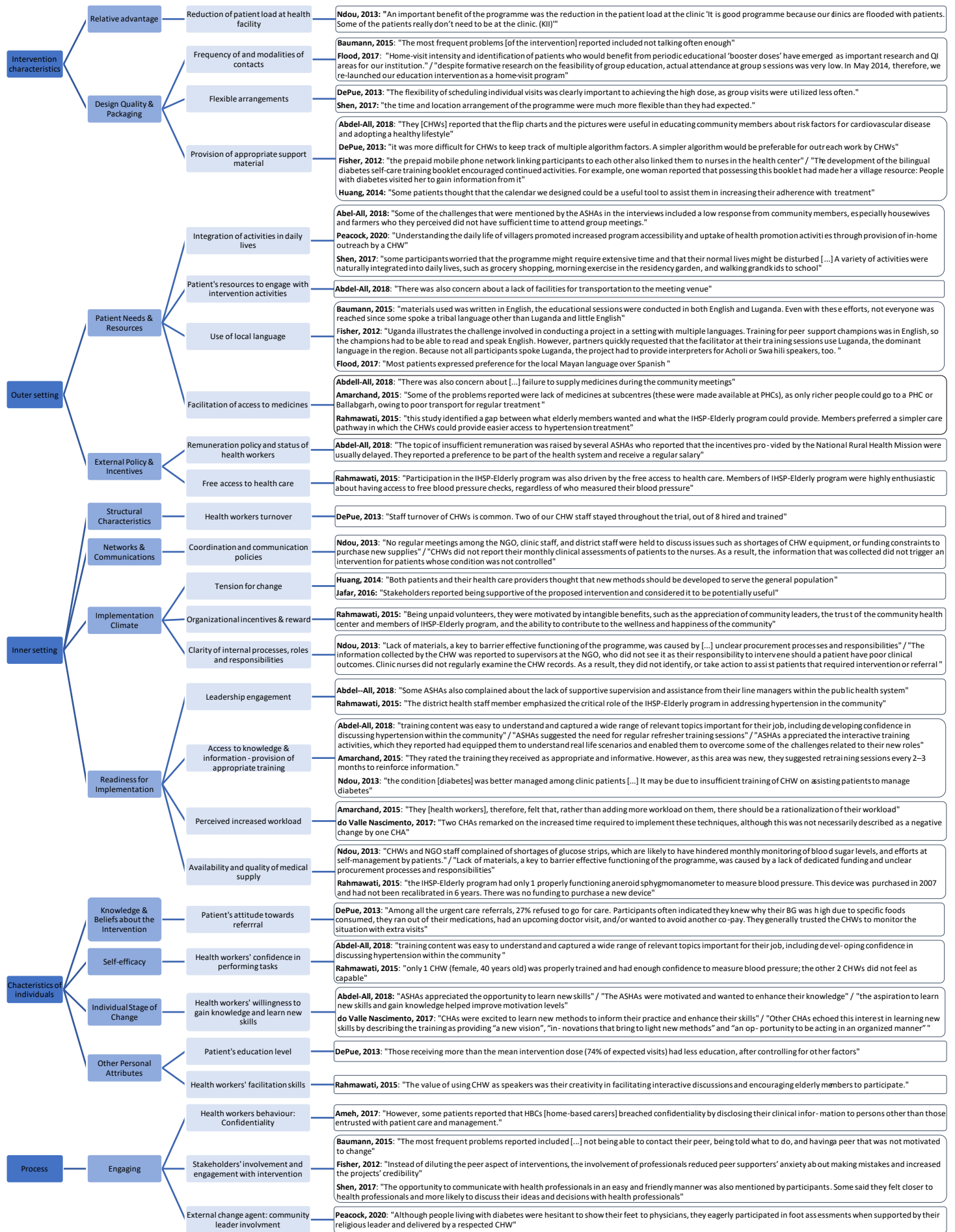

Supplement: Supplementary file 3 [file DataSheet6.pdf]

# Supplementary Material 5: Coding Framework E-Health

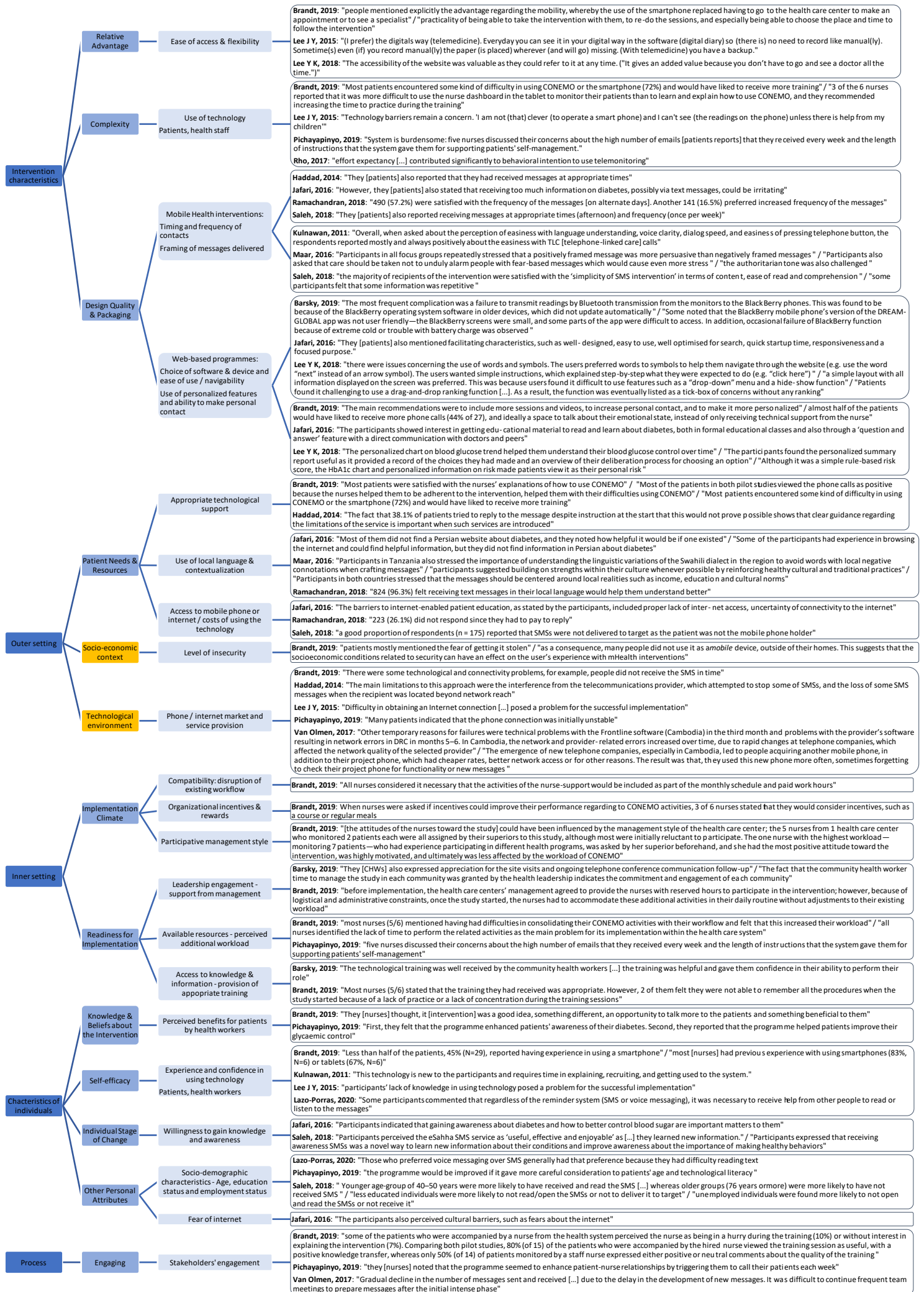

Supplement: Supplementary file 6 [file DataSheet5.pdf]
