# Supplementary material for: Factors Influencing the Implementation of Remote Delivery Strategies for Non-Communicable Disease Care in Low- and Middle-Income Countries: A Narrative Review
Source: Public Health Rev. 2022 Jun 27;43:1604583. doi: 10.3389/phrs.2022.1604583 (PMC9272771; doi:10.3389/phrs.2022.1604583)
Supplement: Supplementary file 5 [file DataSheet1.pdf]

Supplementary File 1. Search strategy from referenced systematic review on models of care for patients with hypertension and diabetes in humanitarian crises (2020)

Jaung MS, Willis R, Sharma P, Aebischer Perone S, Frederiksen S, Truppa C, et al. Models of Care for Patients with Hypertension and Diabetes in Humanitarian Crises: A Systematic Review. Health Policy Plan (2021) 36: 509. doi:10.1093/heapol/czab007 19.

Search strategy

Revised Medline Search 07 Nov 2020

1 (non?communicable disease* or chronic disease* or chronic illness* or chronic condition* or long?term condition*)

2 (hypertensi* or high blood pressure or cardiovascular disease* or heart disease* or cardiomyopath* or ((heart or cardiac or ventricular) and (isch?emia or attack or failure or dysfunction or insufficiency)))

3 (diabet* or hyperglyc?emia or blood sugar or blood glucose)

4 (neuropath* or angiopath* or retinopath* or atherosclero* or impaired vision or amput* or (renal and (damage or failure or impair*)) or (non-healing and ulcer) or stroke* or cerebrovascular accident* or (brain adj3 isch?emia))

5 exp Chronic Disease/ or Noncommunicable Diseases/
6 exp Cardiomyopathies/ or exp Heart Failure/ or exp Myocardial Ischemia/ or exp Ventricular Dysfunction/ or exp Arterial Occlusive Diseases/ or exp Diabetic Angiopathies/ or exp Hypertension/ or exp Peripheral Vascular Diseases/ or Prehypertension/ or exp Stroke/
7 exp Diabetes Mellitus/ or exp Hyperglycemia/
8 1 or 2 or 3 or 4 or 5 or 6 or 7

9 (humanitarian cris* or conflict or war or warfare or disaster* or earthquake* or hurricane* or cyclon* or flood* or landslide* or typhoon* or volcan* or tornado* or drought* or tsunami*)

10 (((relief or aid) adj3 (work or humanitarian or disaster)) or medical mission*)

11 (migrant* or refugee* or (displaced adj3 (person* or people or population* or communit*)))

12 Relief work/
13 exp Disasters/
14 Refugees/
15 exp Warfare and Armed Conflicts/
16 9 or 10 or 11 or 12 or 13 or 14 or 15

17 (((model? or package or community or home or facility or health or medical or clinic* or hospital or prevent* or primary or secondary or tertiary or referral or specialist) adj3 care) or healthcare or health service?)

18 (treatment* or screen* or referral* or management)

19 exp Behavior Control/ or exp Clinical Protocols/ or exp Drug Therapy/ or exp Emergency Treatment/ or exp Obesity Management/ or exp Nutrition therapy/ or exp Patient Care/ or exp Self Care/
20 exp Patient Care Management/
21 Models, Organizational/
22 exp General Practice/ or exp Preventive Medicine/
23 exp Community Health Services/ or Community Medicine/
24 17 or 18 or 19 or 20 or 21 or 22 or 23
25 8 and 16 and 24
26 limit 25 to yr="1990 – 2019"

27 Developing Countries/

28 ((developing or less* developed or under developed or underdeveloped or middle income or low* income) adj
(economy or economies)).ti,ab.

29 ((developing or less* developed or under developed or underdeveloped or middle income or low* income or
underserved or under served or deprived or poor*) adj (countr* or nation? or population? or world)).ti,ab.

30 (low* adj (gdp or gnp or gross domestic or gross national)).ti,ab.

31 (low adj3 middle adj3 countr*).ti,ab.

32 (lmic or lmics or third world or lmic countr*).ti,ab.

33 transitional countr*.ti,ab.

34 global south.ti,ab.

35 "Democratic People's Republic of Korea"/ or (North Korea or (Democratic People* Republic adj2 Korea)).ti,ab. or
Cambodia/ or Cambodia.ti,ab. or Indonesia/ or (Indonesia or Dutch East Indies).ti,ab. or (Kiribati or Gilbert Islands or
Phoenix Islands or Line Islands).ti,ab. or Laos/ or (Laos or (Lao adj1 Democratic Republic)).ti,ab. or Micronesia/ or
Micronesia.ti,ab. or Mongolia/ or Mongolia.ti,ab. or Myanmar/ or (Myanmar or Burma).ti,ab.

36 Papua New Guinea/ or (Papua New Guinea or German New Guinea or British New Guinea or Territory of Papua).ti,ab.
or Philippines/ or (Philippines or Philippine Islands).ti,ab. or Solomon Islands.ti,ab. or Timor-Leste/ or (Timor-Leste
or East Timor or Portuguese Timor).ti,ab. or Vanuatu/ or (Vanuatu or New Hebrides).ti,ab. or Vietnam/ or (Viet Nam or
Vietnam or French Indochina).ti,ab.

37 exp China/ or (China or People* Republic of China).ti,ab. or Fiji/ or Fiji.ti,ab. or Malaysia/ or (Malaysia or
Malayan Union or Malaya).ti,ab. or Marshall Islands.ti,ab. or Nauru.ti,ab. or "Independent State of Samoa"/ or ((Samoa
not American Samoa) or Western Samoa or Navigator Islands or Samoan Islands).ti,ab.

38 Thailand/ or (Thailand or Siam).ti,ab. or Tonga/ or Tonga.ti,ab. or (Tuvalu or Ellice Islands).ti,ab. or
Melanesia/ or Melanesia.ti,ab. or Polynesia/ or Polynesia.ti,ab. or Kyrgyzstan/ or (Kyrgyzstan or Kyrgyz Republic or
Kirghizia or Kirghiz).ti,ab.

39 (Moldova/ or Moldova.ti,ab. or Ukraine/ or Ukraine.ti,ab. or Uzbekistan/ or Uzbekistan.ti,ab. or Albania/ or
Albania.ti,ab. or Armenia/ or Armenia.ti,ab. or Azerbaijan/ or Azerbaijan.ti,ab. or "Republic of Belarus"/ or (Belarus
or Byelarus or Byelorussia or Belorussia).ti,ab. or Bosnia-Herzegovina/ or (Bosnia or Herzegovina).ti,ab. or Bulgaria/
or Bulgaria.ti,ab. or "Georgia (Republic)"/ or Georgia.ti,ab.) not Georgia/

40 Kazakhstan/ or (Kazakhstan or Kazakh).ti,ab. or Kosovo/ or Kosovo.ti,ab. or Montenegro/ or Montenegro.ti,ab. or
"Republic of North Macedonia"/ or North Macedonia.ti,ab. or Romania/ or Romania.ti,ab. or Russia/ or USSR/ or (Russia or
Russian Federation or USSR or Union of Soviet Socialist Republics or Soviet Union).ti,ab. or Serbia/ or Serbia.ti,ab. or
Turkey/ or (Turkey.ti,ab. not animal/) or (Anatolia or Asia Minor).ti,ab. or Turkmenistan/ or Turkmenistan.ti,ab. or
Tajikistan/ or Tajikistan.ti,ab. or Asia, Central/ or Asia, Northern/ or Central Asia.ti,ab.

41 Haiti/ or (Haiti or Hayti).ti,ab. or Bolivia/ or Bolivia.ti,ab. or El Salvador/ or El Salvador.ti,ab. or
Honduras/ or Honduras.ti,ab. or Nicaragua/ or Nicaragua.ti,ab. or Argentina/ or (Argentina or Argentine Republic).ti,ab.
or Belize/ or (Belize or British Honduras).ti,ab. or Brazil/ or Brazil.ti,ab. or Colombia/ or Colombia.ti,ab.

42 Costa Rica/ or Costa Rica.ti,ab. or Cuba/ or Cuba.ti,ab. or Dominica/ or Dominica.ti,ab. or Dominican Republic/
or Dominican Republic.ti,ab. or Ecuador/ or Ecuador.ti,ab. or Grenada/ or Grenada.ti,ab. or Guatemala/ or
Guatemala.ti,ab.

43 Guyana/ or (Guyana or British Guiana).ti,ab. or Jamaica/ or Jamaica.ti,ab. or Mexico/ or (Mexico or United
Mexican States).ti,ab. or Paraguay/ or Paraguay.ti,ab. or Peru/ or Peru.ti,ab. or Saint Lucia/ or (St Lucia or Saint
Lucia or Iyonala or Hewanorra).ti,ab. or "Saint Vincent and the Grenadines"/ or (Saint Vincent or St Vincent or
Grenadines).ti,ab. or Suriname/ or (Suriname or Dutch Guiana).ti,ab. or Venezuela/ or Venezuela.ti,ab.

44 Djibouti/ or (Djibouti or French Somaliland).ti,ab. or Egypt/ or Egypt.ti,ab. or Morocco/ or Morocco.ti,ab. or
Tunisia/ or Tunisia.mp. or (Gaza or West Bank or Palestine).ti,ab. or Algeria/ or Algeria.ti,ab. or Iran/ or (Iran or
Persia).ti,ab. or Iraq/ or (Iraq or Mesopotamia).ti,ab. or Jordan/ or Jordan.ti,ab. or Lebanon/ or (Lebanon or Lebanese
Republic).ti,ab. or Libya/ or Libya.ti,ab.

45 Syria/ or (Syria or Syrian Arab Republic).ti,ab. or Yemen/ or Yemen.ti,ab. or Afghanistan/ or Afghanistan.ti,ab.
or Nepal/ or Nepal.ti,ab. or Bangladesh/ or Bangladesh.ti,ab. or Bhutan/ or Bhutan.ti,ab. or exp India/ or India.ti,ab.
or Pakistan/ or Pakistan.ti,ab. or Maldives.ti,ab. or Sri Lanka/ or (Sri Lanka or Ceylon).ti,ab.

46 Angola/ or Angola.ti,ab. or Cameroon/ or (Cameroon or Kamerun or Cameroun).ti,ab. or Cape Verde/ or (Cape Verde
or Cabo Verde).ti,ab. or Comoros/ or (Comoros or Glorioso Islands or Mayotte).ti,ab. or Congo/ or (Congo not
((Democratic Republic adj3 Congo) or congo red or crimean-congo)).ti,ab. or Cote d'Ivoire/ or (Cote d'Ivoire or Cote
dIvoire or Ivory Coast).ti,ab. or Eswatini/ or (eSwatini or Swaziland).ti,ab. or Ghana/ or (Ghana or Gold Coast).ti,ab.

47 Kenya/ or (Kenya or East Africa Protectorate).ti,ab. or Lesotho/ or (Lesotho or Basutoland).ti,ab. or Mauritania/
or Mauritania.ti,ab. or Nigeria/ or Nigeria.ti,ab. or (Sao Tome adj2 Principe).ti,ab. or Senegal/ or Senegal.ti,ab. or
Sudan/ or (Sudan not South Sudan).ti,ab. or Zambia/ or (Zambia or Northern Rhodesia).ti,ab. or Zimbabwe/ or (Zimbabwe or
Southern Rhodesia).ti,ab.

48 Botswana/ or (Botswana or Bechuanaland or Kalahari).ti,ab. or Equatorial Guinea/ or (Equatorial Guinea or Spanish
Guinea).ti,ab. or Gabon/ or (Gabon or Gabonese Republic).ti,ab. or Mauritius/ or (Mauritius or Agalega Islands).ti,ab.
or Namibia/ or (Namibia or German South West Africa).ti,ab. or South Africa/ or (South Africa or Zululand or Transvaal
or Natalia Republic or Orange Free State).ti,ab. or Benin/ or (Benin or Dahomey).ti,ab. or Burkina Faso/ or (Burkina
Faso or Burkina Fasso or Upper Volta).ti,ab. or Burundi/ or (Burundi or Ruanda-Urundi).ti,ab.

49 Central African Republic/ or (Central African Republic or Ubangi-Shari).ti,ab. or Chad/ or Chad.ti,ab. or
"Democratic Republic of the Congo"/ or (((Democratic Republic or DR) adj2 Congo) or Congo-Kinshasa or Belgian Congo or
Zaire or Congo Free State).ti,ab. or Eritrea/ or Eritrea.ti,ab. or Ethiopia/ or (Ethiopia or Abyssinia).ti,ab. or
Gambia/ or Gambia.ti,ab. or Guinea/ or (Guinea not (New Guinea or Guinea Pig* or Guinea Fowl or Guinea-Bissau or
Portuguese Guinea or Equatorial Guinea)).ti,ab. or Guinea-Bissau/ or (Guinea-Bissau or Portuguese Guinea).ti,ab.

50 Liberia/ or Liberia.ti,ab. or Madagascar/ or (Madagascar or Malagasy Republic).ti,ab. or Malawi/ or (Malawi or
Nyasaland).ti,ab. or Mali/ or Mali.ti,ab. or Mozambique/ or (Mozambique or Mocambique or Portuguese East Africa).ti,ab.
or Niger/ or (Niger not (Aspergillus or Peptococcus or Schizothorax or Cruciferae or Gobius or Lasius or Agelastes or
Melanosuchus or radish or Parastromateus or Orius or Apergillus or Parastromateus or Stomoxys)).ti,ab.

51 Rwanda/ or (Rwanda or Ruanda).ti,ab. or Sierra Leone/ or (Sierra Leone or Salone).ti,ab. or Somalia/ or (Somalia
or Somaliland).ti,ab. or South Sudan/ or South Sudan.ti,ab. or Tanzania/ or (Tanzania or Tanganyika or Zanzibar).ti,ab.
or Togo/ or (Togo or Togolese Republic or Togoland).ti,ab. or Uganda/ or Uganda.ti,ab.

52 "africa south of the sahara"/ or africa, central/ or africa, eastern/ or africa, southern/ or africa, western/ or
("Africa South of the Sahara" or sub-Saharan Africa or subSaharan Africa).ti,ab. or Central Africa.ti,ab. or Eastern
Africa.ti,ab. or Southern Africa.ti,ab. or Western Africa.ti,ab.

53 or/27-52

54 26 and 53 (1446)

GLOBAL HEALTH AND ECON LIT – 23/11/2020

1. (non?communicable disease* or chronic disease* or chronic illness* or chronic condition* or long?term condition*).mp. [mp=abstract, title, original title, broad terms, heading words, identifiers, cabicodes]

2. (hypertensi* or high blood pressure or cardiovascular disease* or heart disease* or cardiomyopath* or ((heart or cardiac or ventricular) and (isch?emia or attack or failure or dysfunction or insufficiency))).mp. [mp=abstract, title, original title, broad terms, heading words, identifiers, cabicodes]

3. (diabet* or hyperglyc?emia or blood sugar or blood glucose).mp. [mp=abstract, title, original title, broad terms, heading words, identifiers, cabicodes]

4. (neuropath* or angiopath* or retinopath* or atherosclero* or impaired vision or amput* or (renal and (damage or failure or impair*)) or (non-healing and ulcer) or stroke* or cerebrovascular accident* or (brain adj3 isch?emia)).mp. [mp=abstract, title, original title, broad terms, heading words, identifiers, cabicodes]

5. exp chronic disease/ or exp noncommunicable diseases/

6. exp Cardiomyopathies/ or exp Heart Failure/ or exp Myocardial Ischemia/ or exp Ventricular Dysfunction/ or exp Arterial Occlusive Diseases/ or exp Diabetic Angiopathies/ or exp Hypertension/ or exp Peripheral Vascular Diseases/ or Prehypertension/ or exp Stroke/

7. exp Diabetes Mellitus/ or exp Hyperglycemia/

8. 1 or 2 or 3 or 4 or 5 or 6 or 7

9. (humanitarian cris* or conflict or war or warfare or disaster* or earthquake* or hurricane* or cyclon* or flood* or landslide* or typhoon* or volcan* or tornado* or drought* or tsunami*).mp. [mp=abstract, title, original title, broad terms, heading words, identifiers, cabicodes]

10. (((relief or aid) adj3 (work or humanitarian or disaster)) or medical mission*).mp. [mp=abstract, title, original title, broad terms, heading words, identifiers, cabicodes]

11. (migrant* or refugee* or (displaced adj3 (person* or people or population* or communit*))).mp. [mp=abstract, title, original title, broad terms, heading words, identifiers, cabicodes]

12. Relief work/

13. exp Disasters/

14. Refugees/

15. exp War/ and Armed Conflicts/

16. 9 or 10 or 11 or 12 or 13 or 14 or 15

17. (((model? or package or community or home or facility or health or medical or clinic* or hospital or prevent* or primary or secondary or tertiary or referral or specialist) adj3 care) or healthcare or health service?).mp. [mp=abstract, title, original title, broad terms, heading words, identifiers, cabicodes]

18. (treatment* or screen* or referral* or management).mp. [mp=abstract, title, original title, broad terms, heading words, identifiers, cabicodes]

19. exp Behavior Control/ or exp Clinical Protocols/ or exp Drug Therapy/ or exp Emergency

Treatment/ or exp Obesity Management/ or exp Nutrition therapy/ or exp Patient Care/ or exp Self Care/

20. exp Patient Care/

21. Models, Organizational/

22. exp General Practice/ or exp Preventive Medicine/

23. exp Community Health Services/ or Community Medicine/

24. 17 or 18 or 19 or 20 or 21 or 22 or 23

25. 8 and 16 and 24

26. 25

27. Developing Countries/

28. ((developing or less* developed or under developed or underdeveloped or middle income or low* income) adj (economy or economies)).ti,ab.

29. ((developing or less* developed or under developed or underdeveloped or middle income or low* income or underserved or under served or deprived or poor*) adj (countr* or nation? or population? or world)).ti,ab.

30. (low* adj (gdp or gnp or gross domestic or gross national)).ti,ab.

31. (low adj3 middle adj3 countr*).ti,ab.

32. (lmic or lmics or third world or lmic countr*).ti,ab.

33. transitional countr*.ti,ab.

34. global south.ti,ab.

35. "Democratic People's Republic of Korea"/ or (North Korea or (Democratic People* Republic adj2 Korea)).ti,ab. or Cambodia/ or Cambodia.ti,ab. or Indonesia/ or (Indonesia or Dutch East Indies).ti,ab. or (Kiribati or Gilbert Islands or Phoenix Islands or Line Islands).ti,ab. or Laos/ or (Laos or (Lao adj1 Democratic Republic)).ti,ab. or Micronesia/ or Micronesia.ti,ab. or Mongolia/ or Mongolia.ti,ab. or Myanmar/ or (Myanmar or Burma).ti,ab.

36. Papua New Guinea/ or (Papua New Guinea or German New Guinea or British New Guinea or Territory of Papua).ti,ab. or Philippines/ or (Philippines or Philippine Islands).ti,ab. or Solomon Islands.ti,ab. or Timor-Leste/ or (Timor-Leste or East Timor or Portuguese Timor).ti,ab. or Vanuatu/ or (Vanuatu or New Hebrides).ti,ab. or Vietnam/ or (Viet Nam or Vietnam or French Indochina).ti,ab.

37. exp China/ or (China or People* Republic of China).ti,ab. or Fiji/ or Fiji.ti,ab. or Malaysia/ or (Malaysia or Malayan Union or Malaya).ti,ab. or Marshall Islands.ti,ab. or Nauru.ti,ab. or "Independent State of Samoa"/ or ((Samoa not American Samoa) or Western Samoa or Navigator Islands or Samoan Islands).ti,ab.

38. Thailand/ or (Thailand or Siam).ti,ab. or Tonga/ or Tonga.ti,ab. or (Tuvalu or Ellice Islands).ti,ab. or Melanesia/ or Melanesia.ti,ab. or Polynesia/ or Polynesia.ti,ab. or Kyrgyzstan/ or (Kyrgyzstan or Kyrgyz Republic or Kirghizia or Kirghiz).ti,ab.

39. (Moldova/ or Moldova.ti,ab. or Ukraine/ or Ukraine.ti,ab. or Uzbekistan/ or Uzbekistan.ti,ab. or Albania/ or Albania.ti,ab. or Armenia/ or Armenia.ti,ab. or Azerbaijan/ or Azerbaijan.ti,ab. or "Republic of Belarus"/ or (Belarus or Byelarus or Byelorussia or Belorussia).ti,ab. or Bosnia-Herzegovina/ or (Bosnia or Herzegovina).ti,ab. or Bulgaria/ or Bulgaria.ti,ab. or "Georgia (Republic)"/ or Georgia.ti,ab.) not Georgia/

40. Kazakhstan/ or (Kazakhstan or Kazakh).ti,ab. or Kosovo/ or Kosovo.ti,ab. or Montenegro/ or Montenegro.ti,ab. or "Republic of North Macedonia"/ or North Macedonia.ti,ab. or Romania/ or Romania.ti,ab. or Russia/ or USSR/ or (Russia or Russian Federation or USSR or Union of Soviet Socialist Republics or Soviet Union).ti,ab. or Serbia/ or Serbia.ti,ab. or Turkey/ or (Turkey.ti,ab. not animal/) or (Anatolia or Asia Minor).ti,ab. or Turkmenistan/ or Turkmenistan.ti,ab. or Tajikistan/ or Tajikistan.ti,ab. or Asia, Central/ or Asia, Northern/ or Central Asia.ti,ab.

41. Haiti/ or (Haiti or Hayti).ti,ab. or Bolivia/ or Bolivia.ti,ab. or El Salvador/ or El Salvador.ti,ab. or Honduras/ or Honduras.ti,ab. or Nicaragua/ or Nicaragua.ti,ab. or Argentina/ or (Argentina or Argentine Republic).ti,ab. or Belize/ or (Belize or British Honduras).ti,ab. or Brazil/ or Brazil.ti,ab. or Colombia/ or Colombia.ti,ab.

42. Costa Rica/ or Costa Rica.ti,ab. or Cuba/ or Cuba.ti,ab. or Dominica/ or Dominica.ti,ab. or Dominican Republic/ or Dominican Republic.ti,ab. or Ecuador/ or Ecuador.ti,ab. or Grenada/ or Grenada.ti,ab. or Guatemala/ or Guatemala.ti,ab.

43. Guyana/ or (Guyana or British Guiana).ti,ab. or Jamaica/ or Jamaica.ti,ab. or Mexico/ or (Mexico or United Mexican States).ti,ab. or Paraguay/ or Paraguay.ti,ab. or Peru/ or Peru.ti,ab. or Saint Lucia/ or (St Lucia or Saint Lucia or Iyonala or Hewanorra).ti,ab. or "Saint Vincent and the Grenadines"/ or (Saint Vincent or St Vincent or Grenadines).ti,ab. or Suriname/ or (Suriname or Dutch Guiana).ti,ab. or Venezuela/ or Venezuela.ti,ab.

44. Djibouti/ or (Djibouti or French Somaliland).ti,ab. or Egypt/ or Egypt.ti,ab. or Morocco/ or Morocco.ti,ab. or Tunisia/ or Tunisia.mp. or (Gaza or West Bank or Palestine).ti,ab. or Algeria/ or Algeria.ti,ab. or Iran/ or (Iran or Persia).ti,ab. or Iraq/ or (Iraq or Mesopotamia).ti,ab. or Jordan/ or Jordan.ti,ab. or Lebanon/ or (Lebanon or Lebanese Republic).ti,ab. or Libya/ or Libya.ti,ab.

45. Syria/ or (Syria or Syrian Arab Republic).ti,ab. or Yemen/ or Yemen.ti,ab. or Afghanistan/ or Afghanistan.ti,ab. or Nepal/ or Nepal.ti,ab. or Bangladesh/ or Bangladesh.ti,ab. or Bhutan/ or Bhutan.ti,ab. or exp India/ or India.ti,ab. or Pakistan/ or Pakistan.ti,ab. or Maldives.ti,ab. or Sri Lanka/ or (Sri Lanka or Ceylon).ti,ab.

46. Angola/ or Angola.ti,ab. or Cameroon/ or (Cameroon or Kamerun or Cameroun).ti,ab. or Cape Verde/ or (Cape Verde or Cabo Verde).ti,ab. or Comoros/ or (Comoros or Glorioso Islands or Mayotte).ti,ab. or Congo/ or (Congo not ((Democratic Republic adj3 Congo) or congo red or crimean-congo)).ti,ab. or Cote d'Ivoire/ or (Cote d'Ivoire or Cote dIvoire or Ivory Coast).ti,ab. or Eswatini/ or (eSwatini or Swaziland).ti,ab. or Ghana/ or (Ghana or Gold Coast).ti,ab.

47. Kenya/ or (Kenya or East Africa Protectorate).ti,ab. or Lesotho/ or (Lesotho or Basutoland).ti,ab. or Mauritania/ or Mauritania.ti,ab. or Nigeria/ or Nigeria.ti,ab. or (Sao Tome adj2 Principe).ti,ab. or Senegal/ or Senegal.ti,ab. or Sudan/ or (Sudan not South Sudan).ti,ab. or Zambia/ or (Zambia or Northern Rhodesia).ti,ab. or Zimbabwe/ or (Zimbabwe or Southern Rhodesia).ti,ab.

48. Botswana/ or (Botswana or Bechuanaland or Kalahari).ti,ab. or Equatorial Guinea/ or (Equatorial Guinea or Spanish Guinea).ti,ab. or Gabon/ or (Gabon or Gabonese Republic).ti,ab. or Mauritius/ or (Mauritius or Agalega Islands).ti,ab. or Namibia/ or (Namibia or German South West Africa).ti,ab. or South Africa/ or (South Africa or Zululand or Transvaal or Natalia Republic or Orange Free State).ti,ab. or Benin/ or (Benin or Dahomey).ti,ab. or Burkina Faso/ or (Burkina Faso or Burkina Fasso or Upper Volta).ti,ab. or Burundi/ or (Burundi or Ruanda-Urundi).ti,ab.

49. Central African Republic/ or (Central African Republic or Ubangi-Shari).ti,ab. or Chad/ or Chad.ti,ab. or "Democratic Republic of the Congo"/ or (((Democratic Republic or DR) adj2 Congo) or Congo-Kinshasa or Belgian Congo or Zaire or Congo Free State).ti,ab. or Eritrea/ or Eritrea.ti,ab. or Ethiopia/ or (Ethiopia or Abyssinia).ti,ab. or Gambia/ or Gambia.ti,ab. or Guinea/ or (Guinea not (New Guinea or Guinea Pig* or Guinea Fowl or Guinea-Bissau or Portuguese Guinea or Equatorial Guinea)).ti,ab. or Guinea-Bissau/ or (Guinea-Bissau or Portuguese Guinea).ti,ab.

50. Liberia/ or Liberia.ti,ab. or Madagascar/ or (Madagascar or Malagasy Republic).ti,ab. or Malawi/ or (Malawi or Nyasaland).ti,ab. or Mali/ or Mali.ti,ab. or Mozambique/ or (Mozambique or Mocambique or Portuguese East Africa).ti,ab. or Niger/ or (Niger not (Aspergillus or Peptococcus or Schizothorax or Cruciferae or Gobius or Lasius or Agelastes or Melanosuchus or radish or Parastromateus or Orius or Apergillus or Parastromateus or Stomoxys)).ti,ab.

51. Rwanda/ or (Rwanda or Ruanda).ti,ab. or Sierra Leone/ or (Sierra Leone or Salone).ti,ab. or Somalia/ or (Somalia or Somaliland).ti,ab. or South Sudan/ or South Sudan.ti,ab. or Tanzania/ or (Tanzania or Tanganyika or Zanzibar).ti,ab. or Togo/ or (Togo or Togolese Republic or Togoland).ti,ab. or Uganda/ or Uganda.ti,ab.

52. "africa south of the sahara"/ or africa, central/ or africa, eastern/ or africa, southern/ or africa, western/ or ("Africa South of the Sahara" or sub-Saharan Africa or subSaharan Africa).ti,ab. or Central Africa.ti,ab. or Eastern Africa.ti,ab. or Southern Africa.ti,ab. or Western Africa.ti,ab.

53. or/27-52

54. 26 and 53

55. limit 54 to yr="1990 - 2019"

Import total:

EMBASE

1 (“non$communicable disease?” or “chronic disease?” or “chronic illness$” or “chronic condition?” or “long$term condition?”)

2 (hypertensi$ or “high blood pressure” or “cardiovascular disease?” or “heart disease?” or cardiomyopath$ or ((heart or cardiac or ventricular) and (isch$emia or attack or failure or dysfunction or insufficiency)))

3 (diabet$ or hyperglyc$emia or “blood sugar” or “blood glucose”)

4 (neuropath$ or angiopath$ or retinopath$ or atherosclero$ or “impaired vision” or amput$ or (renal and (damage or failure or impair$)) or (non healing and ulcer) or stroke? or “cerebrovascular accident?” or “brain isch$emia”)

5 “Chronic Disease”/exp or “Noncommunicable Diseases”/de

6 “Cardiomyopathies”/exp or “Heart Failure”/exp or “Myocardial Ischemia”/exp or “Ventricular Dysfunction”/exp or “Arterial Occlusive Diseases”/exp or “Diabetic Angiopathies”/exp or “Hypertension”/exp or “Peripheral Vascular Diseases”/exp or Prehypertension/de or “Stroke”/exp

7 “Diabetes Mellitus”/exp or Hyperglycemia/exp

8 1 or 2 or 3 or 4 or 5 or 6 or 7 (5554705)

9 (“humanitarian cris$” or conflict? or war? or warfare or disaster? or earthquake? or hurricane? or cyclon$ or flood? or landslide? or typhoon? or volcan$ or tornado? or drought? or tsunami?)
10 ((relief or aid) NEAR/3 (work) or “medical mission?”)
11 (migrant? or refugee? or (displaced NEAR/3 (person? or people or population? or communit?)))
12 “Relief work”/de
13 Disasters/exp
14 Refugees/exp
15 "Warfare and Armed Conflicts"/exp

16 9 or 10 or 11 or 12 or 13 or 14 or 15 (688890)

17 (((model? or package or community or home or facility or health or medical or clinic? or hospital or prevent? or primary or secondary or tertiary or referral or specialist) NEAR/3 care) or healthcare)
18 (treatment? or screen$ or referral? or management)
19 “Behavior Control”/exp or “Clinical Protocols”/exp or “Drug Therapy”/exp or “Emergency Treatment”/exp or “Obesity Management”/exp or “Nutrition Therapy”/exp or “Patient Care”/exp or “Self Care”/exp
20 “Patient Care Management”/exp
21 “Models, Organizational”/de
22 “General Practice”/exp or “Preventive Medicine”/exp
23 “Community Health Services”/exp or “Community Medicine”/de

24 17 or 18 or 19 or 20 or 21 or 22 or 23 (8688360)
25 8 and 16 and 24 (58,684 → 47,583)
26 #25 [1990-2019]/py (43043)

‘Developing Countries’/de
(developing OR less$developed OR under$developed OR middle$income OR low$income) NEAR/2 econom$
(developing OR less$developed OR under$developed OR middle$income OR low$income) NEAR/2 countr$
(developing OR less$developed OR under$developed OR middle$income OR low$income) NEAR/2 nation?

low$ near/2 (gdp or gnp or ‘gross domestic’ or ‘gross national’)
(lmic or lmics or ‘third world’ or ‘lmic countr$’) or
‘transitional countr$’ or
‘global south’

‘North Korea’ or ‘Democratic People$ Republic of Korea’ or
Cambodia/de or
Cambodia or
Indonesia/de or
Indonesia or
Kiribati or
Laos/de or
(Laos or ‘Lao Democratic Republic’) or
Micronesia/de or
Micronesia or

Mongolia/de or
Mongolia or
Myanmar/de or
(Myanmar or Burma) or
‘Papua New Guinea’/de or
‘Papua New Guinea’ or
Philippines/de or
Philippines or
‘Solomon Islands’ or
Timor-Leste/de or
(Timor-Leste or ‘East Timor’ or ’Portuguese Timor’) or
Vanuatu/de or
Vanuatu or
Vietnam/de or
(‘Viet Nam’ or Vietnam) Or

China/exp or
(China or ‘People* Republic of China’) or
Fiji/de or
Fiji or
Malaysia/de or
Malaysia or
‘Marshall Islands’ or
Nauru or
‘Independent State of Samoa’/de or
Thailand/de or
(Thailand or Siam) or
Tonga/de or
Tonga or
(Tuvalu) or
Melanesia/de or
Melanesia or
Polynesia/de or
Polynesia or
Kyrgyzstan/de or

(Kyrgyzstan or ‘Kyrgyz Republic’ or Kirghizia or Kirghiz)

Moldova/de or
Moldova or
Ukraine/de or
Ukraine or
Uzbekistan/de or
Uzbekistan or
Albania/de or
Albania or
Armenia/de or
Armenia or
Azerbaijan/de or
Azerbaijan or
"Republic of Belarus"/de or
(Belarus or Byelarus or Byelorussia or Belorussia) or
Bosnia-Herzegovina/de or
(Bosnia or Herzegovina) or
Bulgaria/de or
Bulgaria or
"Georgia (Republic)"/de or

Kazakhstan/de or
(Kazakhstan or Kazakh) or
Kosovo/de or
Kosovo or
Montenegro/de or
Montenegro or
"Republic of North Macedonia"/de or
‘North Macedonia’ or
Romania/de or
Romania or
Russia/de or
(Russia or ‘Russian Federation’) or
Serbia/de or
Serbia.ti,ab or
Turkey/de or
Turkmenistan/de or
Turkmenistan or
Tajikistan/de or
Tajikistan or
‘Asia, Central’/de or
‘Asia, Northern’/de or
‘Central Asia’

Haiti/de or

(Haiti or Hayti) or
Bolivia/de or
Bolivia or
‘El Salvador’/de or
‘El Salvador’ or
Honduras/de or
Honduras or
Nicaragua/de or
Nicaragua or
Argentina/de or
(Argentina or ‘Argentine Republic’) or
Belize/de or
(Belize) or
Brazil/de or
Brazil or
Colombia/de or
Colombia or
‘Costa Rica’/de or
‘Costa Rica’ or
Cuba/de or
Cuba or
Dominica/de or
Dominica or
‘Dominican Republic’/de or
‘Dominican Republic’ or
Ecuador/de or
Ecuador or
Grenada/de or
Grenada or
Guatemala/de or
Guatemala or

Guyana/de or
(Guyana) or
Jamaica/de or
Jamaica or
Mexico/de or
(Mexico or ‘United Mexican States’) or
Paraguay/de or
Paraguay or
Peru/de or
Peru or
‘Saint Lucia’/de or
"Saint Vincent and the Grenadines"/de or
Suriname/de or
(Suriname or ‘Dutch Guiana’) or

Venezuela/de or
Venezuela or

Djibouti/de or
Djibouti or
Egypt/de or
Egypt or
Morocco/de or
Morocco or
Tunisia/de or
Tunisia or
(Gaza or 'West Bank' or Palestine) or
Algeria/de or
Algeria or
Iran/de or
(Iran or Persia) or
Iraq/de or
(Iraq or Mesopotamia) or
Jordan/de or
Jordan or
Lebanon/de or
(Lebanon or 'Lebanese Republic') or
Libya/de or
Libya or

Syria/de or
(Syria or 'Syrian Arab Republic') or
Yemen/de or
Yemen or
Afghanistan/de or
Afghanistan or
Nepal/de or
Nepal or
Bangladesh/de or
Bangladesh or
Bhutan/de or
Bhutan or
India/exp or
India or
Pakistan/de or
Pakistan or
Maldives or
'Sri Lanka'/de or
('Sri Lanka' or Ceylon) or

Angola/de or
Angola or
Cameroon/de or
(Cameroon or Kamerun or Cameroun) or
‘Cape Verde’/de or
(‘Cape Verde’ or ‘Cabo Verde’) or
Comoros/de or
(Comoros) or
Congo/de or
‘Republic of Congo’ or
‘Cote d$Ivoire’/de or
(‘Cote d$Ivoire’ or ‘Ivory Coast’) or
Eswatini/de or
(eSwatini or Swaziland) or
Ghana/de or
Ghana or

Kenya/de or
(Kenya) or
Lesotho/de or
(Lesotho or Basutoland) or
Mauritania/de or
Mauritania or
Nigeria/de or
Nigeria or
Senegal/de or
Senegal or
Sudan/de or
(Sudan) or
Zambia/de or
(Zambia) or
Zimbabwe/de or
(Zimbabwe) or

Botswana/de or
(Botswana or Bechuanaland) or
‘Equatorial Guinea’/de or
(‘Equatorial Guinea’) or
Gabon/de or
(Gabon or ‘Gabonese Republic’) or
Mauritius/de or
(Mauritius) or
Namibia/de or
(Namibia) or
‘South Africa’/de or
(‘South Africa’ or Zululand or Transvaal) or

Benin/de or
(Benin or Dahomey) or
‘Burkina Faso’/de or
(‘Burkina Faso’) or
Burundi/de or
(Burundi or ‘Ruanda-Urundi’) or

‘Central African Republic’/de or
(‘Central African Republic’) or
Chad/de or
Chad or
"Democratic Republic of the Congo"/de or
‘democratic republic of congo’ or
Eritrea/de or
Eritrea or
Ethiopia/de or
(Ethiopia or Abyssinia) or
Gambia/de or
Gambia or
Guinea/de or
(Guinea)
‘Guinea Bissau’/de or
(‘Guinea-Bissau’) or

Liberia/de or
Liberia or
Madagascar/de or
(Madagascar or ‘Malagasy Republic’) or
Malawi/de or
(Malawi or Nyasaland) or
Mali/de or
Mali or
Mozambique/de or
(Mozambique or Mocambique) or
Niger/de or
(Niger not (Aspergillus or Peptococcus or Schizothorax or Cruciferae or Gobius or Lasius or Agelastes or Melanosuchus or radish or Parastromateus or Orius or Apergillus or Parastromateus or Stomoxys)) or

Rwanda/de or
(Rwanda or Ruanda) or
‘Sierra Leone’/de or
(‘Sierra Leone’ or Salone) or
Somalia/de or
(Somalia or Somaliland) or
‘South Sudan’/de or

‘South Sudan’ or
Tanzania/de or
(Tanzania or Tanganyika or Zanzibar) or
Togo/de or
(Togo or ‘Togolese Republic’ or Togoland) or
Uganda/de or
Uganda or

"africa south of the sahara"/de or
‘africa, central’/de or
‘africa, eastern’/de or
‘africa, southern’/de or
‘africa, western’/de or
("Africa South of the Sahara" or ‘sub-Saharan Africa’ or ‘subSaharan Africa’) or
‘Central Africa’ or
‘Eastern Africa’ or
‘Southern Africa’ or
‘Western Africa’

WoS

TS = (“non$communicable disease” or “chronic disease$” or “chronic condition$” or “long$term condition”) or
TS = (hypertensi* or “high blood pressure” or “cardiovascular disease$” or “heart disease$”) or
TS = (diabet* or hyperglyc?emia or “blood sugar” or “blood glucose”) or
TS = (neuropath* or angiopath* or retinopathy* or atherosclero8 or (renal and (damage or failure or impair*)) or “non-healing ulcer” or “impaired vision” or amput*)

TS = (“humanitarian emergenc*” or “humanitarian cris*” conflict$ or war$ or warfare or disaster$ or “complex emergenc*” or typhoon$ or volcan* or tornado$ or drought$ or tsunami$ or “climate cris*” or “climate change”)
TS = (((relief or aid) near/3 work) or “medical mission$”) or
TS = (migrant or refugee or (displaced near/3 (person$ or people or population$ or communit*)))

TS = ((model$ or package or community or home or facility or health or medical or clinic* or hospital or prevent* or primary or secondary or tertiary or referral or specialist) near/3 care) or
TS = (treatment$ or screen* or referral or management) or
TS = (“behavior control” or “clinical protocols” or “patient care” or “organization model$” or “general practice” or “preventative medicine” or “community health service$” or or “community medicine”)

Import Total: 3912

GIM

tw:((tw:("noncommunicable disease" OR "chronic disease" OR "chronic illness" OR "chronic condition" OR diabet* OR hyperglycemia OR hypertens* OR "high blood pressure" OR "cardiovascular disease" OR “heart disease” OR neuropath* OR angiopath* OR retinopath* OR atherosclero* OR “impaired vision” OR amput* OR stroke)) AND (tw:("humanitarian emergency" OR “humanitarian crisis” OR conflict* OR war* OR warfare OR disaster* OR "complex emergency" OR earthquake* OR hurricane* OR cyclone* OR flood* OR landslide* OR typhoon* OR volcan* OR drought* OR tsunami* OR “climate crisis” OR “climate change” OR “relief work” OR “aid work” OR “medical mission” OR migrant* OR refugee* OR “displaced person”)) AND (tw:((“model of care” OR “package of care” OR “primary care” OR “secondary care” OR “health care” OR “clinical care” OR “hospital care”) OR (healthcare OR treatment* OR screen* OR referral* OR management) OR ("patient care management" OR "general practice" OR “behavior control” OR “clinical protocols” OR “drug therapy” OR “emergency treatment” OR “obesity management” OR “nutrition therapy” OR “patient care” OR “self care” OR “patient care management” OR “models, organizational” OR “preventative medicine” OR “community health services” OR “community medicine”)))) AND (fulltext:("1")) AND (year_cluster:[1990 TO 2019])

Import total: 4214

Total:

--
LMICS
Developing Countries/
((developing or less* developed or under developed or underdeveloped or middle income or low* income) adj (economy or economies)).ti,ab. (424)
((developing or less* developed or under developed or underdeveloped or middle income or low* income or underserved or under served or deprived or poor*) adj (countr* or nation? or population? or world)).ti,ab.
(low* adj (gdp or gnp or gross domestic or gross national)).ti,ab.
(low adj3 middle adj3 countr*).ti,ab.
(lmic or lmics or third world or lmic countr*).ti,ab.
transitional countr*.ti,ab.
global south.ti,ab.

"Democratic People's Republic of Korea"/ or
(North Korea or (Democratic People* Republic adj2 Korea)).ti,ab. or
Cambodia/ or
Cambodia.ti,ab. or
Indonesia/ or
(Indonesia or Dutch East Indies).ti,ab. or
(Kiribati or Gilbert Islands or Phoenix Islands or Line Islands).ti,ab or
Laos/ or

(Laos or (Lao adj1 Democratic Republic)).ti,ab. or
Micronesia/ or
Micronesia.ti,ab. or
Mongolia/ or
Mongolia.ti,ab. or
Myanmar/ or
(Myanmar or Burma).ti,ab. or
Papua New Guinea/ or
(Papua New Guinea or German New Guinea or British New Guinea or Territory of Papua).ti,ab. or
Philippines/ or
(Philippines or Philippine Islands).ti,ab. or
Solomon Islands.ti,ab. or
Timor-Leste/ or
(Timor-Leste or East Timor or Portuguese Timor).ti,ab. or
Vanuatu/ or
(Vanuatu or New Hebrides).ti,ab. or
Vietnam/ or
(Viet Nam or Vietnam or French Indochina).ti,ab. Or

exp China/ or
(China or People* Republic of China).ti,ab. or
Fiji/ or
Fiji.ti,ab. or
Malaysia/ or
(Malaysia or Malayan Union or Malaya).ti,ab. or
Marshall Islands.ti,ab. or
Nauru.ti,ab. or
"Independent State of Samoa"/ or
((Samoa not American Samoa) or Western Samoa or Navigator Islands or Samoan Islands).ti,ab. or
Thailand/ or
(Thailand or Siam).ti,ab. or
Tonga/ or
Tonga.ti,ab. or
(Tuvalu or Ellice Islands).ti,ab. or
Melanesia/ or
Melanesia.ti,ab. or
Polynesia/ or
Polynesia.ti,ab. or
Kyrgyzstan/ or
(Kyrgyzstan or Kyrgyz Republic or Kirghizia or Kirghiz).ti,ab.

Moldova/ or
Moldova.ti,ab. or
Ukraine/ or

Ukraine.ti,ab. or
Uzbekistan/ or
Uzbekistan.ti,ab. or
Albania/ or
Albania.ti,ab. or
Armenia/ or
Armenia.ti,ab. or
Azerbaijan/ or
Azerbaijan.ti,ab. or
"Republic of Belarus"/ or
(Belarus or Byelarus or Byelorussia or Belorussia).ti,ab. or
Bosnia-Herzegovina/ or
(Bosnia or Herzegovina).ti,ab. or
Bulgaria/ or
Bulgaria.ti,ab. or
"Georgia (Republic)"/ or
Georgia.ti,ab. not Georgia/ or

Kazakhstan/ or
(Kazakhstan or Kazakh).ti,ab. or
Kosovo/ or
Kosovo.ti,ab. or
Montenegro/ or
Montenegro.ti,ab. or
"Republic of North Macedonia"/ or
North Macedonia.ti,ab. or
Romania/ or
Romania.ti,ab or
Russia/ or
USSR/ or
(Russia or Russian Federation or USSR or Union of Soviet Socialist Republics or Soviet Union).ti,ab. or
Serbia/ or
Serbia.ti,ab or
Turkey/ or
(Turkey.ti,ab. not animal/) or (Anatolia or Asia Minor).ti,ab. or
Turkmenistan/ or
Turkmenistan.ti,ab. or
Tajikistan/ or
Tajikistan.ti,ab. or
Asia, Central/ or
Asia, Northern/ or
Central Asia.ti,ab.

Haiti/ or
(Haiti or Hayti).ti,ab. or

Bolivia/ or
Bolivia.ti,ab. or
El Salvador/ or
El Salvador.ti,ab. or
Honduras/ or
Honduras.ti,ab. or
Nicaragua/ or
Nicaragua.ti,ab. or
Argentina/ or
(Argentina or Argentine Republic).ti,ab. or
Belize/ or
(Belize or British Honduras).ti,ab. or
Brazil/ or
Brazil.ti,ab. or
Colombia/ or
Colombia.ti,ab. or
Costa Rica/ or
Costa Rica.ti,ab. or
Cuba/ or
Cuba.ti,ab or
Dominica/ or
Dominica.ti,ab or
Dominican Republic/ or
Dominican Republic.ti,ab. or
Ecuador/ or
Ecuador.ti,ab. or
Grenada/ or
Grenada.ti,ab. or
Guatemala/ or
Guatemala.ti,ab. or

Guyana/ or
(Guyana or British Guiana).ti,ab. or
Jamaica/ or
Jamaica.ti,ab. or
Mexico/ or
(Mexico or United Mexican States).ti,ab. or
Paraguay/ or
Paraguay.ti,ab. or
Peru/ or
Peru.ti,ab. or
Saint Lucia/ or
(St Lucia or Saint Lucia or Iyonala or Hewanorra).ti,ab. or
"Saint Vincent and the Grenadines"/ or
(Saint Vincent or St Vincent or Grenadines).ti,ab. or
Suriname/ or

(Suriname or Dutch Guiana).ti,ab. or
Venezuela/ or
Venezuela.ti,ab. or

Djibouti/ or
(Djibouti or French Somaliland).ti,ab. or
Egypt/ or
Egypt.ti,ab. or
Morocco/ or
Morocco.ti,ab. or
Tunisia/ or
Tunisia.mp. or
(Gaza or West Bank or Palestine).ti,ab. or
Algeria/ or
Algeria.ti,ab. or
Iran/ or
(Iran or Persia).ti,ab or
Iraq/ or
(Iraq or Mesopotamia).ti,ab. or
Jordan/ or
Jordan.ti,ab. or
Lebanon/ or
(Lebanon or Lebanese Republic).ti,ab. or
Libya/ or
Libya.ti,ab. or

Syria/ or
(Syria or Syrian Arab Republic).ti,ab. or
Yemen/ or
Yemen.ti,ab. or
Afghanistan/ or
Afghanistan.ti,ab. or
Nepal/ or
Nepal.ti,ab. or
Bangladesh/ or
Bangladesh.ti,ab or
Bhutan/ or
Bhutan.ti,ab or
exp India/ or
India.ti,ab or
Pakistan/ or
Pakistan.ti,ab. or
Maldives.ti,ab. or
Sri Lanka/ or
(Sri Lanka or Ceylon).ti,ab. or

Angola/ or
Angola.ti,ab. or
Cameroon/ or
(Cameroon or Kamerun or Cameroun).ti,ab. or
Cape Verde/ or
(Cape Verde or Cabo Verde).ti,ab. or
Comoros/ or
(Comoros or Glorioso Islands or Mayotte).ti,ab. or
Congo/ or
(Congo not ((Democratic Republic adj3 Congo) or congo red or crimean-congo)).ti,ab. or
Cote d'Ivoire/ or
(Cote d'Ivoire or Cote dIvoire or Ivory Coast).ti,ab. or
Eswatini/ or
(eSwatini or Swaziland).ti,ab or
Ghana/ or
(Ghana or Gold Coast).ti,ab or

Kenya/ or
(Kenya or East Africa Protectorate).ti,ab. or
Lesotho/ or
(Lesotho or Basutoland).ti,ab. or
Mauritania/ or
Mauritania.ti,ab. or
Nigeria/ or
Nigeria.ti,ab. or
(Sao Tome adj2 Principe).ti,ab. or
Senegal/ or
Senegal.ti,ab. or
Sudan/ or
(Sudan not South Sudan).ti,ab. or
Zambia/ or
(Zambia or Northern Rhodesia).ti,ab. or
Zimbabwe/ or
(Zimbabwe or Southern Rhodesia).ti,ab. or

Botswana/ or
(Botswana or Bechuanaland or Kalahari).ti,ab. or
Equatorial Guinea/ or
(Equatorial Guinea or Spanish Guinea).ti,ab. or
Gabon/ or
(Gabon or Gabonese Republic).ti,ab. or
Mauritius/ or
(Mauritius or Agalega Islands).ti,ab. or
Namibia/ or
(Namibia or German South West Africa).ti,ab. or

South Africa/ or
(South Africa or Zululand or Transvaal or Natalia Republic or Orange Free State).ti,ab. or
Benin/ or
(Benin or Dahomey).ti,ab. or
Burkina Faso/ or
(Burkina Faso or Burkina Fasso or Upper Volta).ti,ab or
Burundi/ or
(Burundi or Ruanda-Urundi).ti,ab. or

Central African Republic/ or
(Central African Republic or Ubangi-Shari).ti,ab. or
Chad/ or
Chad.ti,ab. or
"Democratic Republic of the Congo"/ or
(((Democratic Republic or DR) adj2 Congo) or Congo-Kinshasa or Belgian Congo or Zaire or Congo Free State).ti,ab. or
Eritrea/ or
Eritrea.ti,ab. or
Ethiopia/ or
(Ethiopia or Abyssinia).ti,ab. or
Gambia/ or
Gambia.ti,ab. or
Guinea/ or
(Guinea not (New Guinea or Guinea Pig* or Guinea Fowl or Guinea-Bissau or Portuguese Guinea or Equatorial Guinea)).ti,ab. or
Guinea-Bissau/ or
(Guinea-Bissau or Portuguese Guinea).ti,ab. or

Liberia/ or
Liberia.ti,ab or
Madagascar/ or
(Madagascar or Malagasy Republic).ti,ab. or
Malawi/ or
(Malawi or Nyasaland).ti,ab. or
Mali/ or
Mali.ti,ab. or
Mozambique/ or
(Mozambique or Mocambique or Portuguese East Africa).ti,ab. or
Niger/ or
(Niger not (Aspergillus or Peptococcus or Schizothorax or Cruciferae or Gobius or Lasius or Agelastes or Melanosuchus or radish or Parastromateus or Orius or Apergillus or Parastromateus or Stomoxys)).ti,ab. or

Rwanda/ or
(Rwanda or Ruanda).ti,ab. or
Sierra Leone/ or

(Sierra Leone or Salone).ti,ab. or
Somalia/ or
(Somalia or Somaliland).ti,ab. or
South Sudan/ or
South Sudan.ti,ab or
Tanzania/ or
(Tanzania or Tanganyika or Zanzibar).ti,ab. or
Togo/ or
(Togo or Togolese Republic or Togoland).ti,ab. or
Uganda/ or
Uganda.ti,ab. or

"africa south of the sahara"/ or
africa, central/ or
africa, eastern/ or
africa, southern/ or
africa, western/ or
("Africa South of the Sahara" or sub-Saharan Africa or subSaharan Africa).ti,ab. or
Central Africa.ti,ab. or
Eastern Africa.ti,ab. or
Southern Africa.ti,ab. or
Western Africa.ti,ab.
